# Regioselective bromination of pyrrolo[1,2-*a*]quinoxalines†

**DOI:** 10.1039/d4ra07358d

**Published:** 2024-11-15

**Authors:** Yingqian Li, Yali Liu, Di Hao, Liang Xu, Ping Liu

**Affiliations:** a School of Chemistry and Chemical Engineering, Key Laboratory for Green Processing of Chemical Engineering of Xinjiang Bingtuan, Shihezi University Shihezi 832003 China liuping@shzu.edu.cn liuping1979112@aliyun.com liuyan1979810@aliyun.com +86 0993 2057270 +86 0993 2057213

## Abstract

In this study, we report a novel and efficient method for the regioselective bromination of pyrrolo[1,2-*a*]quinoxalines using tetrabutylammonium tribromide (TBATB). This method exploits the mild nature of TBATB to obtain highly selective C3-brominated or C1, C3-dibrominated products in good yields. Notably, the reaction has a broad substrate applicability, and the C3-brominated product can be synthesized on a gram scale and can be further converted into structurally diverse pyrrolo[1,2-*a*]quinoxaline derivatives.

## Abstract 2

A novel and efficient method for the regioselective bromination of pyrrolo[1,2-*a*]quinoxaline using tetrabutylammonium tribromide was developed. A series of highly selective C3-brominated or C1, C3-dibrominated products were obtained in good yields.

## Graphical abstract

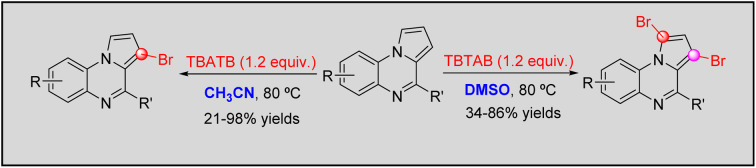

## Introduction

**Fig. 1:**
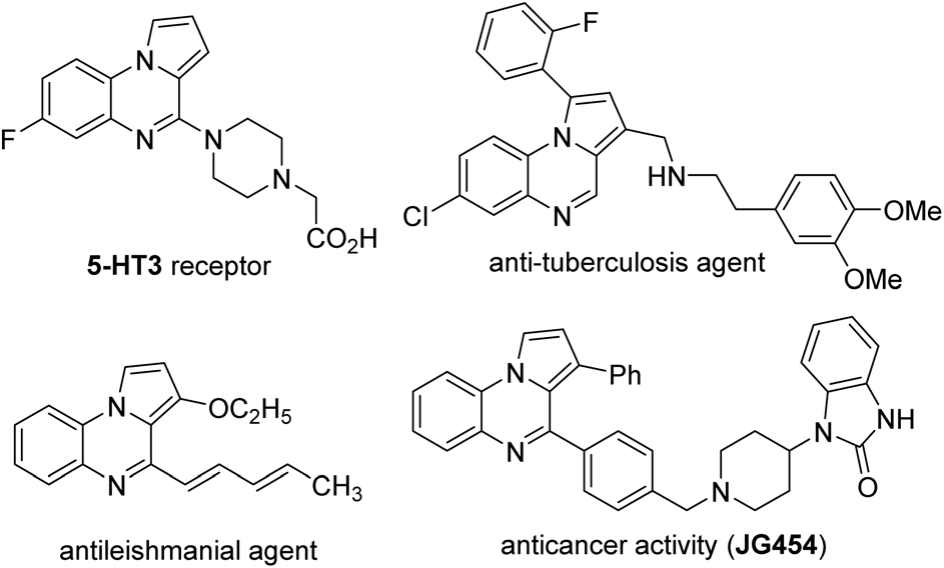
Pyrrolo[1,2-*a*]quinoxalines in biologically important targets.

**Scheme 1:**
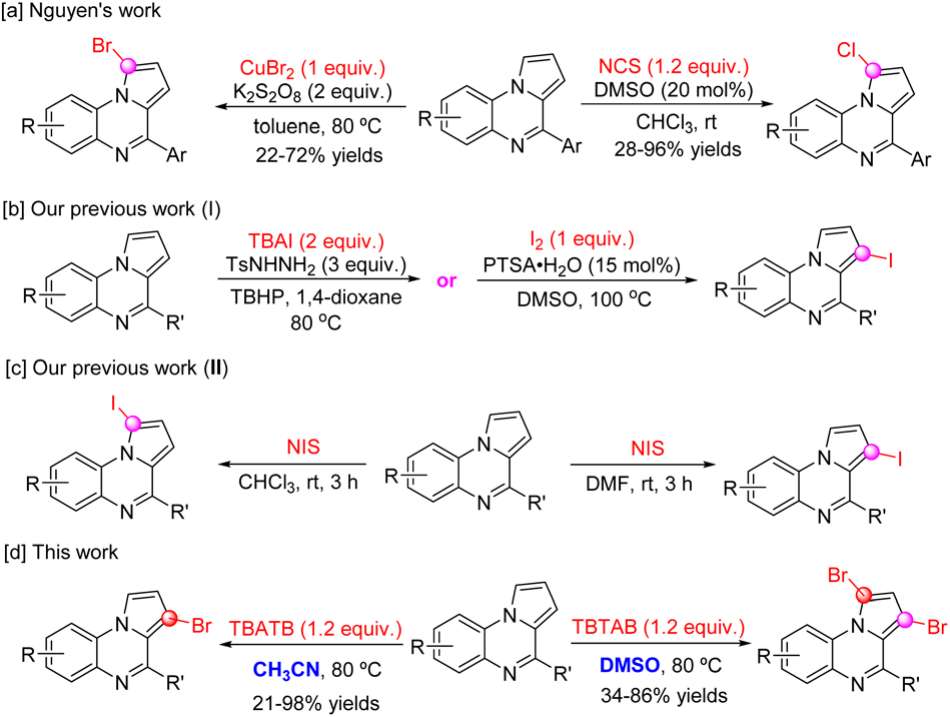
Selective halogenation of pyrrolo[1,2-*a*]quinoxaline.

Nitrogen heterocycles are important structural elements in medicinal chemistry. According to the U.S. Food and Drug Administration (FDA) approved drug database, more than half of the unique small molecule drugs contain a nitrogen ring structure.^1^ Pyrrolo[1,2-*a*]quinoxalines are an important class of nitrogen-containing heterocyclic compounds with great potential for applications in medicinal chemistry and materials science.^2–6^ They are commonly used to study inhibition of human protein kinase CK2, and anticancer, antimalarial, anti-HIV, antifungal, antidiabetic and antitubercular activities (Fig. 1). In addition, pyrrolo[1,2-*a*]quinoxaline has shown potential as a potential candidate material in the field of photovoltaic research due to its unique electrochemical and photochemical properties. Therefore, it is particularly important to construct diverse backbones for pyrrolo[1,2-*a*]quinoxaline derivatives. In the last decade, cyclization reactions have become the main strategy for the synthesis of pyrrolo[1,2-*a*]quinoxaline.^7^ However, these approaches are mainly limited to the construction of 4-substituted pyrrolo[1,2-*a*]quinoxaline skeletons, which largely limits the potential of pyrrolo[1,2-*a*]quinoxalines as bioactive molecules. Therefore, exploring new ways to enrich the structural diversity of pyrrolo[1,2-*a*]quinoxaline derivatives has become an important topic in synthetic chemistry. In recent years, C–H activation of (hetero)olefins has attracted much attention in the field of organic synthesis because of its directness and efficiency.^8^ As a heterocyclic molecule with multiple reaction sites, further structural modification of pyrrolo[1,2-*a*]quinoxaline is theoretically feasible. A series of preliminary work by our research group has also confirmed that the introduction of various functional groups on the pyrrolo[1,2-*a*]quinoxaline backbone through direct C–H functionalization is a feasible strategy.^9–13^ These include C1-thiocyanation, selenocyanation, C1-amidation, and C1/C3-arylation of pyrrolo[1,2-*a*]quinoxaline, in addition, halogenation of pyrrolo[1,2-*a*]quinoxaline has also been explored, as this approach provides a viable route for the late functionalization of pyrrolo[1,2-*a*]quinoxaline.^14–16^ For example, Nguyen *et al.* developed a C1-bromination of pyrrolo[1,2-*a*]quinoxaline, which was carried out with CuBr_2_ as the bromination reagent and K_2_S_2_O_8_ as the oxidizing agent. Similarly, the C1-chlorination of 4-arylpyrrolo[1,2-*a*]quinoxalines was carried out in the presence of NCS and catalytic amounts of DMSO (Scheme 1a).^14^ Recently, we described a direct C3-iodination of pyrrolo[1,2-*a*]quinoxalines with TBAI or I_2_, a series of novel 3-iodo-pyrrolo[1,2-*a*]quinoxalines were obtained with excellent regioselectivity (Scheme 1b).^15^ In addition, we reported a solvent mediated regioselective C–H iodination of pyrrolo[1,2-*a*]quinoxaline and NIS, and C1-iodo or 3-iodopyrrolo[1,2-*a*]quinoxalines could be selectively synthesized by using CHCl_3_ and DMF as solvents, respectively (Scheme 1c).^15^ However, to the best of our knowledge, C3-bromination for pyrrolo[1,2-*a*]quinoxaline has not been realized as of this date.

In recent years, tetrabutylammonium tribromide (TBATB) has emerged as a powerful bromination reagent in a variety of reactions.^17^ The benefits of TBATB include a higher safety profile, ease of handling, and reduced environmental impact compared to conventional brominating agents such as molecular bromine (Br_2_).^18^ The application of TBATB offers a range of mild and efficient bromination routes that are compatible with a wide range of functional and sensitive groups. Our hypothesis is based on the ability of TBATB to slow-release monomeric bromine and the regioelectronic effect of pyrrolo[1,2-*a*]quinoxaline, resulting in selective access to novel brominated pyrrolo[1,2-*a*]quinoxalines (Scheme 1d). This approach should provide a highly selective process that is simple to operate and functional group tolerant, providing a valuable addition to existing bromination methods. We herein describe the feasibility of this hypothesis.

## Results and discussion

### Optimization of the reaction conditions

**Fig. 2:**
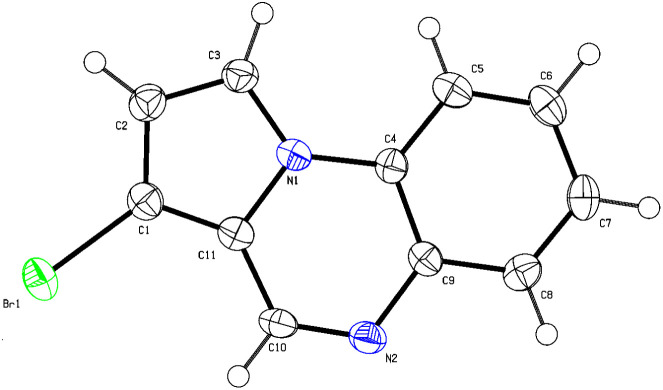
The crystal structure of 3a.

First, this hypothesis was tested by adding 1 equivalent of TBATB to a dimethyl sulfoxide (DMSO) solution of pyrrolo[1,2-*a*]quinoxaline (1a, 0.2 mmol) at 60 °C (Table 1). The results showed that the bromination was able to proceed smoothly and yielded 53% of the C3-brominated product 3a and 28% of the 1,3-dibrominated product 4a (entry 1). The structure of 3a was confirmed by X-single crystal diffraction analysis (Fig. 2. CCDC: 2388555†). Increasing the temperature to 80 °C resulted in a significant increase in the selectivity of 4a (entry 2). A further increase to 100 °C was detrimental to both the conversion and selectivity of the reaction (entry 3). We then examined the effect of the amount of 2a and showed that both the conversion of the reaction and the selectivity of 4a were significantly improved when 1.2 equivalents of 2a was used (entries 4 and 5). To improve the selectivity of 3a, we examined the effect of various reaction solvents. The results indicated that EtOAc and EtOH were ineffective (entries 6 and 7), in contrast to DMA, DMF, and MeCN, which performed well, especially when MeCN was used as the reaction solvent, with yields of 3a reaching 94% (entries 8–10).

**Table 1:** Optimized reaction conditions^a^

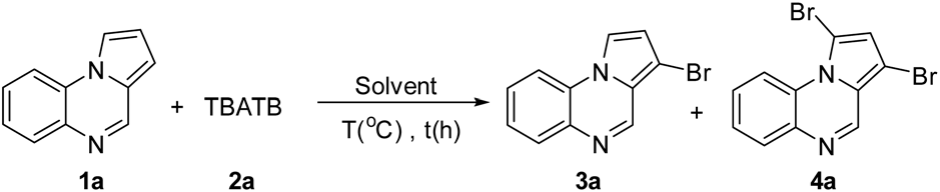						
Entry	1a (mmol)	2a (mmol)	Solvent	*T* (°C)	Yield^b^ (%)	
					3a	4a
1	0.2	0.2	DMSO	60	53	28
2	0.2	0.2	DMSO	80	15	73
3	0.2	0.2	DMSO	100	10	49
4	**0.2**	**0.24**	**DMSO**	**80**	**18**	**80**
5	0.2	0.3	DMSO	80	6	69
6	0.2	0.24	EtOAc	80	22	8
7	0.2	0.24	EtOH	80	54	9
8	0.2	0.24	DMA	80	61	20
9	0.2	0.24	DMF	80	77	18
10	**0.2**	**0.24**	MeCN	**80**	**94**	**5**

a Reaction conditions: 1a (0.2 mmol), 2a (0.24 mmol), solvent (2.5 mL), 12 h.

b Isolated yield.

### Scope and limitations of substrates

**Scheme 2:**
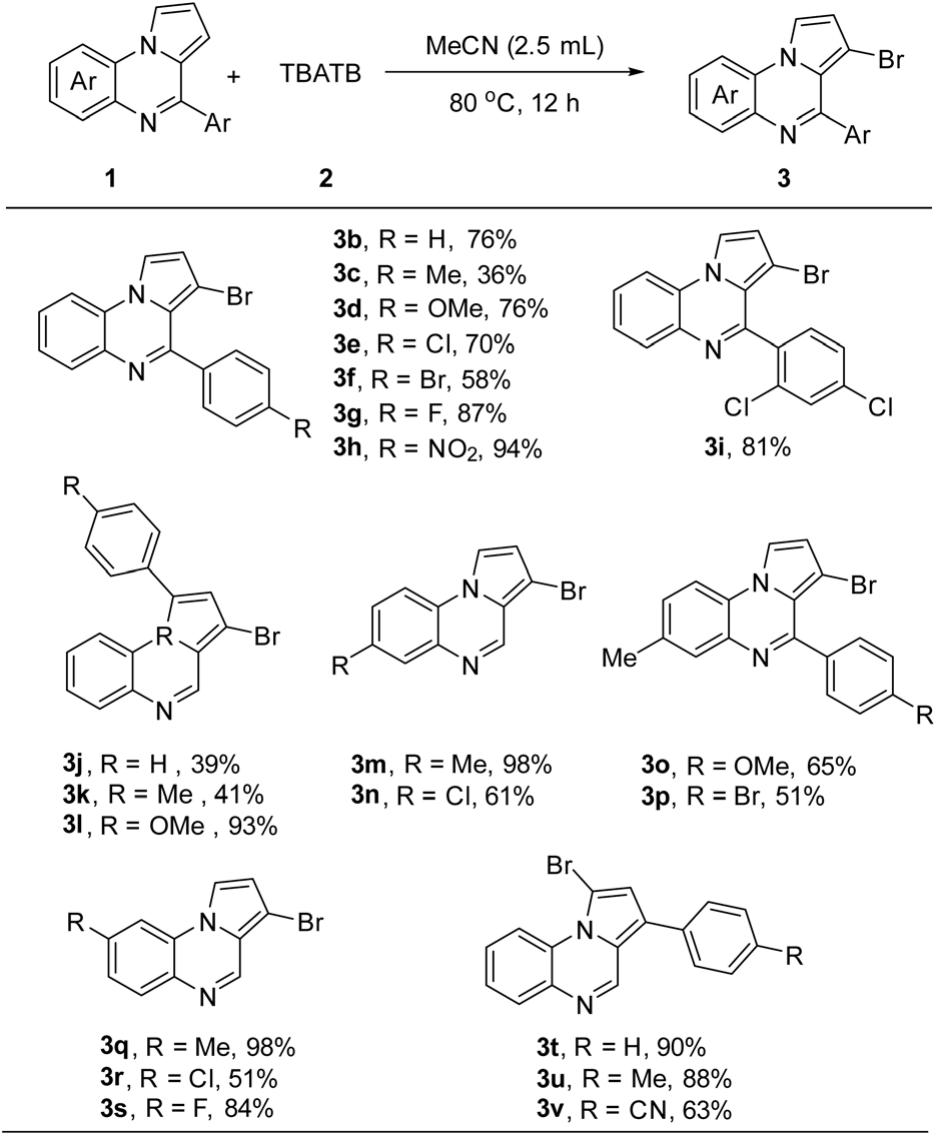
C3-bromination of pyrrolo[1,2-*a*]quinoxalines^*a*^. ^*a*^Reaction conditions: 1 (0.2 mmol), 2 (0.24 mmol), solvent (2.5 mL), 80 °C, isolated yield.

Next, we investigated the substrate scope of pyrrolo[1,2-*a*]quinoxaline 1 as described in Scheme 2. The experimental results showed that the 4-aryl-substituted pyrrolo[1,2-*a*]quinoxalines exhibited good reactivity in most of the cases, and the desired C3-brominated products 3b–3i were successfully obtained. Particularly, the reaction exhibited good compatibility with a variety of aryl groups, wherein the reactions with electron-withdrawing groups (*e.g.*, 4-OMe, 4-Cl, 4-Br, 4-F, 4-NO_2_ and 2,4-dichloro groups) exhibited superior reactivity compared to those with electron-donating groups (*e.g.*, 4-Me). Similarly, for 1-aryl substituted pyrrolo[1,2-*a*]quinoxaline substrates, we observed a similar reaction pattern (3i–3l). However, an interesting inversion of the reactivity was observed when substituents were present at the 6- and 7-positions on the benzene ring of the pyrrolo[1,2-*a*]quinoxalines, when the electron-donating group seemed to be more favorable for the C3-bromination (3m–3s). It was also noteworthy that when the C3-position of the pyrrolo[1,2-*a*]quinoxaline was occupied by an aryl group, the bromination reaction will take place on C1-position, and we succeeded in obtaining the products 3t–3v in yields of 63–90%.

**Scheme 3:**
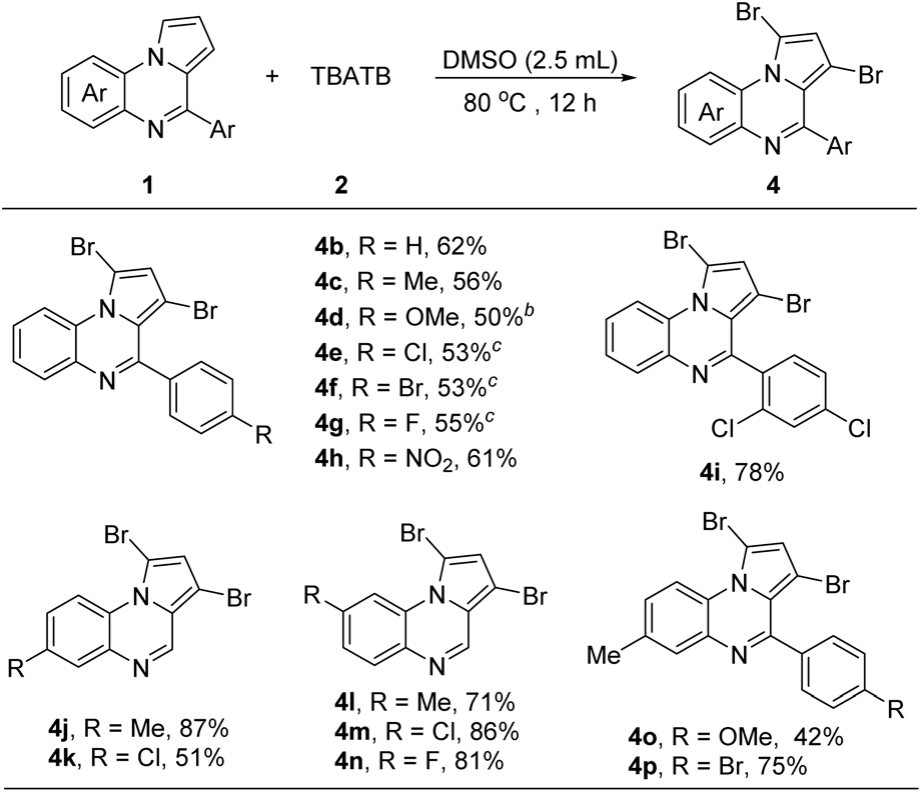
1,3-Dibromination of pyrrolo[1,2-*a*]quinoxaline with TBATB^*a*^. ^*a*^Reaction conditions: 1 (0.2 mmol), 2 (0.24 mmol), solvent (2.5 mL), 80 °C, isolated yield; ^*b*^0.3 mmol of 2; ^*c*^0.4 mmol of 2, 17 h.

We also examined the efficiency of the 1,3-dibromination reaction of pyrrolo[1,2-*a*]quinoxaline derivatives with TBATB (Scheme 3). Various substituted pyrrolo[1,2-*a*]quinoxaline were obtained in moderate to good yields to the 1,3-dibrominated products 4b–4p. Various groups such as F^−^, Cl^−^, Br^−^, Me^−^, MeO^−^, and nitro were tolerated. In most examples, 4-aryl-substituted substrates containing electron-withdrawing groups typically exhibit good reactivity, providing the products in satisfactory yields (4c–4i, 4o and 4p). 7-Substituted pyrrolo[1,2-*a*]quinoxaline also exhibited similar reactivity as reactants (4l–4m). In contrast, 6-substituted pyrrolo[1,2-*a*]quinoxaline substrates showed opposite reactivity (4j and 4k).

**Scheme 4:**
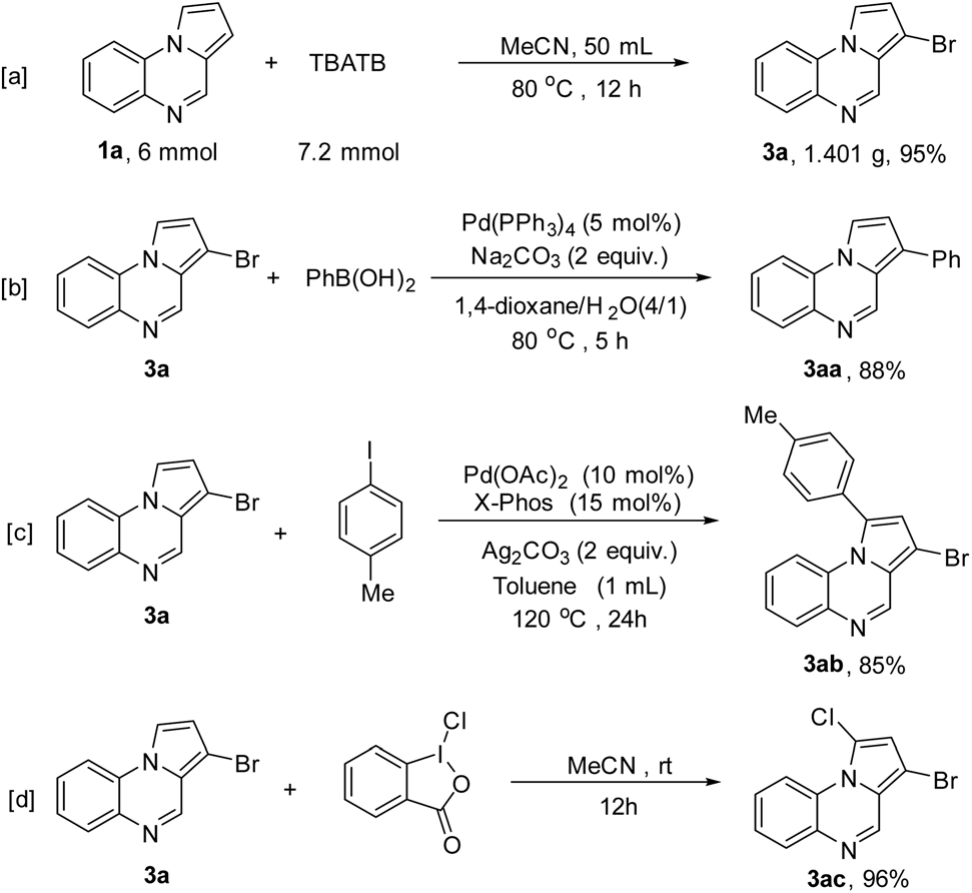
Gram-scale synthesis (a) and further transformations of 3a (b–d).

This C3-bromination can be easily scaled up using 6 mmol of 1a as reactant to give the gram-level products 3a in 95% yields (Scheme 4a). Next, the further transformations of 3a were investigated. The Suzuki coupling reaction of 3a with phenylboronic acid catalyzed by Pd(PPh_3_)_4_ readily afforded the 3-phenylpyrrolo[1,2-*a*]quinoxaline product 3aa in 88% yield (Scheme 4b). The reaction of 3a with 1-iodo-4-methylbenzene was executed by using Pd(OAc)_2_ as the catalyst and Ag_2_CO_3_ as the additive, and the C1-arylated product 3ab was obtained in 85% yield (Scheme 4c). In addition, the product 3a can be C1-chlorinated to give two different halogen-substituted product 3ac in excellent yield (Scheme 4d).

**Scheme 5:**
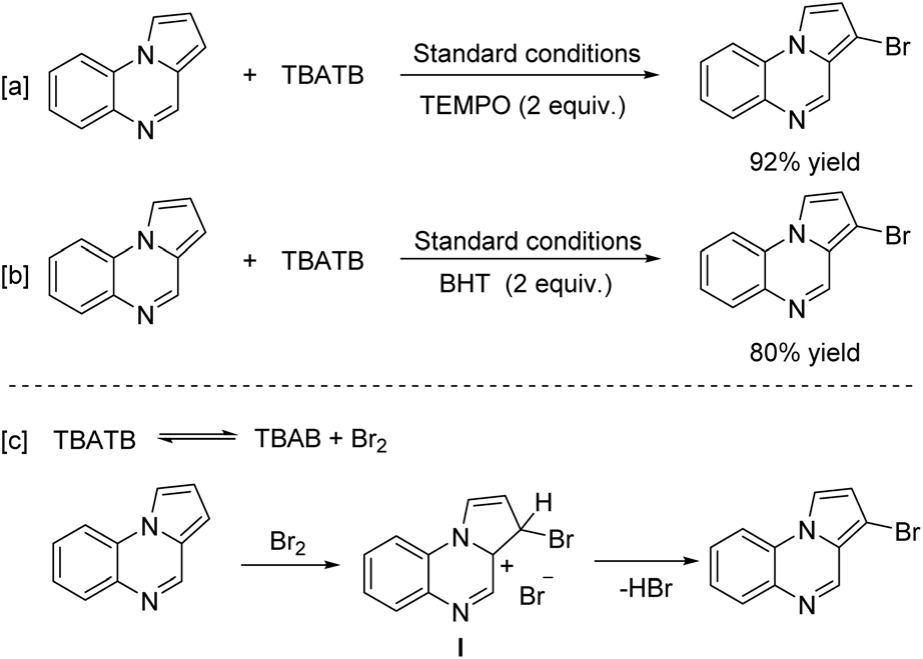
Control experiments (a and b) and proposed mechanism (c).

We next investigated the mechanistic information of this transformation by controlled experiments. The results showed that pyrrolo[1,2-*a*]quinoxaline (1a) could be brominated in the presence of free radical scavengers such as 2,2,6,6-tetramethylpiperidin-1-yloxy (TEMPO) and 2,6-di-*tert*-butyl-4-methylphenol (BHT) in yields of 92% and 80%, respectively (Scheme 5a and b). Therefore, we hypothesized that the method is not a radical process. We then proposed a possible reaction pathway (Scheme 5c). First, a slowly released molecule of bromine from TBATB undergoes electrophilic addition with 1a to generate the carbon-positive intermediate I. Finally, intermediate I strips off a molecule of HBr to give product 3a.

## Conclusions

In summary, this study presents a straightforward and efficient strategy for the regioselective bromination of pyrrolo[1,2-*a*]quinoxalines with TBATB. Various pyrrolo[1,2-*a*]quinoxalines can be efficiently introduced with one or two bromo functional groups at C3 or C1/C3, respectively, to provide scaffolds for their downstream decoration. This protocol is characterized by high selectivity, simple operation, environmental friendliness and easy scalability. It not only enriches the structural diversity of pyrrolo[1,2-*a*]quinoxaline derivatives, but also provides templates and inspirations for further studies on selective bromination of various other heterocycles using TBATB.

## Experimental

### Materials and instruments

Unless otherwise noted, all commercial materials were used directly without further purification, and all reactions were performed in the air. For chromatography, Qingdao Ocean Chemical 200–300 mesh silica gel was employed. Melting points were determined with a fusiometer. ^1^H NMR and ^13^C NMR spectra were recorded on Bruker Avance III HD 400 MHz and Bruker Ascend™ 600 MHZ spectrometer in CDCl_3_, and the chemical shifts are reported in ppm (*δ*) relative to the internal standard tetramethylsilane (TMS) (0 ppm). High-resolution mass spectra (HRMS) were acquired in atmospheric pressure chemical ionization (APCI) mode using a TOF mass analyzer.

### General procedure for the C3-bromination of pyrrolo[1,2-*a*]quinoxaline with TBATB

A 10 mL Schlenk tube was charged with pyrrolo[1,2-*a*]quinoxaline 1 (0.2 mmol), TBATB (0.24 mmol) and MeCN (2.5 mL). The mixture was stirred at 80 °C for 12 hours. After completion of the reaction, the solution was quenched with a saturated solution of sodium thiosulfate (10 mL) and extracted with ethyl acetate (15 mL × 3). The organic layer was dried with anhydrous Na_2_SO_4_ and the solvent was removed under reduced pressure, and the crude product was purified by rapid chromatography on silica gel (petroleum ether/ethyl acetate = 8 : 1) to give the final products 3a–3w.

### General procedure for the dibromination of pyrrolo[1,2-*a*]quinoxaline with TBATB

A 10 mL Schlenk tube was charged with pyrrolo[1,2-*a*]quinoxaline 1a (0.2 mmol), TBATB (0.24 mmol) and DMSO (2.5 mL). The mixture was stirred at 80 °C for 12 hours. After completion of the reaction, the solution was quenched with a saturated solution of sodium thiosulfate (10 mL) and extracted with ethyl acetate (15 mL × 3). The organic layer was dried with anhydrous Na_2_SO_4_ and the solvent was removed under reduced pressure. The residue was purified from the crude product by rapid chromatography on silica gel (petroleum ether/ethyl acetate = 10 : 1) to give the final products 5a–5f.

### Gram-scale synthesis procedure for 3a

To a 200 mL round-bottomed flask was added 1a (6 mmol), TBATB (7.2 mmol) and MeCN (50 mL). The solution was stirred at 80 °C for 12 hours. After completion of the reaction, the solution was quenched with a saturated solution of sodium thiosulfate (25 mL) and extracted with dichloromethane (30 mL × 3). The organic layer was dried with anhydrous Na_2_SO_4_ and the solvent was removed under reduced pressure. The residue was purified by rapid chromatography on silica gel (petroleum ether/ethyl acetate = 8 : 1) to afford the final product 3a (1.401 g, 95% yield).

### Palladium-catalyzed Suzuki–Miyaura reaction of 3a with phenylboronic acid

To a 10 mL Schlenk tube was added 3a (0.2 mmol) phenylboronic acid added to acid (0.4 mmol), Pd(PPh_3_)_4_ (5 mol%), Na_2_CO_3_ (2 equiv.) and 1,4-dioxane/H_2_O (4 : 1, 2.5 mL). The reaction vessel was stirred at 80 °C for 5 h under air atmosphere. Upon completion of the reaction, saturated aqueous NaCl solution (10 mL) was added to the reaction solution, followed by extraction with dichloromethane (15 mL × 3). The organic phase was dried with Na_2_SO_4_ and the solvent was removed under reduced pressure. The residue was purified by rapid chromatography on silica gel (petroleum ether/ethyl acetate = 5 : 1) to give the final product 3aa.

### Palladium-catalyzed C1-arylation of 3a with 4-iodotoluene

A 10 mL Schlenk tube was filled with 3a (0.25 mmol), 4-iodotoluene (0.5 mmol), Pd(OAc)_2_ (10 mol%), X-Phos (15 mol%) and toluene (1 mL). The mixture was stirred in air at 120 °C for 24 hours. The reaction solution was concentrated under reduced pressure and the residue was purified by silica gel column chromatography (petroleum ether/ethyl acetate = 30 : 1) to give the final product 3ab.

### C1-Chlorination of 3a

A 10 mL Schlenk tube was charged with 3a (0.2 mmol), 1-chloro-1,2-phenyliodono-3-one (0.24 mmol) and MeCN (2 mL). The mixture was then stirred at room temperature for 12 hours. After completion of the reaction, the solution was quenched with a saturated solution of sodium bicarbonate (10 mL) and extracted with dichloromethane (15 mL × 3). The organic layer was dried with anhydrous Na_2_SO_4_ and the solvent was removed under reduced pressure, and the residue was purified by rapid chromatography on silica gel (petroleum ether/ethyl acetate = 10 : 1) to give the final product 3ac.

### 3-Bromopyrrolo[1,2-*a*]quinoxaline [3a]

Mp. 166–171 °C. ^1^H NMR (400 MHz, CDCl_3_) *δ* 8.81 (s, 1H), 8.01 (dd, *J* = 8.0, 1.6 Hz, 1H), 7.88 (d, *J* = 2.8 Hz, 1H), 7.84 (dd, *J* = 8.0, 1.6 Hz, 1H), 7.56 (td, *J* = 7.2, 1.2 Hz, 1H), 7.49 (td, *J* = 8.0 1.2 Hz, 1H), 6.92 (d, *J* = 2.9 Hz, 1H). ^13^C NMR (101 MHz, CDCl_3_) *δ* 143.33, 137.03, 130.41, 128.85, 127.39, 126.50, 125.95, 120.86, 115.32, 99.35, 96.15. HRMS (APCI): *m*/*z* calcd for C_11_H_7_BrN_2_ [M + H]^+^: 246.9865, found: 246.9863.

### 3-Bromo-4-phenylpyrrolo[1,2-*a*]quinoxaline [3b]

Mp. 161–166 °C. ^1^H NMR (400 MHz, CDCl_3_) *δ* 8.03 (dd, *J* = 8.0, 1.2 Hz, 1H), 7.94 (d, *J* = 3.2 Hz, 1H), 7.83 (dd, *J* = 8.0, 1.2 Hz, 1H), 7.65–7.61 (m, 2H), 7.54 (dd, *J* = 7.2, 1.6 Hz, 1H), 7.51 (dd, *J* = 4.0, 2.4 Hz, 3H), 7.47 (td, *J* = 8.0, 1.2 Hz, 1H), 6.90 (d, *J* = 3.2 Hz, 1H). ^13^C NMR (101 MHz, CDCl_3_) *δ* 154.93, 137.49, 135.58, 130.36, 129.49, 129.37, 128.08, 128.05, 127.02, 125.87, 121.87, 118.21, 114.64, 113.14, 95.74. HRMS (APCI): *m*/*z* calcd for C_17_H_11_BrN_2_ [M + H]^+^: 323.0178, found: 323.0176.

### 3-Bromo-4-(*p*-tolyl)pyrrolo[1,2-*a*]quinoxaline [3c]

Mp. 174–179 °C. ^1^H NMR (400 MHz, CDCl_3_) *δ* 8.02 (dd, *J* = 8.0, 1.6 Hz, 1H), 7.94 (d, *J* = 2.8 Hz, 1H), 7.83 (dd, *J* = 8.0, 1.2 Hz, 1H), 7.56–7.50 (m, 3H), 7.46 (td, *J* = 7.6, 1.6 Hz, 1H), 7.31 (d, *J* = 8.0 Hz, 2H), 6.91 (d, *J* = 3.2 Hz, 1H), 2.46 (s, 3H). ^13^C NMR (101 MHz, CDCl_3_) *δ* 154.37, 139.44, 136.77, 134.42, 130.32, 129.37, 128.80, 128.35, 126.79, 126.36, 124.46, 123.00, 115.24, 99.78, 96.75, 21.65. HRMS (APCI): *m*/*z* calcd for C_18_H_13_BrN_2_ [M + H]^+^: 337.0335, found: 337.0330.

### 3-Bromo-4-(4-methoxyphenyl)pyrrolo[1,2-*a*]quinoxaline [3d]

Mp. 167–169 °C. ^1^H NMR (400 MHz, CDCl_3_) *δ* 8.00 (dd, *J* = 8.0, 1.6 Hz, 1H), 7.91 (d, *J* = 2.8 Hz, 1H), 7.80 (dd, *J* = 8.0, 1.6 Hz, 1H), 7.61 (d, *J* = 2.0 Hz, 1H), 7.59 (d, *J* = 2.0 Hz, 1H), 7.49 (td, *J* = 8.0, 1.6 Hz, 1H), 7.44 (td, *J* = 7.6, 1.2 Hz, 1H), 7.04 (d, *J* = 2.4 Hz, 1H), 7.02 (d, *J* = 2.0 Hz, 1H), 6.89 (d, *J* = 2.8 Hz, 1H), 3.89 (s, 3H). ^13^C NMR (101 MHz, CDCl_3_) *δ* 160.73, 154.54, 135.55, 131.08, 130.12, 129.87, 127.82, 126.85, 125.78, 121.94, 118.17, 114.64, 113.41, 113.07, 95.70, 55.45. HRMS (APCI): *m*/*z* calcd for C_18_H_13_BrN_2_O [M + H]^+^: 353.0284, found: 353.0280.

### 3-Bromo-4-(4-chlorophenyl)pyrrolo[1,2-*a*]quinoxaline [3e]

Mp. 199–201 °C. ^1^H NMR (400 MHz, CDCl_3_) *δ* 8.01 (dd, *J* = 8.0, 1.6 Hz, 1H), 7.96 (d, *J* = 2.8 Hz, 1H), 7.85 (dd, *J* = 8.0, 1.2 Hz, 1H), 7.60–7.57 (m, 2H), 7.54 (dd, *J* = 8.4, 1.6 Hz, 1H), 7.50–7.45 (m, 3H), 6.92 (d, *J* = 3.2 Hz, 1H). ^13^C NMR (101 MHz, CDCl_3_) *δ* 153.72, 135.92, 135.59, 135.51, 131.04, 130.40, 128.36, 128.33, 127.04, 126.03, 121.69, 118.33, 114.87, 113.22, 95.71. HRMS (APCI): *m*/*z* calcd for C_17_H_10_BrClN_2_ [M + H]^+^: 356.9789, found: 356.9762.

### 3-Bromo-4-(4-bromophenyl)pyrrolo[1,2-*a*]quinoxaline [3f]

Mp. 202–205 °C. ^1^H NMR (400 MHz, CDCl_3_) *δ* 8.01 (dd, *J* = 8.0, 1.6 Hz, 1H), 7.97 (d, *J* = 2.8 Hz, 1H), 7.85 (dd, *J* = 8.0, 1.2 Hz, 1H), 7.67–7.62 (m, 2H), 7.58–7.53 (m, 2H), 7.52–7.50 (m, 1H), 7.49–7.46 (m, 1H), 6.92 (d, *J* = 2.8 Hz, 1H). ^13^C NMR (101 MHz, CDCl_3_) *δ* 153.69, 136.26, 135.39, 131.30, 131.26, 130.32, 128.39, 127.00, 126.05, 123.91, 121.59, 118.36, 114.95, 113.23, 95.82. HRMS (APCI): *m*/*z* calcd for C_17_H_10_Br_2_N_2_ [M + H]^+^: 400.9284, found: 400.9261.

### 3-Bromo-4-(4-fluorophenyl)pyrrolo[1,2-*a*]quinoxaline [3g]

Mp. 174–176 °C. ^1^H NMR (400 MHz, CDCl_3_) *δ* 7.91 (dd, *J* = 8.0, 1.6 Hz, 1H), 7.85 (d, *J* = 2.8 Hz, 1H), 7.74 (dd, *J* = 8.0, 1.6 Hz, 1H), 7.57–7.49 (m, 2H), 7.44 (td, *J* = 8.0, 7.6, 1.6 Hz, 1H), 7.38 (td, *J* = 8.0, 7.6, 1.6 Hz, 1H), 7.14–7.06 (m, 2H), 6.82 (d, *J* = 2.8 Hz, 1H). ^13^C NMR (101 MHz, CDCl_3_) *δ* 153.81, 135.43, 133.47 (d, *J* = 3.3 Hz), 131.54 (d, *J* = 8.4 Hz), 130.26, 128.20, 126.95, 125.94, 121.75, 118.26, 115.08 (d, *J* = 21.9 Hz), 114.80, 113.15, 95.70. ^19^F NMR (376 MHz, CDCl_3_) *δ* −111.96. HRMS (APCI): *m*/*z* calcd for C_17_H_10_BrFN_2_ [M + H]^+^: 341.0084, found: 341.0083.

### 3-Bromo-4-(4-nitrophenyl)pyrrolo[1,2-*a*]quinoxaline [3h]

Mp. 244–249 °C. ^1^H NMR (400 MHz, CDCl_3_) *δ* 8.38 (d, *J* = 8.8 Hz, 2H), 8.07–8.00 (m, 2H), 7.90 (dd, *J* = 8.4, 0.8 Hz, 1H), 7.83 (d, *J* = 8.4 Hz, 2H), 7.61 (td, *J* = 8.4, 7.2, 1.2 Hz, 1H), 7.53 (td, *J* = 8.4, 7.2, 1.2 Hz, 1H), 6.97 (d, *J* = 2.8 Hz, 1H). ^13^C NMR (101 MHz, CDCl_3_) *δ* 152.57, 148.62, 143.70, 135.34, 130.86, 130.58, 128.98, 127.13, 126.32, 123.37, 121.35, 118.53, 115.27, 113.38, 95.67. HRMS (APCI): *m*/*z* calcd for C_17_H_10_BrN_3_O_2_ [M + H]^+^: 368.0029, found: 368.0027.

### 3-Bromo-4-(2,4-dichlorophenyl)pyrrolo[1,2-*a*]quinoxaline [3i]

Mp. 137–141 °C. ^1^H NMR (400 MHz, CDCl_3_) *δ* 8.03 (dd, *J* = 8.0, 1.6 Hz, 1H), 7.97 (d, *J* = 2.8 Hz, 1H), 7.87 (dd, *J* = 8.0, 1.2 Hz, 1H), 7.59 (td, *J* = 8.0, 1.2 Hz, 1H), 7.54 (t, *J* = 1.2 Hz, 1H), 7.50 (td, *J* = 8.0, 1.2 Hz, 2H), 7.41 (d, *J* = 1.6 Hz, 2H), 6.91 (d, *J* = 3.2 Hz, 1H); ^13^C NMR (101 MHz, CDCl_3_) *δ* 151.39, 136.02, 134.94, 131.64, 130.35, 129.47, 128.88, 127.37, 126.23, 121.88, 118.17, 115.17, 113.41. HRMS (APCI): *m*/*z* calcd for C_17_H_9_BrCl_2_N_2_ [M + H]^+^: 390.9398, found: 390.9395.

### 3-Bromo-1-phenylpyrrolo[1,2-*a*]quinoxaline [3j]

Mp. 153–158 °C. ^1^H NMR (400 MHz, CDCl_3_) *δ* 8.83 (s, 1H), 7.96 (dd, *J* = 8.0, 1.6 Hz, 1H), 7.51 (t, *J* = 3.2 Hz, 5H), 7.40–7.31 (m, 2H), 7.14 (td, *J* = 7.2, 1.6 Hz, 1H), 6.81 (s, 1H). ^13^C NMR (101 MHz, CDCl_3_) *δ* 144.35, 137.21, 132.99, 132.49, 130.33, 129.84, 129.36, 129.02, 128.52, 127.16, 125.67, 124.79, 118.76, 116.50, 95.96. HRMS (APCI): *m*/*z* calcd for C_17_H_11_BrN_2_ [M + H]^+^: 323.0178, found: 323.0177.

### 3-Bromo-1-(*p*-tolyl)pyrrolo[1,2-*a*]quinoxaline [3k]

Mp. 118–123 °C. ^1^H NMR (400 MHz, CDCl_*3*_) *δ* 8.81 (s, 1H), 7.95 (dd, *J* = 8.0, 1.6 Hz, 1H), 7.41–7.34 (m, 4H), 7.31 (d, *J* = 8.0 Hz, 2H), 7.14 (td, *J* = 8.0, 1.6 Hz, 1H), 6.77 (s, 1H), 2.48 (s, 3H). ^13^C NMR (101 MHz, CDCl_3_) *δ* 144.38, 139.40, 137.27, 132.62, 130.30, 130.03, 129.71, 128.63, 127.05, 125.58, 124.70, 118.63, 116.51, 95.82, 21.60. HRMS (APCI): *m*/*z* calcd for C_18_H_13_BrN_2_ [M + H]^+^: 337.0335, found: 337.0333.

### 3-Bromo-1-(4-methoxyphenyl)pyrrolo[1,2-*a*]quinoxaline [3l]

Mp. 148–150 °C. ^1^H NMR (400 MHz, CDCl_3_) *δ* 8.80 (s, 1H), 7.94 (dd, *J* = 8.0, 1.6 Hz, 1H), 7.42–7.33 (m, 4H), 7.17–7.11 (m, 1H), 7.03 (d, *J* = 8.4 Hz, 2H), 6.75 (s, 1H), 3.91 (s, 3H). ^13^C NMR (101 MHz, CDCl_3_) *δ* 160.42, 144.34, 137.26, 132.37, 131.17, 130.28, 128.67, 127.06, 125.54, 125.12, 124.58, 118.57, 116.38, 114.42, 95.74, 55.53. HRMS (APCI): *m*/*z* calcd for C_18_H_13_BrN_2_O [M + H]^+^: 353.0284, found: 353.0283.

### 3-Bromo-7-methylpyrrolo[1,2-*a*]quinoxaline [3m]

Mp. 174–179 °C. ^1^H NMR (400 MHz, CDCl_3_) *δ* 8.72 (s, 1H), 7.74 (dd, *J* = 7.2, 2.4 Hz, 2H), 7.64 (d, *J* = 8.4 Hz, 1H), 7.30 (dd, *J* = 8.4, 2.0 Hz, 1H), 6.83 (d, *J* = 2.8 Hz, 1H), 2.47 (s, 3H). ^13^C NMR (101 MHz, CDCl_3_) *δ* 143.86, 135.79, 135.71, 130.08, 129.42, 125.16, 123.73, 115.98, 113.89, 113.02, 94.65, 21.20. HRMS (APCI): *m*/*z* calcd for C_12_H_9_BrN_2_ [M + H]^+^: 261.0022, found: 261.0019.

### 3-Bromo-7-chloropyrrolo[1,2-*a*]quinoxaline [3n]

Mp. 167–172 °C. ^1^H NMR (400 MHz, CDCl_3_) *δ* 8.78 (s, 1H), 7.96 (d, *J* = 2.4 Hz, 1H), 7.82 (dd, *J* = 2.8, 0.8 Hz, 1H), 7.74 (d, *J* = 8.8 Hz, 1H), 7.49 (dd, *J* = 8.8, 2.4 Hz, 1H), 6.91 (d, *J* = 2.8 Hz, 1H). ^13^C NMR (101 MHz, CDCl_3_) *δ* 145.02, 136.68, 131.24, 129.73, 128.51, 126.05, 123.82, 116.86, 114.69, 114.61, 96.10. HRMS (APCI): *m*/*z* calcd for C_11_H_6_BrClN_2_ [M + H]^+^: 280.9476, found: 280.9473.

### 3-Bromo-4-(4-methoxyphenyl)-7-methylpyrrolo[1,2-*a*]quinoxaline [3o]

Mp. 162–167 °C. ^1^H NMR (400 MHz, CDCl_3_) *δ* 7.89 (d, *J* = 2.8 Hz, 1H), 7.82 (s, 1H), 7.71 (d, *J* = 8.4 Hz, 1H), 7.60 (d, *J* = 2.0 Hz, 1H), 7.58 (d, *J* = 2.0 Hz, 1H), 7.32 (dd, *J* = 8.4, 2.0 Hz, 1H), 7.03 (d, *J* = 2.0 Hz, 1H), 7.02 (d, *J* = 2.0 Hz, 1H), 6.88 (d, *J* = 3.2 Hz, 1H), 3.89 (s, 3H), 2.48 (s, 3H). ^13^C NMR (101 MHz, CDCl_3_) *δ* 160.79, 154.48, 135.80, 135.33, 131.16, 129.82, 129.08, 124.79, 121.86, 118.06, 114.60, 113.43, 112.86, 95.66, 55.49, 21.23. HRMS (APCI): *m*/*z* calcd for C_19_H_15_BrN_2_O [M + H]^+^: 367.0441, found: 367.0437.

### 3-Bromo-4-(4-bromophenyl)-7-methylpyrrolo[1,2-*a*]quinoxaline [3p]

Mp. 216–219 °C. ^1^H NMR (400 MHz, CDCl_3_) *δ* 7.68 (d, *J* = 3.2 Hz, 1H), 7.57 (s, 1H), 7.49 (d, *J* = 8.4 Hz, 1H), 7.39 (dd, *J* = 6.4, 2.0 Hz, 2H), 7.27 (dd, *J* = 6.4, 2.0 Hz, 2H), 7.12 (dd, *J* = 8.4, 2.0 Hz, 1H), 6.65 (d, *J* = 2.8 Hz, 1H), 2.25 (s, 3H). ^13^C NMR (101 MHz, CDCl_3_) *δ* 153.60, 136.40, 135.99, 135.36, 131.23, 130.09, 129.53, 124.90, 123.82, 121.52, 118.09, 114.71, 112.95, 95.41, 21.25. HRMS (APCI): *m*/*z* calcd for C_18_H_12_Br_2_N_2_ [M + H]^+^: 414.9440, found: 414.9436.

### 3-Bromo-8-methylpyrrolo[1,2-*a*]quinoxaline [3q]

Mp. 169–174 °C. ^1^H NMR (400 MHz, CDCl_3_) *δ* 8.72 (s, 1H), 7.84 (d, *J* = 8.4 Hz, 1H), 7.79 (d, *J* = 3.2 Hz, 1H), 7.58 (d, *J* = 2.0 Hz, 1H), 7.26 (dd, *J* = 8.0, 2.0 Hz, 1H), 6.86 (d, *J* = 2.8 Hz, 1H), 2.53 (s, 3H). ^13^C NMR (101 MHz, CDCl_3_) *δ* 143.03, 139.08, 133.78, 130.02, 127.22, 127.13, 123.92, 116.23, 113.86, 113.42, 94.69, 21.96. HRMS (APCI): *m*/*z* calcd for C_12_H_9_BrN_2_ [M + H]^+^: 261.0022, found: 261.0020.

### 3-Bromo-8-chloropyrrolo[1,2-*a*]quinoxaline [3r]

Mp. 169–174 °C. ^1^H NMR (400 MHz, CDCl_3_) *δ* 8.82 (s, 1H), 8.04 (d, *J* = 8.8 Hz, 1H), 7.88 (d, *J* = 2.8 Hz, 1H), 7.85 (d, *J* = 2.0 Hz, 1H), 7.47 (dd, *J* = 8.4, 2.0 Hz, 1H), 7.00 (d, *J* = 2.8 Hz, 1H). ^13^C NMR (101 MHz, CDCl_3_) *δ* 143.51, 134.45, 130.95, 127.97, 127.01, 126.74, 123.73, 117.50, 115.46, 115.36, 113.81. HRMS (APCI): *m*/*z* calcd for C_11_H_6_BrClN_2_ [M + H]^+^: 280.9476, found: 280.9474.

### 3-Bromo-8-fluoropyrrolo[1,2-*a*]quinoxaline [3s]

Mp. 164–166 °C. ^1^H NMR (400 MHz, CDCl_3_) *δ* 8.78 (s, 1H), 8.02 (dd, *J* = 8.8, 5.6 Hz, 1H), 7.78 (d, *J* = 3.2 Hz, 1H), 7.51 (dd, *J* = 8.8, 2.4 Hz, 1H), 7.22 (td, *J* = 9.2, 2.8 Hz, 1H), 6.95 (d, *J* = 2.8 Hz, 1H). ^13^C NMR (101 MHz, CDCl_3_) *δ* 161.94 (d, *J* = 250.1 Hz), 143.25, 132.33 (d, *J* = 9.6 Hz), 116.98, 114.50, 114.10, 100.55, 100.37. ^19^F NMR (376 MHz, CDCl_3_) *δ* −109.16. HRMS (APCI): *m*/*z* calcd for C_11_H_6_BrFN_2_ [M + H]^+^: 264.9771, found: 264.9769.

### 1-Bromo-3-phenylpyrrolo[1,2-*a*]quinoxaline [3t]

Mp. 137–141 °C. ^1^H NMR (400 MHz, CDCl_3_) *δ* 9.27 (dd, *J* = 8.0, 2.0 Hz, 1H), 8.90 (s, 1H), 7.98 (dd, *J* = 7.2, 2.4 Hz, 1H), 7.57 (d, *J* = 7.2 Hz, 2H), 7.53–7.46 (m, 4H), 7.40 (t, *J* = 7.2 Hz, 1H), 7.03 (s, 1H). ^13^C NMR (101 MHz, CDCl_3_) *δ* 144.08, 137.05, 133.00, 129.85, 129.23, 128.72, 127.86, 127.09, 126.11, 124.81, 124.70, 119.02, 115.61, 100.11. HRMS (APCI): *m*/*z* calcd for C_17_H_11_BrN_2_ [M + H]^+^: 323.0178, found: 323.0177.

### 1-Bromo-3-(*p*-tolyl)pyrrolo[1,2-*a*]quinoxaline [3u]

Mp. 150–155 °C. ^1^H NMR (400 MHz, CDCl_3_) *δ* 9.26 (dd, *J* = 8.0, 2.0 Hz, 1H), 8.88 (s, 1H), 7.98 (dd, *J* = 7.2, 2.0 Hz, 1H), 7.54–7.45 (m, 4H), 7.30 (d, *J* = 7.6 Hz, 2H), 7.01 (s, 1H), 2.43 (s, 3H). ^13^C NMR (101 MHz, CDCl_3_) *δ* 144.28, 137.76, 137.20, 130.10, 129.94, 129.90, 129.35, 128.58, 126.99, 126.03, 124.76, 124.67, 118.89, 115.59, 99.91, 21.37. HRMS (APCI): *m*/*z* calcd for C_18_H_13_BrN_2_ [M + H]^+^: 337.0335, found: 337.0333.

### 4-(1-Bromopyrrolo[1,2-*a*]quinoxalin-3-yl)benzonitrile [3v]

Mp. 240–242 °C. ^1^H NMR (400 MHz, CDCl_3_) *δ* 9.32 (dd, *J* = 8.0, 2.0 Hz, 1H), 8.90 (s, 1H), 8.04 (dd, *J* = 7.2, 2.0 Hz, 1H), 7.79 (d, *J* = 8.4 Hz, 2H), 7.68 (d, *J* = 8.4 Hz, 2H), 7.57 (pd, *J* = 7.2, 2.0 Hz, 2H), 7.08 (s, 1H). ^13^C NMR (101 MHz, CDCl_3_) *δ* 143.08, 137.69, 133.06, 129.98, 129.10, 127.72, 126.66, 119.12, 118.81, 115.77, 111.40, 101.17. HRMS (APCI): *m*/*z* calcd for C_18_H_10_BrN_3_ [M + H]^+^: 348.0131, found: 348.0129.

### 1,3-Dibromopyrrolo[1,2-*a*]quinoxaline [4a]

Mp. 157–162 °C. ^1^H NMR (400 MHz, CDCl_3_) *δ* 9.18–9.23 (m, 1H), 8.71 (s, 1H), 8.03–7.96 (m, 1H), 7.51–7.53 (m, 2H), 6.91 (s, 1H). ^13^C NMR (101 MHz, CDCl_3_) *δ* 143.32, 137.00, 130.40, 128.87, 127.42, 126.53, 125.97, 120.90, 115.34, 99.42, 96.23. HRMS (APCI): *m*/*z* calcd for C_11_H_6_Br_2_N_2_ [M + H]^+^: 324.8971, found: 324.8968.

### 1,3-Dibromo-4-phenylpyrrolo[1,2-*a*]quinoxaline [4b]

Mp. 151–153 °C. ^1^H NMR (400 MHz, CDCl_3_) *δ* 9.21–9.13 (m, 1H), 7.96–7.90 (m, 1H), 7.51–7.45 (m, 2H), 7.44–7.38 (m, 5H), 6.84 (s, 1H). ^13^C NMR (101 MHz, CDCl_3_) *δ* 154.29, 137.32, 136.77, 130.41, 129.43, 129.41, 128.43, 128.18, 126.94, 126.42, 124.38, 123.03, 115.29, 99.87, 96.74. HRMS (APCI): *m*/*z* calcd for C_17_H_10_Br_2_N_2_ [M + H]^+^: 400.9284, found: 400.9280.

### 1,3-Dibromo-4-(*p*-tolyl)pyrrolo[1,2-*a*]quinoxaline [4c]

Mp. 159–162 °C. ^1^H NMR (400 MHz, CDCl_3_) *δ* 9.29–9.21 (m, 1H), 8.05–7.99 (m, 1H), 7.53–7.47 (m, 3H), 7.46 (s, 1H), 7.31 (s, 1H), 7.29 (s, 1H), 6.93 (s, 1H), 2.45 (s, 3H). ^13^C NMR (101 MHz, CDCl_3_) *δ* 155.01, 139.42, 135.52, 134.50, 130.24, 129.48, 128.72, 127.98, 126.97, 125.86, 121.94, 118.25, 114.69, 113.13, 95.88, 21.66. HRMS (APCI): *m*/*z* calcd for C_18_H_12_Br_2_N_2_ [M + H]^+^: 414.9440, found: 414.9435.

### 1,3-Dibromo-4-(4-methoxyphenyl)pyrrolo[1,2-*a*]quinoxaline [4d]

Mp. 159–162 °C. ^1^H NMR (400 MHz, CDCl_3_) *δ* 9.22–9.15 (m, 1H), 7.95–7.97 (m, 1H), 7.46 (d, *J* = 2.0 Hz, 1H), 7.45–7.40 (m, 3H), 6.95 (d, *J* = 2.0 Hz, 1H), 6.93 (d, *J* = 2.0 Hz, 1H), 6.87 (s, 1H), 3.81 (s, 3H). ^13^C NMR (101 MHz, CDCl_3_) *δ* 160.89, 154.01, 136.68, 131.05, 130.17, 128.33, 126.82, 126.45, 124.54, 123.14, 115.31, 113.60, 100.01, 97.18, 55.52. HRMS (APCI): *m*/*z* calcd for C_18_H_12_Br_2_N_2_O [M + H]^+^: 430.9389, found: 430.9385.

### 1,3-Dibromo-4-(4-chlorophenyl)pyrrolo[1,2-*a*]quinoxaline [4e]

Mp. 189–193 °C. ^1^H NMR (400 MHz, CDCl_3_) *δ* 9.31–9.22 (m, 1H), 8.04–7.97 (m, 1H), 7.51–7.55 (m, 4H), 7.46–7.49 (m, 2H), 6.94 (s, 1H). ^13^C NMR (101 MHz, CDCl_3_) *δ* 153.07, 136.59, 135.71, 130.96, 130.36, 128.45, 128.43, 127.22, 126.58, 124.16, 123.15, 115.36, 100.26, 96.80. HRMS (APCI): *m*/*z* calcd for C_17_H_9_Br_2_ClN_2_ [M + H]^+^: 434.8894, found: 434.8890.

### 1,3-Dibromo-4-(4-bromophenyl)pyrrolo[1,2-*a*]quinoxaline [4f]

Mp. 207–211 °C. ^1^H NMR (400 MHz, CDCl_3_) *δ* 9.32–9.23 (m, 1H), 8.06–7.96 (m, 1H), 7.63 (d, *J* = 8.0 Hz, 2H), 7.58–7.49 (m, 2H), 7.46 (d, *J* = 8.0 Hz, 2H), 6.95 (s, 1H). ^13^C NMR (101 MHz, CDCl_3_) *δ* 153.06, 136.53, 136.06, 131.39, 131.21, 130.33, 128.41, 127.25, 126.60, 124.08, 124.00, 123.17, 115.37, 100.33, 96.87. HRMS (APCI): *m*/*z* calcd for C_17_H_9_Br_3_N_2_ [M + H]^+^: 478.8389, found: 478.8386.

### 1,3-Dibromo-4-(4-fluorophenyl)pyrrolo[1,2-*a*]quinoxaline [4g]

Mp. 168–169 °C. ^1^H NMR (400 MHz, CDCl_3_) *δ* 9.31–9.23 (m, 1H), 8.06–8.00 (m, 1H), 7.60–7.48 (m, 4H), 7.19 (t, *J* = 8.8 Hz, 2H), 6.95 (s, 1H). ^13^C NMR (101 MHz, CDCl_3_) *δ* 165.04, 162.57, 153.24, 131.56, 131.47, 130.29, 128.44, 127.18, 126.59, 124.30, 123.20, 115.41, 115.37, 115.19, 100,40, 97.24. ^19^F NMR (376 MHz, CDCl_3_) *δ* −111.77 (s). HRMS (APCI): *m*/*z* calcd for C_17_H_9_Br_2_FN_2_ [M + H]^+^: 418.9189, found: 418.9185.

### 1,3-Dibromo-4-(4-nitrophenyl)pyrrolo[1,2-*a*]quinoxaline [4h]

Mp. 159–162 °C. ^1^H NMR (400 MHz, CDCl_3_) *δ* 9.31 (dd, *J* = 8.0, 2.0 Hz, 1H), 8.37 (d, *J* = 8.4 Hz, 2H), 8.03 (dd, *J* = 7.6, 2.0 Hz, 1H), 7.77 (d, *J* = 8.0 Hz, 2H), 7.57 (pd, *J* = 7.2, 1.6 Hz, 2H), 6.99 (s, 1H). ^13^C NMR (101 MHz, CDCl_3_) *δ* 151.92, 148.68, 143.48, 136.44, 130.81, 130.54, 128.54, 127.81, 126.85, 123.78, 123.46, 123.30, 115.51, 100.83, 96.72. HRMS (APCI): *m*/*z* calcd for C_17_H_9_Br_2_N_3_O_2_ [M + H]^+^: 445.9134, found: 445.9131.

### 1,3-Dibromo-4-(2,4-dichlorophenyl)pyrrolo[1,2-*a*]quinoxaline [4i]

Mp. 145–150 °C. ^1^H NMR (400 MHz, CDCl_3_) *δ* 9.27 (dd, *J* = 8.0, 1.6 Hz, 1H), 8.01 (dd, *J* = 7.6, 2.0 Hz, 1H), 7.56–7.50 (m, 3H), 7.41 (dd, *J* = 8.0, 1.6 Hz, 1H), 7.38 (d, *J* = 8.4 Hz, 1H), 6.92 (s, 1H). ^13^C NMR (101 MHz, CDCl_3_) *δ* 150.72, 136.48, 136.00, 134.96, 131.62, 130.53, 130.17, 129.42, 128.76, 127.56, 127.38, 126.58, 124.15, 122.79, 115.36, 100.16, 96.64. HRMS (APCI): *m*/*z* calcd for C_17_H_8_Br_2_Cl_2_N_2_ [M + H]^+^: 468.8504, found: 468.8500.

### 1,3-Dibromo-7-methylpyrrolo[1,2-*a*]quinoxaline [4j]

Mp. 149–151 °C. ^1^H NMR (400 MHz, CDCl_3_) *δ* 8.99 (d, *J* = 8.8 Hz, 1H), 8.63 (s, 1H), 7.74–7.70 (m, 1H), 7.28 (dd, *J* = 8.8, 2.0 Hz, 1H), 6.84 (s, 1H), 2.47 (s, 3H). ^13^C NMR (101 MHz, CDCl_3_) *δ* 143.18, 136.95, 136.42, 130.14, 128.38, 126.58, 125.79, 120.43, 114.94, 98.99, 95.75, 77.48, 77.16, 76.84, 21.06. HRMS (APCI): *m*/*z* calcd for C_12_H_8_Br_2_N_2_ [M + H]^+^: 338.9127, found: 338.9124.

### 1,3-Dibromo-7-chloropyrrolo[1,2-*a*]quinoxaline [4k]

Mp. 187–190 °C. ^1^H NMR (600 MHz, CDCl_3_) *δ* 9.22 (d, *J* = 2.2 Hz, 1H), 9.22 (d, *J* = 2.4 Hz, 1H), 8.69 (s, 1H), 7.92 (d, *J* = 8.4 Hz, 1H), 7.47 (dd, *J* = 9.0, 2.4 Hz, 1H), 6.95 (s, 1H). ^13^C NMR (101 MHz, CDCl_3_) *δ* 143.53, 135.79, 132.81, 131.48, 129.24, 126.93, 125.93, 121.33, 115.42, 99.76, 96.69. HRMS (APCI): *m*/*z* calcd for C_11_H_5_Br_2_ClN_2_ [M + H]^+^: 358.8581, found: 358.8587.

### 1,3-Dibromo-8-methylpyrrolo[1,2-*a*]quinoxaline [4l]

Mp. 166–171 °C. ^1^H NMR (400 MHz, CDCl_3_) *δ* 8.64 (s, 1H), 8.64 (s, 1H), 7.86 (d, *J* = 8.4 Hz, 1H), 7.31 (dd, *J* = 8.4, 1.6 Hz, 1H), 6.88 (s, 1H), 2.53 (s, 3H). ^13^C NMR (101 MHz, CDCl_3_) *δ* 142.25, 138.01, 134.75, 129.90, 128.62, 127.71, 125.97, 120.85, 115.33, 99.09, 95.95, 22.28. HRMS (APCI): *m*/*z* calcd for C_12_H_8_Br_2_N_2_ [M + H]^+^: 338.9127, found: 338.9125.

### 1,3-Dibromo-8-chloropyrrolo[1,2-*a*]quinoxaline [4m]

Mp. 197–199 °C. ^1^H NMR (600 MHz, CDCl_3_) *δ* 9.15 (d, *J* = 9.0 Hz, 1H), 8.72 (s, 1H), 7.99 (d, *J* = 2.4 Hz, 1H), 7.49 (dd, *J* = 9.6, 2.4 Hz, 1H), 6.95 (s, 1H). ^13^C NMR (101 MHz, CDCl_3_) *δ* 144.50, 138.36, 131.79, 129.79, 127.43, 127.31, 125.91, 121.12, 116.41, 99.77, 96.78. HRMS (APCI): *m*/*z* calcd for C_11_H_5_Br_2_ClN_2_ [M + H]^+^: 358.8581, found: 358.8586.

### 1,3-Dibromo-8-fluoropyrrolo[1,2-*a*]quinoxaline [4n]

Mp. 184–186 °C. ^1^H NMR (400 MHz, CDCl_3_) *δ* 8.93 (dd, *J* = 10.8, 2.4 Hz, 1H), 8.66 (s, 1H), 7.97 (dd, *J* = 8.8, 6.0 Hz, 1H), 7.27–7.21 (m, 1H), 6.93 (s, 1H). ^13^C NMR (101 MHz, CDCl_3_) *δ* 160.53 (d, *J* = 248.6 Hz), 142.47, 133.51, 131.86 (d, *J* = 9.8 Hz), 125.59, 121.30, 114.48, 114.33, 102.57 (d, *J* = 29.8 Hz), 99.59, 96.57. ^19^F NMR (376 MHz, CDCl_3_) *δ* −108.43. HRMS (APCI): *m*/*z* calcd for C_11_H_5_Br_2_FN_2_ [M + H]^+^: 342.8876, found: 342.8872.

### 1,3-Dibromo-4-(4-methoxyphenyl)-7-methylpyrrolo[1,2-*a*]quinoxaline [4o]

Mp. 166–168 °C. ^1^H NMR (400 MHz, CDCl_3_) *δ* 7.89 (d, *J* = 2.8 Hz, 1H), 7.82 (s, 1H), 7.71 (d, *J* = 8.4 Hz, 1H), 7.60 (d, *J* = 2.0 Hz, 1H), 7.58 (d, *J* = 2.0 Hz, 1H), 7.32 (dd, *J* = 8.4, 2.0 Hz, 1H), 7.03 (d, *J* = 2.0 Hz, 1H), 7.02 (d, *J* = 1.8 Hz, 1H), 6.88 (d, *J* = 3.2 Hz, 1H), 3.89 (s, 3H), 2.48 (s, 3H). ^13^C NMR (101 MHz, CDCl_3_) *δ* 160.79, 153.92, 136.67, 136.36, 131.02, 129.97, 127.88, 126.15, 124.40, 122.73, 115.00, 113.54, 99.66, 96.52, 55.50, 21.05. HRMS (APCI): *m*/*z* calcd for C_19_H_14_Br_2_N_2_O [M + H]^+^: 444.9546, found: 444.9542.

### 1,3-Dibromo-4-(4-bromophenyl)-7-methylpyrrolo[1,2-*a*]quinoxaline [4q]

Mp. 124–129 °C. ^1^H NMR (400 MHz, CDCl_3_) *δ* 9.14 (d, *J* = 8.8 Hz, 1H), 7.83 (s, 1H), 7.64 (d, *J* = 2.0 Hz, 1H), 7.62 (d, *J* = 2.0 Hz, 1H), 7.46 (d, *J* = 2.0 Hz, 1H), 7.44 (d, *J* = 2.0 Hz, 1H), 7.35 (dd, *J* = 8.8, 1.6 Hz, 1H), 6.93 (s, 1H), 2.49 (s, 3H). ^13^C NMR (101 MHz, CDCl_3_) *δ* 153.04, 136.59, 136.33, 131.36, 131.22, 130.21, 128.35, 126.29, 124.01, 123.89, 122.80, 115.11, 99.98, 96.44, 21.06. HRMS (APCI): *m*/*z* calcd for C_18_H_11_Br_3_N_2_ [M + H]^+^: 492.8545, found: 492.8542.

### 3-Phenylpyrrolo[1,2-*a*]quinoxaline [3aa]

Mp. 178–180 °C. ^1^H NMR (400 MHz, CDCl_3_) *δ* 9.04 (s, 1H), 7.97 (dd, *J* = 8.0, 1.6 Hz, 1H), 7.93 (d, *J* = 2.8 Hz, 1H), 7.84 (dd, *J* = 8.0, 1.2 Hz, 1H), 7.65 (d, *J* = 7.2 Hz, 2H), 7.55–7.42 (m, 4H), 7.37 (t, *J* = 7.2 Hz, 1H), 7.02 (d, *J* = 2.8 Hz, 1H). ^13^C NMR (101 MHz, CDCl_3_) *δ* 145.16, 136.15, 134.49, 130.08, 129.15, 128.36, 128.08, 128.03, 127.19, 125.49, 123.88, 122.75, 114.28, 113.84, 113.80. HRMS (APCI): *m*/*z* calcd for C_17_H_12_N_2_ [M + H]^+^: 245.1073, found: 245.1071.

### 3-Bromo-1-(*p*-tolyl)pyrrolo[1,2-*a*]quinoxaline [3ab]

Mp. 118–123 °C. ^1^H NMR (400 MHz, CDCl_3_) *δ* 8.82 (s, 1H), 7.98 (d, *J* = 8.4 Hz, 1H), 7.35–7.41 (m, 4H), 7.32 (d, *J* = 8.0 Hz, 2H), 7.18–7.13 (m, 1H), 6.79 (s, 1H), 2.49 (s, 3H). ^13^C NMR (101 MHz, CDCl_3_) *δ* 144.00, 139.55, 136.70, 133.10, 129.95, 129.88, 129.76, 129.69, 128.59, 127.22, 125.74, 124.65, 118.93, 116.57, 96.56, 21.61. HRMS (APCI): *m*/*z* calcd for C_18_H_13_BrN_2_ [M + H]^+^: 337.0335, found: 337.0331.

### 3-Bromo-1-chloropyrrolo[1,2-*a*]quinoxaline [3ac]

Mp 136–138 °C. ^1^H NMR (400 MHz, CDCl_3_) *δ* 8.93 (dd, *J* = 6.4, 3.6 Hz, 1H), 8.68 (s, 1H), 7.95 (dd, *J* = 6.4, 3.2 Hz, 1H), 7.48 (dd, *J* = 6.4, 3.2 Hz, 2H), 6.78 (s, 1H). ^13^C NMR (101 MHz, CDCl_3_) *δ* 143.51, 137.12, 130.42, 128.48, 127.57, 126.41, 124.31, 116.69, 115.76, 115.67, 95.02. HRMS (APCI): *m*/*z* calcd for C_11_H_6_BrClN_2_ [M + H]^+^: 280.9476, found: 280.9473.

## Data availability

The data supporting this article have been included as part of the ESI.†

## Conflicts of interest

There are no conflicts to declare.

## Supplementary Material

RA-014-D4RA07358D-s001

RA-014-D4RA07358D-s002
