# Supplementary material for: Regioselective bromination of pyrrolo[1,2-a]quinoxalines
Source: RSC Adv. 2024 Nov 15;14(49):36488–96. doi: 10.1039/d4ra07358d (PMC11565163; doi:10.1039/d4ra07358d)

Supplementary Information (SI) for RSC Advances.
This journal is © The Royal Society of Chemistry 2024

Regioselective Bromination of Pyrrolo[1,2-a]quinoxalines

Yingqian Li, Yali Liu, Di Hao, Liang Xu* and Ping Liu*

School of Chemistry and Chemical Engineering/Key Laboratory for Green Processing of Chemical Engineering of Xinjiang Bingtuan, Shihezi University, Shihezi 832004, China.

Supporting Information

1. Materials and instruments 2

2. Experimental Section 2

3. The NMR Spectra Data 3

4. Copies of Spectra 13

1. Materials and instruments

Unless otherwise noted, all commercial materials were used directly without further purification, and all reactions were performed in the air. For chromatography, Qingdao Ocean Chemical 200-300 mesh silica gel was employed. Melting points were determined with a fusiometer. ^{1}H NMR and ^{13}C NMR spectra were recorded on Bruker Avance III HD 400 MHz and Bruker AscendTM 600 MHZ spectrometer in $CDCl_3$ and the chemical shifts are reported in ppm (δ) relative to the internal standard tetramethylsilane (TMS) (0 ppm). High-resolution mass spectra (HRMS) were acquired in atmospheric pressure chemical ionization (APCI) mode using a TOF mass analyzer.

2. Experimental Section

General procedure for the C3-bromination of pyrrolo[1,2-*a*]quinoxaline with TBATB

A 10 mL Schlenk tube was charged with pyrrolo[1,2-*a*]quinoxaline 1 (0.2 mmol), TBATB (0.24 mmol) and MeCN (2.5 mL). The mixture was stirred at 80 °C for 12 hours. After completion of the reaction, the solution was quenched with a saturated solution of sodium thiosulfate (10 mL) and extracted with ethyl acetate (15 mL × 3). The organic layer was dried with anhydrous Na_2SO_4 and the solvent was removed under reduced pressure, and the crude product was purified by rapid chromatography on silica gel (petroleum ether/ethyl acetate = 8:1) to give the final products **3a-3w**.

General procedure for the dibromination of pyrrolo[1,2-*a*]quinoxaline with TBATB

A 10 mL Schlenk tube was charged with pyrrolo[1,2-*a*]quinoxaline 1 (0.2 mmol), TBATB (0.24 mmol) and DMSO (2.5 mL). The mixture was stirred at 80°C for 12 hours. After completion of the reaction, the solution was quenched with a saturated solution of sodium thiosulfate (10 mL) and extracted with ethyl acetate (15 mL × 3). The organic layer was dried with anhydrous Na_2SO_4 and the solvent was removed under reduced pressure. The residue was purified from the crude product by rapid chromatography on silica gel (petroleum ether/ethyl acetate = 10:1) to give the final

products **5a-5f**.

Gram-scale synthesis procedure for 3a

To a 200 mL round-bottomed flask was added 1a (6 mmol), TBATB (7.2 mmol) and MeCN (50 mL). The solution was stirred at 100 °C for 12 hours. After completion of the reaction, the solution was quenched with a saturated solution of sodium thiosulfate (25 mL) and extracted with dichloromethane (30 mL × 3). The organic layer was dried with anhydrous Na_2SO_4 and the solvent was removed under reduced pressure. The residue was purified by rapid chromatography on silica gel (petroleum ether/ethyl acetate = 8:1) to afford the final product **3a** (1.401 g, 95% yield).

Palladium-catalyzed Suzuki-Miyaura reaction of 3a with phenylboronic acid

To a 10 mL Schlenk tube was added **3a** (0.2 mmol) phenylboronic acid added to acid (0.4 mmol), $Pd(PPh_3)_4$ (5 mol%), Na_2CO_3 (2 equiv.) and 1,4-dioxane/H_2O (4:1, 2.5 mL). The reaction vessel was stirred at 80 °C for 5 h under air atmosphere. Upon completion of the reaction, saturated aqueous NaCl solution (10 mL) was added to the reaction solution, followed by extraction with dichloromethane (15 mL × 3). The organic phase was dried with Na_2SO_4 and the solvent was removed under reduced pressure. The residue was purified by rapid chromatography on silica gel (petroleum ether/ethyl acetate = 5:1) to give the final product **3aa**.

Palladium-catalyzed C1-arylation of 3a with 4-iodotoluene

A 10 mL Schlenk tube was filled with **3a** (0.25 mmol), 4-iodotoluene (0.5 mmol), $Pd(OAc)_2$ (10 mol%), X-Phos (15 mol%) and toluene (1 mL). The mixture was stirred in air at 120 °C for 24 hours. The reaction solution was concentrated under reduced pressure and the residue was purified by silica gel column chromatography (petroleum ether/ethyl acetate = 30:1) to give the final product **3ab**.

C1-Chlorination of 3a

A 10 mL Schlenk tube was charged with 3a (0.2 mmol), 1-chloro-1,2-phenyliodono-3-one (0.24 mmol) and MeCN (2 mL). The mixture was then stirred at room temperature for 12 hours. After completion of the reaction, the solution was quenched with a saturated solution of sodium bicarbonate (10 mL) and extracted with dichloromethane (15 mL × 3). The organic layer was dried with

anhydrous Na_2SO_4 and the solvent was removed under reduced pressure, and the residue was purified by rapid chromatography on silica gel (petroleum ether/ethyl acetate = 10:1) to give the final product **3ac**.

3. The NMR Spectra Data

3-bromopyrrolo[1,2-*a*]quinoxaline [3a]

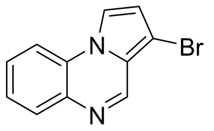

Mp. 166-171 °C. White solid (46.4 mg, 94%).

^{1}H NMR (400 MHz, $CDCl_3$) δ 8.81 (s, 1H), 8.01 (dd, J = 8.0, 1.6 Hz, 1H), 7.88 (d, J = 2.8 Hz, 1H), 7.84 (dd, J = 8.0, 1.6 Hz, 1H), 7.56 (td, J = 7.2, 1.2 Hz, 1H), 7.49 (td, J = 8.0 1.2 Hz, 1H), 6.92 (d, J = 2.9 Hz, 1H).

^{13}C NMR (101 MHz, $CDCl_3$) δ 143.33, 137.03, 130.41, 128.85, 127.39, 126.50, 125.95, 120.86, 115.32, 99.35, 96.15.

HRMS (APCI): m/z calcd for $C_{11}H_7BrN_2$ $[M+H]^+$: 246.9865, found: 246.9863.

3-bromo-4-phenylpyrrolo[1,2-*a*]quinoxaline [3b]

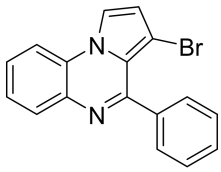

Mp. 161-166 °C. Light yellow solid (48.6 mg, 76%).

^{1}H NMR (400 MHz, $CDCl_3$) δ 8.03 (dd, J = 8.0, 1.2 Hz, 1H), 7.94 (d, J = 3.2 Hz, 1H), 7.83 (dd, J = 8.0, 1.2 Hz, 1H), 7.65-7.61 (m, 2H), 7.54 (dd, J = 7.2, 1.6 Hz, 1H), 7.51 (dd, J = 4.0, 2.4 Hz, 3H), 7.47 (td, J = 8.0, 1.2 Hz, 1H), 6.90 (d, J = 3.2 Hz, 1H).

^{13}C NMR (101 MHz, $CDCl_3$) δ 154.93, 137.49, 135.58, 130.36, 129.49, 129.37, 128.08, 128.05, 127.02, 125.87, 121.87, 118.21, 114.64, 113.14, 95.74.

HRMS (APCI): m/z calcd for $C_{17}H_{11}BrN_2$ $[M+H]^+$: 323.0178, found: 323.0176.

3-bromo-4-(p-tolyl)pyrrolo[1,2-*a*]quinoxaline [3c]

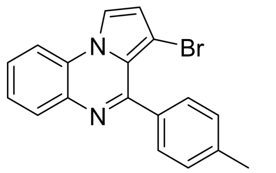

Mp. 174-179 °C. Light yellow solid (24.2 mg, 36%).

^{1}H NMR (400 MHz, $CDCl_3$) δ 8.02 (dd, J = 8.0, 1.6 Hz, 1H), 7.94 (d, J = 2.8 Hz, 1H), 7.83 (dd, J = 8.0, 1.2 Hz, 1H), 7.56-7.50 (m, 3H), 7.46 (td, J = 7.6, 1.6 Hz, 1H), 7.31 (d, J = 8.0 Hz, 2H), 6.91 (d, J = 3.2 Hz, 1H), 2.46 (s, 3H).

^{13}C NMR (101 MHz, $CDCl_3$) δ 154.37, 139.44, 136.77, 134.42, 130.32, 129.37, 128.80, 128.35, 126.79, 126.36, 124.46, 123.00, 115.24, 99.78, 96.75, 21.65.

HRMS (APCI): m/z calcd for $C_{18}H_{13}BrN_2$ $[M+H]^+$: 337.0335, found: 337.0330.

3-bromo-4-(4-methoxyphenyl)pyrrolo[1,2-*a*]quinoxaline [3d]

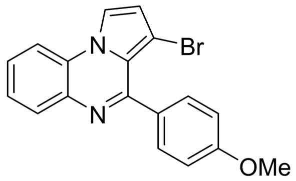

Mp. 167-169 °C. Light yellow solid (53.1 mg, 76%).

^{1}H NMR (400 MHz, $CDCl_3$) δ 8.00 (dd, J = 8.0, 1.6 Hz, 1H), 7.91 (d, J = 2.8 Hz, 1H), 7.80 (dd, J = 8.0, 1.6 Hz, 1H), 7.61 (d, J = 2.0 Hz, 1H), 7.59 (d, J = 2.0 Hz, 1H), 7.49 (td, J = 8.0, 1.6 Hz, 1H), 7.44 (td, J = 7.6, 1.2 Hz, 1H), 7.04 (d, J = 2.4 Hz, 1H), 7.02 (d, J = 2.0 Hz, 1H), 6.89 (d, J = 2.8 Hz, 1H), 3.89 (s, 3H).

^{13}C NMR (101 MHz, $CDCl_3$) δ 160.73, 154.54, 135.55, 131.08, 130.12, 129.87, 127.82, 126.85, 125.78, 121.94, 118.17, 114.64, 113.41, 113.07, 95.70, 55.45.

HRMS (APCI): m/z calcd for $C_{18}H_{13}BrN_2O$ $[M+H]^+$: 353.0284, found: 353.0280.

3-bromo-4-(4-chlorophenyl)pyrrolo[1,2-*a*]quinoxaline [3e]

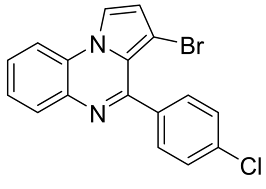

Mp. 199-201°C. Light yellow solid (49.5 mg, 70%).

^{1}H NMR (400 MHz, $CDCl_3$) δ 8.01 (dd, J = 8.0, 1.6 Hz, 1H), 7.96 (d, J = 2.8 Hz, 1H), 7.85 (dd, J = 8.0, 1.2 Hz, 1H), 7.60-7.57 (m, 2H), 7.54 (dd, J = 8.4,

1.6 Hz, 1H), 7.50-7.45 (m, 3H), 6.92 (d, *J* = 3.2 Hz, 1H).

^{13}C NMR (101 MHz, $CDCl_3$) δ 153.72, 135.92, 135.59, 135.51, 131.04, 130.40, 128.36, 128.33, 127.04, 126.03, 121.69, 118.33, 114.87, 113.22, 95.71 .

HRMS (APCI): m/z calcd for $C_{17}H_{10}BrClN_2$ $[M+H]^+$: 356.9789, found: 356.9762.

3-bromo-4-(4-bromophenyl)pyrrolo[1,2-*a*]quinoxaline [3f]

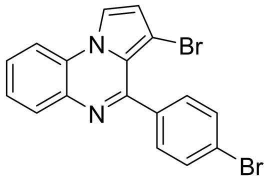

Mp. 202-205 ºC. Light yellow solid (45.9 mg, 58%).

^{1}H NMR (400 MHz, $CDCl_3$) δ 8.01 (dd, *J* = 8.0, 1.6 Hz, 1H), 7.97 (d, *J* = 2.8 Hz, 1H), 7.85 (dd, *J* = 8.0, 1.2 Hz, 1H), 7.67-7.62 (m, 2H), 7.58-7.53 (m, 2H), 7.52-7.50 (m, 1H), 7.49-7.46 (m, 1H), 6.92 (d, *J* = 2.8 Hz, 1H).

^{13}C NMR (101 MHz, $CDCl_3$) δ 153.69, 136.26, 135.39, 131.30, 131.26, 130.32, 128.39, 127.00, 126.05, 123.91, 121.59, 118.36, 114.95, 113.23, 95.82.

HRMS (APCI): m/z calcd for $C_{17}H_{10}Br_2N_2$ $[M+H]^+$: 400.9284, found: 400.9261.

3-bromo-4-(4-fluorophenyl)pyrrolo[1,2-*a*]quinoxaline [3g]

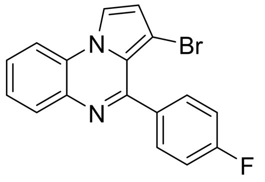

Mp. 174-176 ºC. Light yellow solid (59.3 mg, 87%).

^{1}H NMR (400 MHz, $CDCl_3$) δ 7.91 (dd, *J* = 8.0, 1.6 Hz, 1H), 7.85 (d, *J* = 2.8 Hz, 1H), 7.74 (dd, *J* = 8.0, 1.6 Hz, 1H), 7.57-7.49 (m, 2H), 7.44 (td, *J* = 8.0, 7.6, 1.6 Hz, 1H), 7.38 (td, *J* =8.0, 7.6, 1.6 Hz, 1H), 7.14-7.06 (m, 2H), 6.82 (d, *J* = 2.8 Hz, 1H.

^{13}C NMR (101 MHz, $CDCl_3$) δ 163.70 (d, *J* = 249.3 Hz), 153.81, 135.43, 133.47 (d, *J* = 3.3 Hz), 131.54 (d, *J* = 8.4 Hz), 130.26, 128.20, 126.95, 125.94, 121.75, 118.26, 115.08 (d, *J* = 21.9 Hz), 114.80, 113.15, 95.70.

^{19}F NMR (376 MHz, $CDCl_3$) δ -111.96.

HRMS (APCI): m/z calcd for $C_{17}H_{10}BrFN_2$ $[M+H]^+$: 341.0084, found:

341.0083.

3-bromo-4-(4-nitrophenyl)pyrrolo[1,2-*a*]quinoxaline [3h]

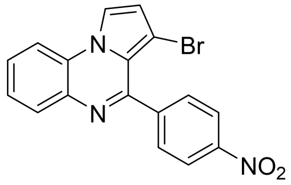

Mp. 244-249 ºC. Yellow solid (69.1 mg, 94%).

^{1}H NMR (400 MHz, $CDCl_3$) δ 8.38 (d, J = 8.8 Hz, 2H), 8.07-8.00 (m, 2H), 7.90 (dd, J = 8.4, 0.8 Hz, 1H), 7.83 (d, J = 8.4 Hz, 2H), 7.61 (td, J = 8.4, 7.2, 1.2 Hz, 1H), 7.53 (td, J = 8.4, 7.2, 1.2 Hz, 1H), 6.97 (d, J = 2.8 Hz, 1H).

^{13}C NMR (151 MHz, $CDCl_3$) δ 152.57, 148.62, 143.70, 135.34, 130.86, 130.58, 128.98, 127.13, 126.32, 123.37, 121.35, 118.53, 115.27, 113.38, 95.67.

HRMS (APCI): m/z calcd for $C_{17}H_{10}BrN_3O_2$ $[M+H]^+$: 368.0029, found: 368.0027.

3-bromo-4-(2,4-dichlorophenyl)pyrrolo[1,2-*a*]quinoxaline [3i]

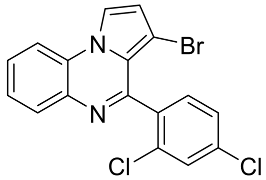

Mp. 137-141 ºC. Light yellow solid (63.3 mg, 81%).

^{1}H NMR (400 MHz, $CDCl_3$) δ 8.03 (dd, J = 8.0, 1.6 Hz, 1H), 7.97 (d, J = 2.8 Hz, 1H), 7.87 (dd, J = 8.0, 1.2 Hz, 1H), 7.59 (td, J = 8.0, 1.2 Hz, 1H), 7.54 (t, J = 1.2 Hz, 1H), 7.50 (td, J = 8.0, 1.2 Hz, 2H), 7.41 (d, J = 1.6 Hz, 2H), 6.91 (d, J = 3.2 Hz, 1H);

^{13}C NMR (101 MHz, $CDCl_3$) δ 151.39, 136.02, 134.94, 131.64, 130.35, 129.47, 128.88, 127.37, 126.23, 121.88, 118.17, 115.17, 113.41.

HRMS (APCI): m/z calcd for $C_{17}H_9BrCl_2N_2$ $[M+H]^+$: 390.9398, found: 390.9395.

3-bromo-1-phenylpyrrolo[1,2-*a*]quinoxaline [3j]

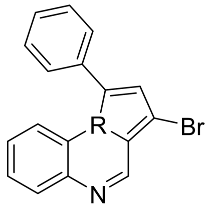

Mp. 153-158 ºC. Light yellow solid (49.8 mg, 39%).

^{1}H NMR (400 MHz, $CDCl_3$) δ 8.83 (s, 1H), 7.96 (dd, J = 8.0, 1.6 Hz, 1H), 7.51 (t, J = 3.2 Hz, 5H), 7.40-7.31 (m, 2H), 7.14 (td, J = 7.2, 1.6 Hz, 1H), 6.81 (s, 1H).

^{13}C NMR (101 MHz, $CDCl_3$) δ 144.35, 137.21, 132.99, 132.49, 130.33, 129.84, 129.36, 129.02, 128.52, 127.16, 125.67, 124.79, 118.76, 116.50, 95.96.

HRMS (APCI): m/z calcd for $C_{17}H_{11}BrN_2$ $[M+H]^+$: 323.0178, found: 323.0177.

3-bromo-1-(p-tolyl)pyrrolo[1,2-*a*]quinoxaline [3k]

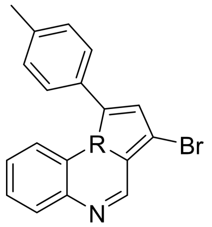

Mp. 118-123 °C. Light yellow solid (55.0 mg, 41%).

^{1}H NMR (400 MHz, $CDCl_3$) δ 8.81 (s, 1H), 7.95 (dd, J = 8.0, 1.6 Hz, 1H), 7.41-7.34 (m, 4H), 7.31 (d, J = 8.0 Hz, 2H), 7.14 (td, J = 8.0, 1.6 Hz, 1H), 6.77 (s, 1H), 2.48 (s, 3H).

^{13}C NMR (101 MHz, $CDCl_3$) δ 144.38, 139.40, 137.27, 132.62, 130.30, 130.03, 129.71, 128.63, 127.05, 125.58, 124.70, 118.63, 116.51, 95.82, 21.60.

HRMS (APCI): m/z calcd for $C_{18}H_{13}BrN_2$ $[M+H]^+$: 337.0335, found: 337.0333.

3-bromo-1-(4-methoxyphenyl)pyrrolo[1,2-*a*]quinoxaline [3l]

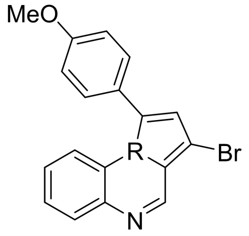

Mp. 148-150 °C. Light yellow solid (65.2 mg, 93%).

^{1}H NMR (400 MHz, $CDCl_3$) δ 8.80 (s, 1H), 7.94 (dd, J = 8.0, 1.6 Hz, 1H), 7.42-7.33 (m, 4H), 7.17-7.11 (m, 1H), 7.03 (d, J = 8.4 Hz, 2H), 6.75 (s, 1H), 3.91 (s, 3H).

^{13}C NMR (101 MHz, $CDCl_3$) δ 160.42, 144.34, 137.26, 132.37, 131.17, 130.28, 128.67, 127.06, 125.54, 125.12, 124.58, 118.57, 116.38, 114.42, 95.74, 55.53.

HRMS (APCI): m/z calcd for $C_{18}H_{13}BrN_2O$ $[M+H]^+$: 353.0284, found:

353.0283.

3-bromo-7-methylpyrrolo[1,2-*a*]quinoxaline [3m]

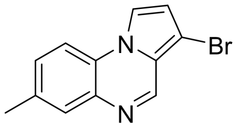

Mp. 174-179 ºC. Light yellow solid (51.0 mg, 98%).

^{1}H NMR (400 MHz, $CDCl_3$) δ 8.72 (s, 1H), 7.74 (dd, J = 7.2, 2.4 Hz, 2H), 7.64 (d, J = 8.4 Hz, 1H), 7.30 (dd, J = 8.4, 2.0 Hz, 1H), 6.83 (d, J = 2.8 Hz, 1H), 2.47 (s, 3H).

^{13}C NMR (101 MHz, $CDCl_3$) δ 143.86, 135.79, 135.71, 130.08, 129.42, 125.16, 123.73, 115.98, 113.89, 113.02, 94.65, 21.20.

HRMS (APCI): m/z calcd for $C_{12}H_9BrN_2$ $[M+H]^+$: 261.0022, found: 221.0019.

3-bromo-7-chloropyrrolo[1,2-*a*]quinoxaline [3n]

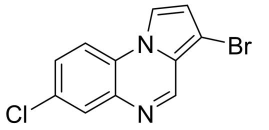

Mp. 167-172 ºC. Light yellow solid (33.9 mg, 61%).

^{1}H NMR (400 MHz, $CDCl_3$) δ 8.78 (s, 1H), 7.96 (d, J = 2.4 Hz, 1H), 7.82 (dd, J = 2.8, 0.8 Hz, 1H), 7.74 (d, J = 8.8 Hz, 1H), 7.49 (dd, J = 8.8, 2.4 Hz, 1H), 6.91 (d, J = 2.8 Hz, 1H).

^{13}C NMR (101 MHz, $CDCl_3$) δ 145.02, 136.68, 131.24, 129.73, 128.51, 126.05, 123.82, 116.86, 114.69, 114.61, 96.10.

HRMS (APCI): m/z calcd for $C_{11}H_6BrClN_2$ $[M+H]^+$: 280.9476, found: 280.9473.

3-bromo-4-(4-methoxyphenyl)-7-methylpyrrolo[1,2-*a*]quinoxaline [3o]

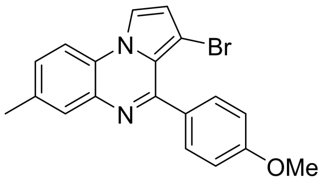

Mp. 162-167 ºC. Light yellow solid (47.4 mg, 65%)

^{1}H NMR (400 MHz, $CDCl_3$) δ 7.89 (d, J = 2.8 Hz, 1H), 7.82 (s, 1H), 7.71 (d, J = 8.4 Hz, 1H), 7.60 (d, J = 2.0 Hz, 1H), 7.58 (d, J = 2.0 Hz, 1H), 7.32 (dd, J = 8.4, 2.0 Hz, 1H), 7.03 (d, J = 2.0 Hz, 1H), 7.02 (d, J = 2.0 Hz, 1H), 6.88 (d, J = 3.2 Hz, 1H), 3.89 (s, 3H), 2.48 (s, 3H).

^{13}C NMR (101 MHz, $CDCl_3$) δ 160.79, 154.48, 135.80, 135.33, 131.16, 129.82, 129.08, 124.79, 121.86, 118.06, 114.60, 113.43, 112.86, 95.66, 55.49, 21.23.

HRMS (APCI): m/z calcd for $C_{19}H_{15}BrN_2O$ $[M+H]^+$: 367.0441, found: 367.0437.

3-bromo-4-(4-bromophenyl)-7-methylpyrrolo[1,2-*a*]quinoxaline [3p]

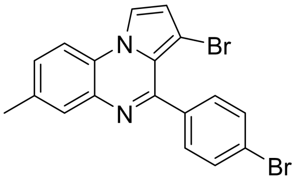

Mp. 216-219 ºC. Light yellow solid (39.5 mg, 51%).

^1H NMR (400 MHz, $CDCl_3$) δ 7.68 (d, J = 3.2 Hz, 1H), 7.57 (s, 1H), 7.49 (d, J = 8.4 Hz, 1H), 7.39 (dd, J = 6.4, 2.0 Hz, 2H), 7.27 (dd, J = 6.4, 2.0 Hz, 2H), 7.12 (dd, J = 8.4, 2.0 Hz, 1H), 6.65 (d, J = 2.8 Hz, 1H), 2.25 (s, 3H).

^{13}C NMR (101 MHz, $CDCl_3$) δ 153.60, 136.40, 135.99, 135.36, 131.23, 130.09, 129.53, 124.90, 123.82, 121.52, 118.09, 114.71, 112.95, 95.41, 21.25.

HRMS (APCI): m/z calcd for $C_{18}H_{12}Br_2N_2$ $[M+H]^+$: 414.9440, found: 414.9436.

3-bromo-8-methylpyrrolo[1,2-*a*]quinoxaline [3q]

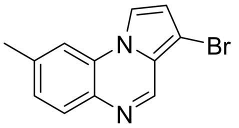

Mp. 169-174 ºC. Light yellow solid (51.5 mg, 98%).

^1H NMR (400 MHz, $CDCl_3$) δ 8.72 (s, 1H), 7.84 (d, J = 8.4 Hz, 1H), 7.79 (d, J = 3.2 Hz, 1H), 7.58 (d, J = 2.0 Hz, 1H), 7.26 (dd, J = 8.0, 2.0 Hz, 1H), 6.86 (d, J = 2.8 Hz, 1H), 2.53 (s, 3H).

^{13}C NMR (101 MHz, $CDCl_3$) δ 143.03, 139.08, 133.78, 130.02, 127.22, 127.13, 123.92, 116.23, 113.86, 113.42, 94.69, 21.96.

HRMS (APCI): m/z calcd for $C_{12}H_9BrN_2$ $[M+H]^+$: 261.0022, found: 261.0020.

3-bromo-8-chloropyrrolo[1,2-*a*]quinoxaline [3r]

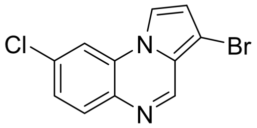

Mp. 169-174 ºC. Light yellow solid (28.7 mg, 51%).

^1H NMR (400 MHz, $CDCl_3$) δ 8.82 (s, 1H), 8.04 (d, J = 8.8 Hz, 1H), 7.88 (d, J

= 2.8 Hz, 1H), 7.85 (d, J = 2.0 Hz, 1H), 7.47 (dd, J = 8.4, 2.0 Hz, 1H), 7.00 (d, J = 2.8 Hz, 1H).

^{13}C NMR (101 MHz, $CDCl_3$) δ 143.51, 134.45, 130.95, 127.97, 127.01, 126.74, 123.73, 117.50, 115.46, 115.36, 113.81.

HRMS (APCI): m/z calcd for $C_{11}H_6BrClN_2$ $[M+H]^+$: 280.9476, found: 280.9474.

3-bromo-8-fluoropyrrolo[1,2-*a*]quinoxaline [3s]

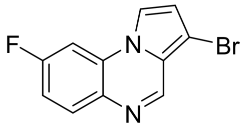

Mp. 164-166 °C. Light yellow solid (43.3 mg, 84%).

^{1}H NMR (400 MHz, $CDCl_3$) δ 8.78 (s, 1H), 8.02 (dd, J =8.8, 5.6 Hz, 1H), 7.78 (d, J = 3.2 Hz, 1H), 7.51 (dd, J = 8.8, 2.4 Hz, 1H), 7.22 (td, J = 9.2, 2.8 Hz, 1H), 6.95 (d, J = 2.8 Hz, 1H).

^{13}C NMR (151 MHz, $CDCl_3$) δ 161.94 (d, J = 250.1 Hz), 143.25, 132.33 (d, J = 9.6 Hz), 116.98, 114.50, 114.10, 100.55, 100.37.

^{19}F NMR (565 MHz, $CDCl_3$) δ -109.16.

HRMS (APCI): m/z calcd for $C_{11}H_6BrFN_2$ $[M+H]^+$: 264.9771, found: 264.9769.

1-bromo-3-phenylpyrrolo[1,2-*a*]quinoxaline [3t]

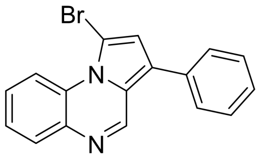

Mp. 137-141°C. Light yellow solid (58.2 mg, 90%).

^{1}H NMR (400 MHz, $CDCl_3$) δ 9.27 (dd, J = 8.0, 2.0 Hz, 1H), 8.90 (s, 1H), 7.98 (dd, J = 7.2, 2.4 Hz, 1H), 7.57 (d, J = 7.2 Hz, 2H), 7.53-7.46 (m, 4H), 7.40 (t, J = 7.2 Hz, 1H), 7.03 (s, 1H).

^{13}C NMR (101 MHz, $CDCl_3$) δ 144.08, 137.05, 133.00, 129.85, 129.23, 128.72, 127.86, 127.09, 126.11, 124.81, 124.70, 119.02, 115.61, 100.11.

HRMS (APCI): m/z calcd for $C_{17}H_{11}BrN_2$ $[M+H]^+$: 323.0178, found: 323.0177.

1-bromo-3-(p-tolyl)pyrrolo[1,2-*a*]quinoxaline [3u]

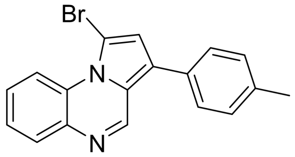

Mp. 150-155 ºC. Light yellow solid (59.4 mg, 88%).

^{1}H NMR (400 MHz, $CDCl_3$) δ 9.26 (dd, J = 8.0, 2.0 Hz, 1H), 8.88 (s, 1H), 7.98 (dd, J = 7.2, 2.0 Hz, 1H), 7.54-7.45 (m, 4H), 7.30 (d, J = 7.6 Hz, 2H), 7.01 (s, 1H), 2.43 (s, 3H).

^{13}C NMR (101 MHz, $CDCl_3$) δ 144.28, 137.76, 137.20, 130.10, 129.94, 129.90, 129.35, 128.58, 126.99, 126.03, 124.76, 124.67, 118.89, 115.59, 99.91, 21.37.

HRMS (APCI): m/z calcd for $C_{18}H_{13}BrN_2$ $[M+H]^+$: 337.0335, found: 337.0333.

4-(1-bromopyrrolo[1,2-*a*]quinoxalin-3-yl)benzonitrile [3v]

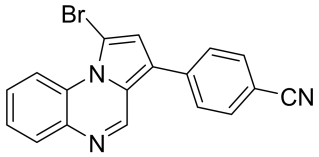

Mp. 240-242 ºC. Light yellow solid (43.9mg, 63%).

^{1}H NMR (400 MHz, $CDCl_3$) δ 9.32 (dd, J = 8.0, 2.0 Hz, 1H), 8.90 (s, 1H), 8.04 (dd, J = 7.2, 2.0 Hz, 1H), 7.79 (d, J = 8.4 Hz, 2H), 7.68 (d, J = 8.4 Hz, 2H), 7.57 (pd, J = 7.2, 2.0 Hz, 2H), 7.08 (s, 1H).

^{13}C NMR (101 MHz, $CDCl_3$) δ 143.08, 137.69, 133.06, 129.98, 129.10, 127.72, 126.66, 119.12, 118.81, 115.77, 111.40, 101.17.

HRMS (APCI): m/z calcd for $C_{18}H_{10}BrN_3$ $[M+H]^+$: 348.0131, found: 348.0129.

1,3-dibromopyrrolo[1,2-*a*]quinoxaline [4a]

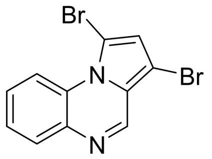

Mp. 157-162 ºC. White solid (52.1 mg, 80%).

^{1}H NMR (400 MHz, $CDCl_3$) *I* 9.18-9.23 (m, 1H), 8.71 (s, 1H), 8.03-7.96 (m, 1H), 7.51-7.53 (m, 2H), 6.91 (s, 1H).

^{13}C NMR (101 MHz, $CDCl_3$) δ 143.32, 137.00, 130.40, 128.87, 127.42, 126.53, 125.97, 120.90, 115.34, 99.42, 96.23.

HRMS (APCI): m/z calcd for $C_{11}H_6Br_2N_2$ $[M+H]^+$: 324.8971, found: 324.8968.

1,3-dibromo-4-phenylpyrrolo[1,2-*a*]quinoxaline [4b]

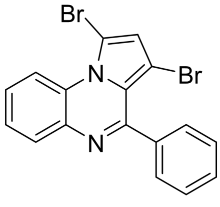

Mp. 151-153 ºC. White solid (49.5 mg, 62%).

^{1}H NMR (400 MHz, $CDCl_3$) δ 9.21-9.13 (m, 1H), 7.96-7.90 (m, 1H), 7.51-7.45 (m, 2H), 7.44-7.38 (m, 5H), 6.84 (s, 1H).

^{13}C NMR (101 MHz, $CDCl_3$) δ 154.29, 137.32, 136.77, 130.41, 129.43, 129.41, 128.43, 128.18, 126.94, 126.42, 124.38, 123.03, 115.29, 99.87, 96.74.

HRMS (APCI): m/z calcd for $C_{17}H_{10}Br_2N_2$ $[M+H]^+$: 400.9284, found: 400.9280.

1,3-dibromo-4-(p-tolyl)pyrrolo[1,2-*a*]quinoxaline [4c]

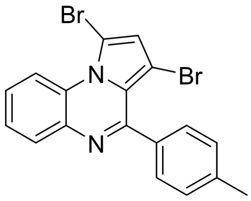

Mp. 159-162 ºC. Light yellow solid (46.5 mg, 56%).

^{1}H NMR (400 MHz, $CDCl_3$) δ 9.29-9.21 (m, 1H), 8.05-7.99 (m, 1H), 7.53-7.47 (m, 3H), 7.46 (s, 1H), 7.31 (s, 1H), 7.29 (s, 1H), 6.93 (s, 1H), 2.45 (s, 3H).

^{13}C NMR (101 MHz, $CDCl_3$) δ 155.01, 139.42, 135.52, 134.50, 130.24, 129.48, 128.72, 127.98, 126.97, 125.86, 121.94, 118.25, 114.69, 113.13, 95.88, 21.66.

HRMS (APCI): m/z calcd for $C_{18}H_{12}Br_2N_2$ $[M+H]^+$: 414.9440, found: 414.9435.

1,3-dibromo-4-(4-methoxyphenyl)pyrrolo[1,2-*a*]quinoxaline [4d]

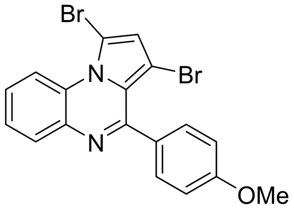

Mp. 159-162 ºC. White solid (43.2 mg, 50%)

^{1}H NMR (400 MHz, $CDCl_3$) δ 9.22-9.15 (m, 1H), 7.95-7.97 (m, 1H), 7.46 (d, J = 2.0 Hz, 1H), 7.45-7.40 (m, 3H), 6.95 (d, J = 2.0 Hz, 1H), 6.93 (d, J = 2.0 Hz, 1H), 6.87 (s, 1H), 3.81 (s, 3H).

^{13}C NMR (101 MHz, $CDCl_3$) δ 160.89, 154.01, 136.68, 131.05, 130.17, 128.33, 126.82, 126.45, 124.54, 123.14, 115.31, 113.60, 100.01, 97.18, 55.52.

HRMS (APCI): m/z calcd for $C_{18}H_{12}Br_2N_2O$ $[M+H]^+$: 430.9389, found: 430.9385.

1,3-dibromo-4-(4-chlorophenyl)pyrrolo[1,2-*a*]quinoxaline [4e]

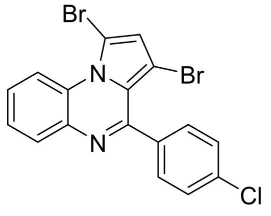

Mp. 189-193 ºC. White solid (45.2 mg, 53%).

^{1}H NMR (400 MHz, $CDCl_3$) δ 9.31-9.22 (m, 1H), 8.04-7.97 (m, 1H), 7.51-7.55 (m, 4H), 7.46-7.49 (m, 2H), 6.94 (s, 1H).

^{13}C NMR (101 MHz, $CDCl_3$) δ 153.07, 136.59, 135.71, 130.96, 130.36, 128.45, 128.43, 127.22, 126.58, 124.16, 123.15, 115.36, 100.26, 96.80.

HRMS (APCI): m/z calcd for $C_{17}H_9Br_2ClN_2$ $[M+H]^+$: 434.8894, found: 434.8890.

1,3-dibromo-4-(4-bromophenyl)pyrrolo[1,2-*a*]quinoxaline [4f]

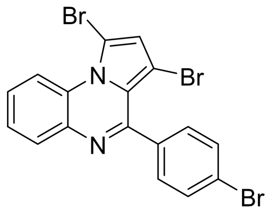

Mp. 207-211ºC. White solid (49.5 mg, 53%).

^{1}H NMR (400 MHz, $CDCl_3$) δ 9.32-9.23 (m, 1H), 8.06-7.96 (m, 1H), 7.63 (d, J = 8.0 Hz, 2H), 7.58-7.49 (m, 2H), 7.46 (d, J = 8.0 Hz, 2H), 6.95 (s, 1H).

^{13}C NMR (101 MHz, $CDCl_3$) δ 153.06, 136.53, 136.06, 131.39, 131.21, 130.33, 128.41, 127.25, 126.60, 124.08, 124.00, 123.17, 115.37, 100.33, 96.87.

HRMS (APCI): m/z calcd for $C_{17}H_9Br_3N_2$ $[M+H]^+$: 478.8389, found: 478.8386.

1,3-dibromo-4-(4-fluorophenyl)pyrrolo[1,2-*a*]quinoxaline [4g]

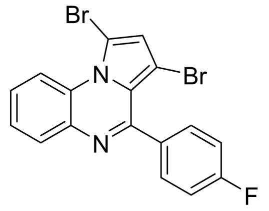

Mp. 168-169 ºC. White solid (45.7 mg, 55%).

^{1}H NMR (400 MHz, $CDCl_3$) δ 9.31-9.23 (m, 1H), 8.06-8.00 (m, 1H), 7.60-7.48 (m, 4H), 7.19 (t, J = 8.8 Hz, 2H), 6.95 (s, 1H).

^{13}C NMR (101 MHz, $CDCl_3$) δ 165.04, 162.57, 153.24, 131.56, 131.47, 130.29, 128.44, 127.18, 126.59, 124.30, 123.20, 115.41, 115.37, 115.19, 100,40, 97.24.

^{19}F NMR (376 MHz, $CDCl_3$) δ -111.77 (s).

HRMS (APCI): m/z calcd for $C_{17}H_9Br_2FN_2$ $[M+H]^+$: 418.9189, found: 418.9185.

1,3-dibromo-4-(4-nitrophenyl)pyrrolo[1,2-*a*]quinoxaline [4h]

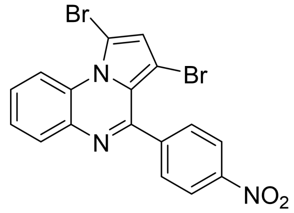

Mp. 159-162 ºC. Yellow solid (54.5 mg, 61%)

^{1}H NMR (400 MHz, $CDCl_3$) δ 9.31 (dd, J = 8.0, 2.0 Hz, 1H), 8.37 (d, J = 8.4 Hz, 2H), 8.03 (dd, J = 7.6, 2.0 Hz, 1H), 7.77 (d, J = 8.0 Hz, 2H), 7.57 (pd, J = 7.2, 1.6 Hz, 2H), 6.99 (s, 1H).

^{13}C NMR (151 MHz, $CDCl_3$) δ 151.92, 148.68, 143.48, 136.44, 130.81, 130.54, 128.54, 127.81, 126.85, 123.78, 123.46, 123.30, 115.51, 100.83, 96.72.

HRMS (APCI): m/z calcd for $C_{17}H_9Br_2N_3O_2$ $[M+H]^+$: 445.9134, found: 445.9131.

1,3-dibromo-4-(2,4-dichlorophenyl)pyrrolo[1,2-*a*]quinoxaline [4i]

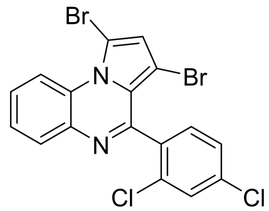

Mp. 145-150 ºC. Light yellow solid (71.1 mg, 78%).

^{1}H NMR (400 MHz, $CDCl_3$) δ 9.27 (dd, J = 8.0, 1.6 Hz, 1H), 8.01 (dd, J = 7.6, 2.0 Hz, 1H), 7.56-7.50 (m, 3H), 7.41 (dd, J = 8.0, 1.6 Hz, 1H), 7.38 (d, J = 8.4

Hz, 1H), 6.92 (s, 1H).

^{13}C NMR (151 MHz, CDCl$_3$) δ 150.72, 136.48, 136.00, 134.96, 131.62, 130.53, 130.17, 129.42, 128.76, 127.56, 127.38, 126.58, 124.15, 122.79, 115.36, 100.16, 96.64.

HRMS (APCI): m/z calcd for $C_{17}H_8Br_2Cl_2N_2$ [M+H]$^+$: 468.8504, found: 468.8500.

1,3-dibromo-7-methylpyrrolo[1,2-*a*]quinoxaline [4j]

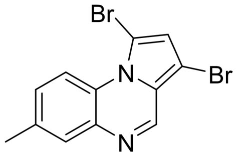

Mp. 149-151 ºC. Light yellow solid (59.1 mg, 87%).

^{1}H NMR (400 MHz, CDCl$_3$) δ 8.99 (d, J = 8.8 Hz, 1H), 8.63 (s, 1H), 7.74-7.70 (m, 1H), 7.28 (dd, J = 8.8, 2.0 Hz, 1H), 6.84 (s, 1H), 2.47 (s, 3H).

^{13}C NMR (101 MHz, CDCl$_3$) δ 143.18, 136.95, 136.42, 130.14, 128.38, 126.58, 125.79, 120.43, 114.94, 98.99, 95.75, 77.48, 77.16, 76.84, 21.06.

HRMS (APCI): m/z calcd for $C_{12}H_8Br_2N_2$ [M+H]$^+$: 338.9127, found: 338.9124.

1,3-dibromo-7-chloropyrrolo[1,2-*a*]quinoxaline [4k]

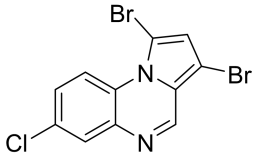

Mp. 187-190 ºC. Light yellow solid (36.3 mg, 51%).

^{1}H NMR (600 MHz, CDCl$_3$) δ 9.22 (d, J = 2.2 Hz, 1H), 9.22 (d, J = 2.4 Hz, 1H), 8.69 (s, 1H), 7.92 (d, J = 8.4 Hz, 1H), 7.47 (dd, J = 9.0, 2.4 Hz, 1H), 6.95 (s, 1H).

^{13}C NMR (151 MHz, CDCl$_3$) δ 143.53, 135.79, 132.81, 131.48, 129.24, 126.93, 125.93, 121.33, 115.42, 99.76, 96.69.

HRMS (APCI): m/z calcd for $C_{11}H_5Br_2ClN_2$ [M+H]$^+$: 358.8581, found: 358.8587.

1,3-dibromo-8-methylpyrrolo[1,2-*a*]quinoxaline [4l]

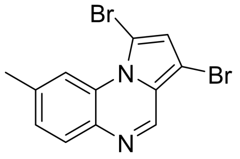

Mp. 166-171 ºC. Light yellow solid (48.1 mg, 71%).

^{1}H NMR (400 MHz, $CDCl_3$) δ 8.64 (s, 1H), 8.64 (s, 1H), 7.86 (d, J = 8.4 Hz, 1H), 7.31 (dd, J = 8.4, 1.6 Hz, 1H), 6.88 (s, 1H), 2.53 (s, 3H).

^{13}C NMR (101 MHz, $CDCl_3$) δ 142.25, 138.01, 134.75, 129.90, 128.62, 127.71, 125.97, 120.85, 115.33, 99.09, 95.95, 22.28.

HRMS (APCI): m/z calcd for $C_{12}H_8Br_2N_2$ $[M+H]^+$: 338.9127, found: 338.9125.

1,3-dibromo-8-chloropyrrolo[1,2-*a*]quinoxaline [4m]

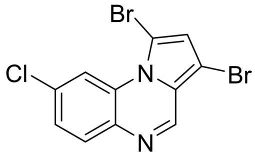

Mp. 197-199 ºC. Light yellow solid (62.0 mg, 86%).

^{1}H NMR (600 MHz, $CDCl_3$) δ 9.15 (d, J = 9.0 Hz, 1H), 8.72 (s, 1H), 7.99 (d, J = 2.4 Hz, 1H), 7.49 (dd, J = 9.6, 2.4 Hz, 1H), 6.95 (s, 1H).

^{13}C NMR (151 MHz, $CDCl_3$) δ 144.50, 138.36, 131.79, 129.79, 127.43, 127.31, 125.91, 121.12, 116.41, 99.77, 96.78.

HRMS (APCI): m/z calcd for $C_{11}H_5Br_2ClN_2$ $[M+H]^+$: 358.8581, found: 358.8586.

1,3-dibromo-8-fluoropyrrolo[1,2-*a*]quinoxaline [4n]

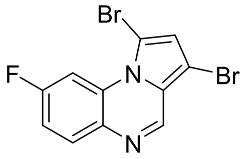

Mp. 184-186 ºC. Light yellow solid (55.1 mg, 81%).

^{1}H NMR (400 MHz, $CDCl_3$) δ 8.93 (dd, J = 10.8, 2.4 Hz, 1H), 8.66 (s, 1H), 7.97 (dd, J = 8.8, 6.0 Hz, 1H), 7.27-7.21 (m, 1H), 6.93 (s, 1H).

^{13}C NMR (151 MHz, $CDCl_3$) δ 160.53 (d, J = 248.6 Hz), 142.47, 133.51, 131.86 (d, J = 9.8 Hz), 125.59, 121.30, 114.48, 114.33, 102.57 (d, J = 29.8 Hz), 99.59, 96.57.

HRMS (APCI): m/z calcd for $C_{11}H_5Br_2FN_2$ $[M+H]^+$: 342.8876, found: 342.8872.

1,3-dibromo-4-(4-methoxyphenyl)-7-methylpyrrolo[1,2-*a*]quinoxaline [4o]

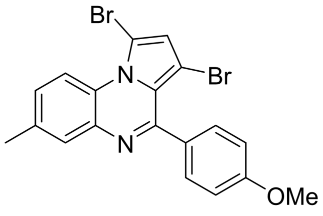
Mp. 166-168 ºC. Light yellow solid (39.1mg, 42%).

^{1}H NMR (400 MHz, $CDCl_3$) *δ* 7.89 (d, *J* = 2.8 Hz, 1H), 7.82 (s, 1H), 7.71 (d, *J* = 8.4 Hz, 1H), 7.60 (d, *J* = 2.0 Hz, 1H), 7.58 (d, *J* = 2.0 Hz, 1H), 7.32 (dd, *J* = 8.4, 2.0 Hz, 1H), 7.03 (d, *J* = 2.0 Hz, 1H), 7.02 (d, *J* = 1.8 Hz, 1H), 6.88 (d, *J* = 3.2 Hz, 1H), 3.89 (s, 3H), 2.48 (s, 3H).

^{13}C NMR (101 MHz, $CDCl_3$) *δ* 160.79, 153.92, 136.67, 136.36, 131.02, 129.97, 127.88, 126.15, 124.40, 122.73, 115.00, 113.54, 99.66, 96.52, 55.50, 21.05.

HRMS (APCI): m/z calcd for $C_{19}H_{14}Br_2N_2O$ $[M+H]^+$: 444.9546, found: 444.9542.

1,3-dibromo-4-(4-bromophenyl)-7-methylpyrrolo[1,2-*a*]quinoxaline [4q]

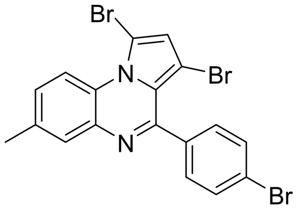
Mp. 124-129 ºC. Light yellow solid (73.7mg, 75%).

^{1}H NMR (400 MHz, $CDCl_3$) *δ* 9.14 (d, *J* = 8.8 Hz, 1H), 7.83 (s, 1H), 7.64 (d, *J* = 2.0 Hz, 1H), 7.62 (d, *J* = 2.0 Hz, 1H), 7.46 (d, *J* = 2.0 Hz, 1H), 7.44 (d, *J* = 2.0 Hz, 1H), 7.35 (dd, *J* = 8.8, 1.6 Hz, 1H), 6.93 (s, 1H), 2.49 (s, 3H).

^{13}C NMR (151 MHz, $CDCl_3$) *δ* 153.04, 136.59, 136.33, 131.36, 131.22, 130.21, 128.35, 126.29, 124.01, 123.89, 122.80, 115.11, 99.98, 96.44, 21.06.

HRMS (APCI): m/z calcd for $C_{18}H_{11}Br_3N_2$ $[M+H]^+$: 492.8545, found: 492.8542.

3-phenylpyrrolo[1,2-*a*]quinoxaline [3aa]

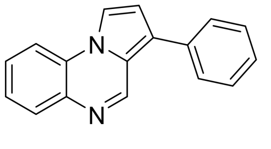
Mp. 178-180 ºC. Light yellow solid (42.7 mg, 88%).

^{1}H NMR (400 MHz, $CDCl_3$) *δ* 9.04 (s, 1H), 7.97 (dd, *J* = 8.0, 1.6 Hz, 1H), 7.93 (d, *J* = 2.8 Hz, 1H), 7.84 (dd, *J* = 8.0, 1.2 Hz, 1H), 7.65 (d, *J* = 7.2 Hz, 2H),

7.55-7.42 (m, 4H), 7.37 (t, *J* = 7.2 Hz, 1H), 7.02 (d, *J* = 2.8 Hz, 1H).

^{13}C NMR (101 MHz, $CDCl_3$) δ 145.16, 136.15, 134.49, 130.08, 129.15, 128.36, 128.08, 128.03, 127.19, 125.49, 123.88, 122.75, 114.28, 113.84, 113.80.

HRMS (APCI): m/z calcd for $C_{17}H_{12}N_2$ $[M+H]^+$: 245.1073, found: 245.1071.

3-bromo-1-(p-tolyl)pyrrolo[1,2-*a*]quinoxaline [3ab]

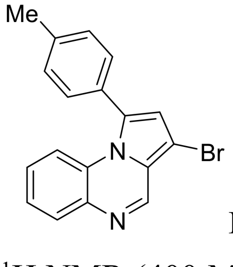

Mp. 118-123 ºC. Light yellow solid (56.9 mg, 85%).

^{1}H NMR (400 MHz, $CDCl_3$) δ 8.82 (s, 1H), 7.98 (d, *J* = 8.4 Hz, 1H), 7.35-7.41 (m, 4H), 7.32 (d, *J* = 8.0 Hz, 2H), 7.18-7.13 (m, 1H), 6.79 (s, 1H), 2.49 (s, 3H).

^{13}C NMR (101 MHz, $CDCl_3$) δ 144.00, 139.55, 136.70, 133.10, 129.95, 129.88, 129.76, 129.69, 128.59, 127.22, 125.74, 124.65, 118.93, 116.57, 96.56, 21.61.

HRMS (APCI): m/z calcd for $C_{18}H_{13}BrN_2$ $[M+H]^+$: 337.0335, found: 337.0331.

3-bromo-1-chloropyrrolo[1,2-*a*]quinoxaline [3ac]

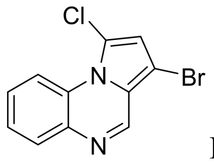

Mp 136-138 ºC. Light yellow solid (53.9 mg, 96%).

^{1}H NMR (400 MHz, $CDCl_3$) δ 8.93 (dd, *J* = 6.4, 3.6 Hz, 1H), 8.68 (s, 1H), 7.95 (dd, *J* = 6.4, 3.2 Hz, 1H), 7.48 (dd, *J* = 6.4, 3.2 Hz, 2H), 6.78 (s, 1H).

^{13}C NMR (101 MHz, $CDCl_3$) δ 143.51, 137.12, 130.42, 128.48, 127.57, 126.41, 124.31, 116.69, 115.76, 115.67, 95.02.

HRMS (APCI): m/z calcd for $C_{11}H_6BrClN_2$ $[M+H]^+$: 280.9476, found: 280.9473.

4. Copies of Spectra

3-bromopyrrolo[1,2-*a*]quinoxaline [3a]

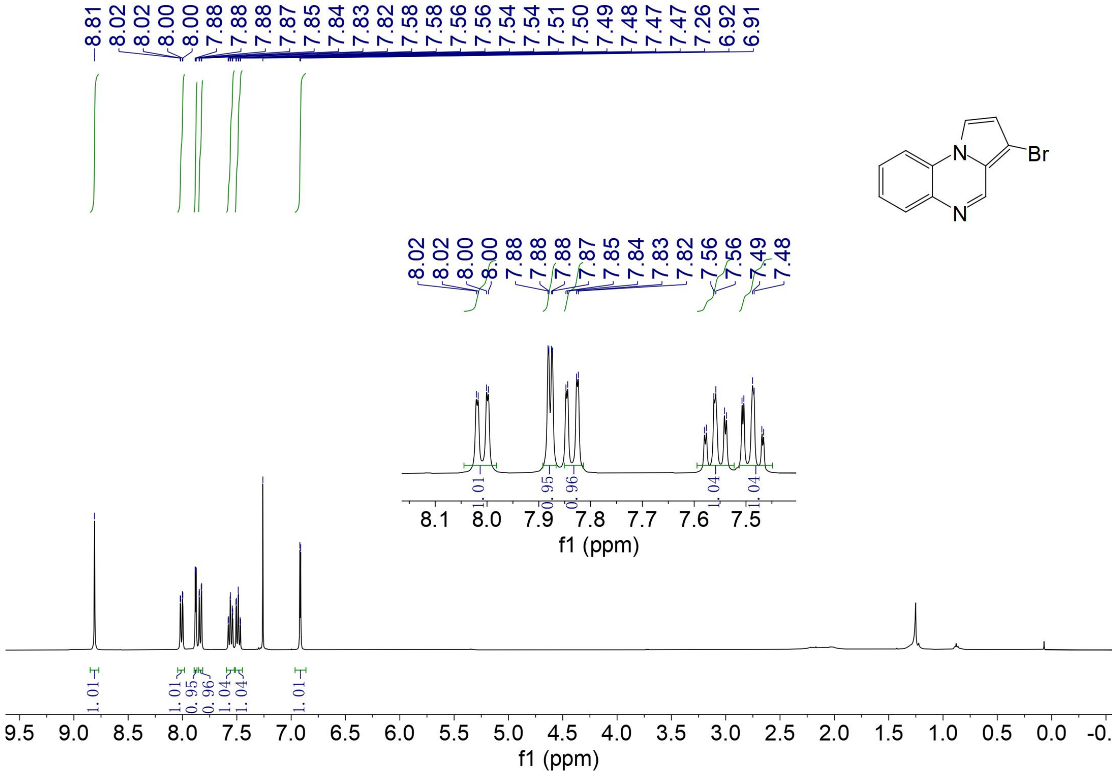

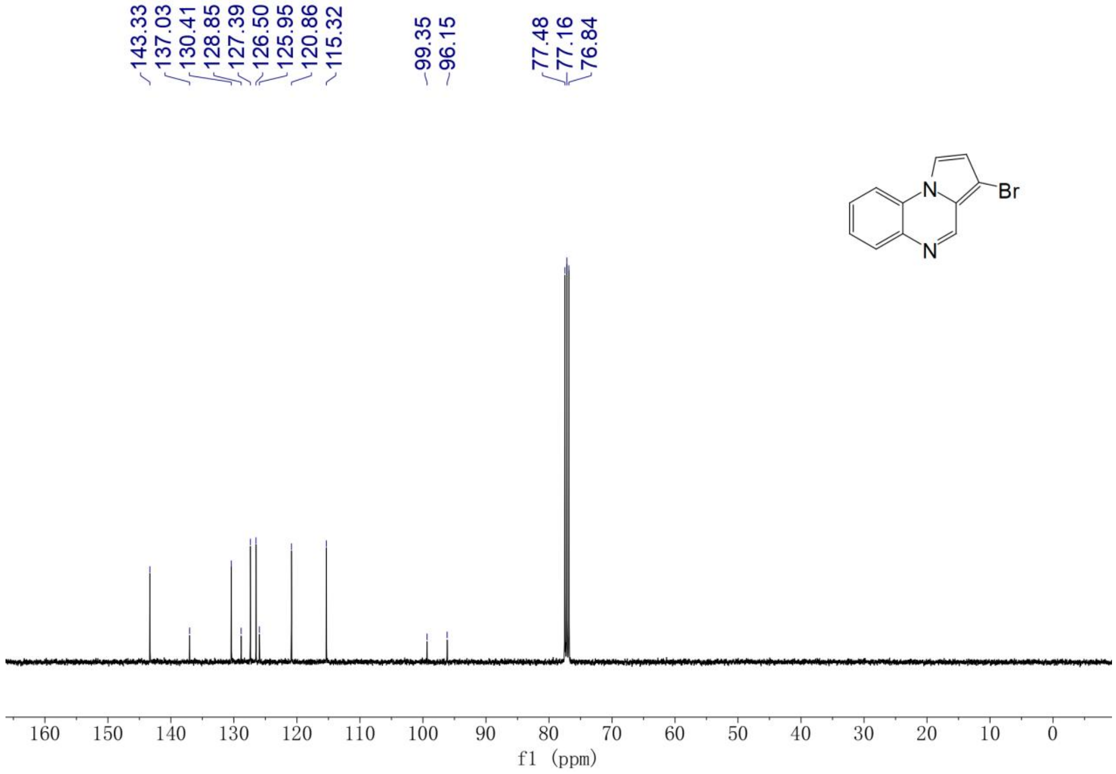

3-bromo-4-phenylpyrrolo[1,2-*a*]quinoxaline [3b]

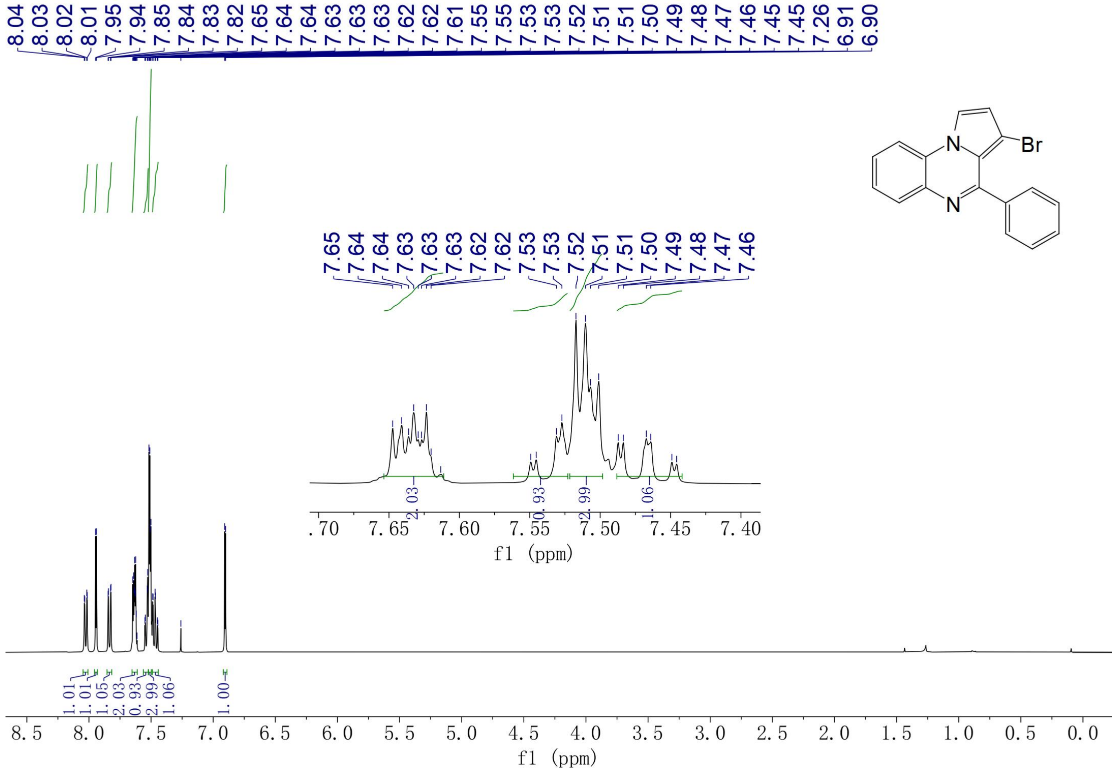

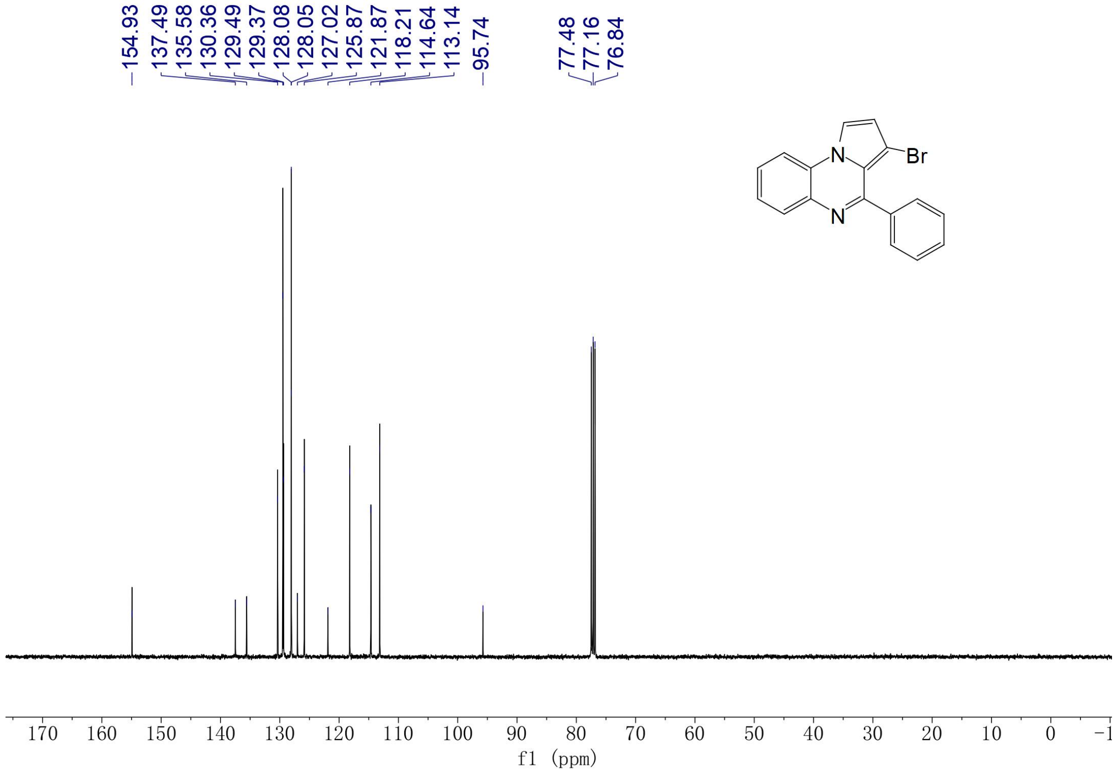

3-bromo-4-(p-tolyl)pyrrolo[1,2-*a*]quinoxaline [3c]

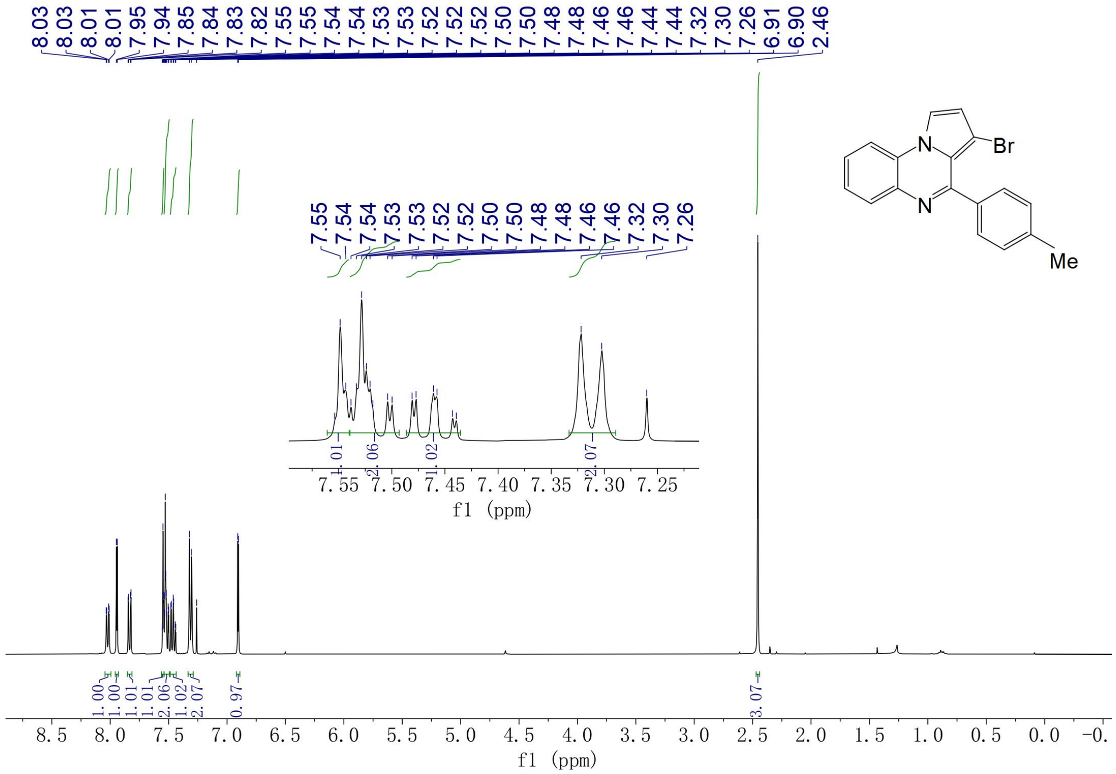

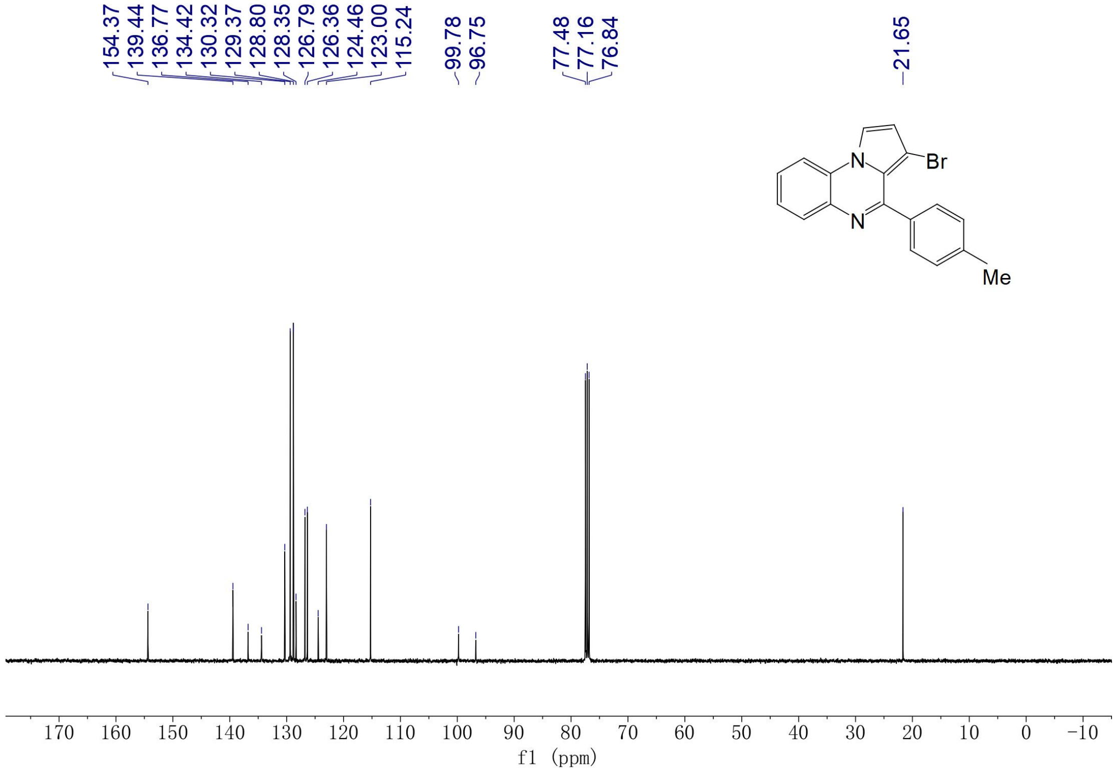

3-bromo-4-(4-methoxyphenyl)pyrrolo[1,2-*a*]quinoxaline [3d]

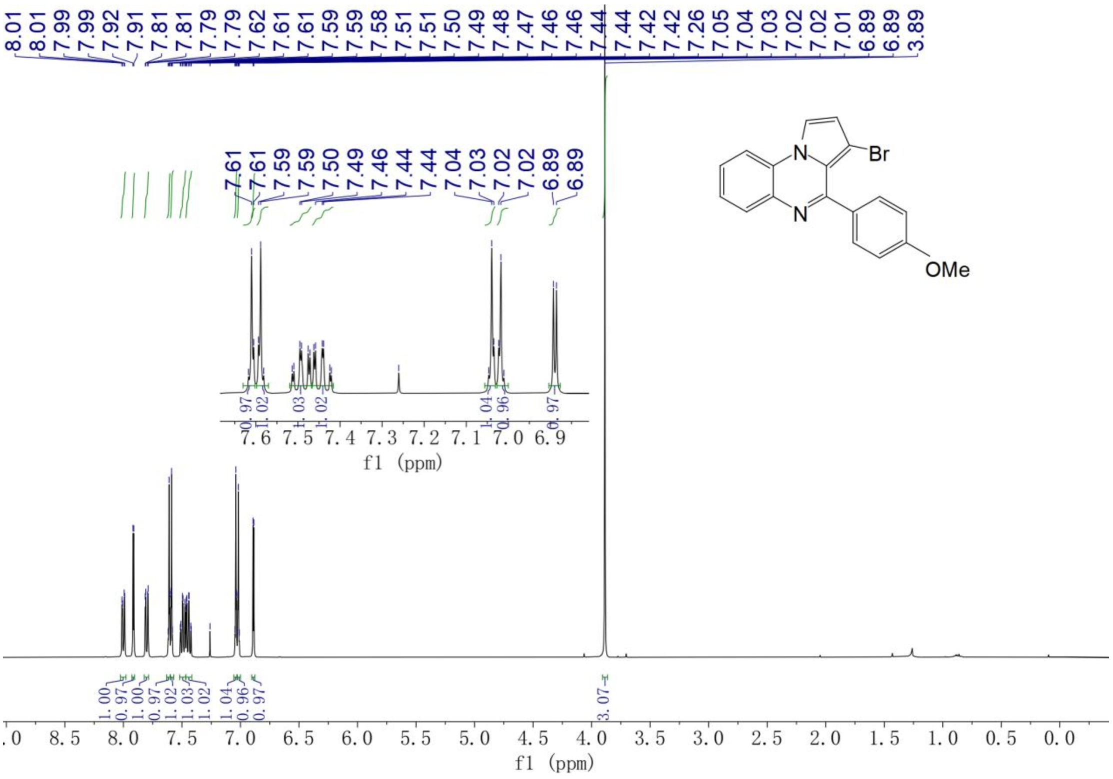

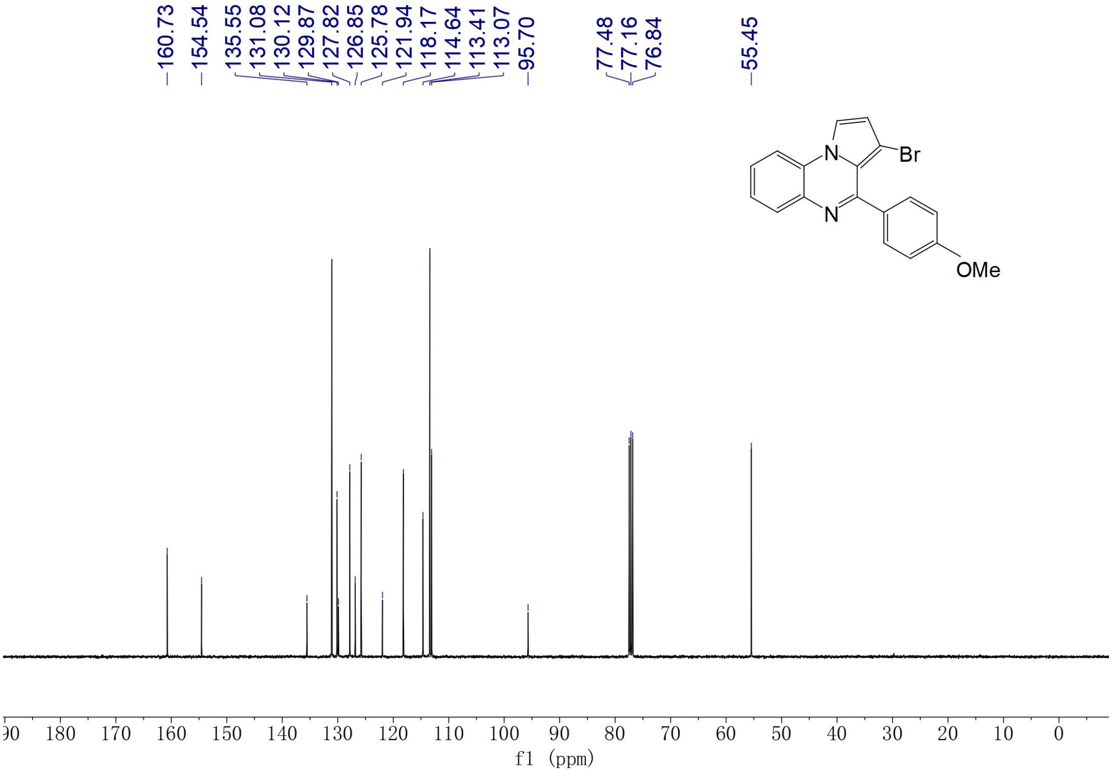

3-bromo-4-(4-chlorophenyl)pyrrolo[1,2-*a*]quinoxaline [3e]

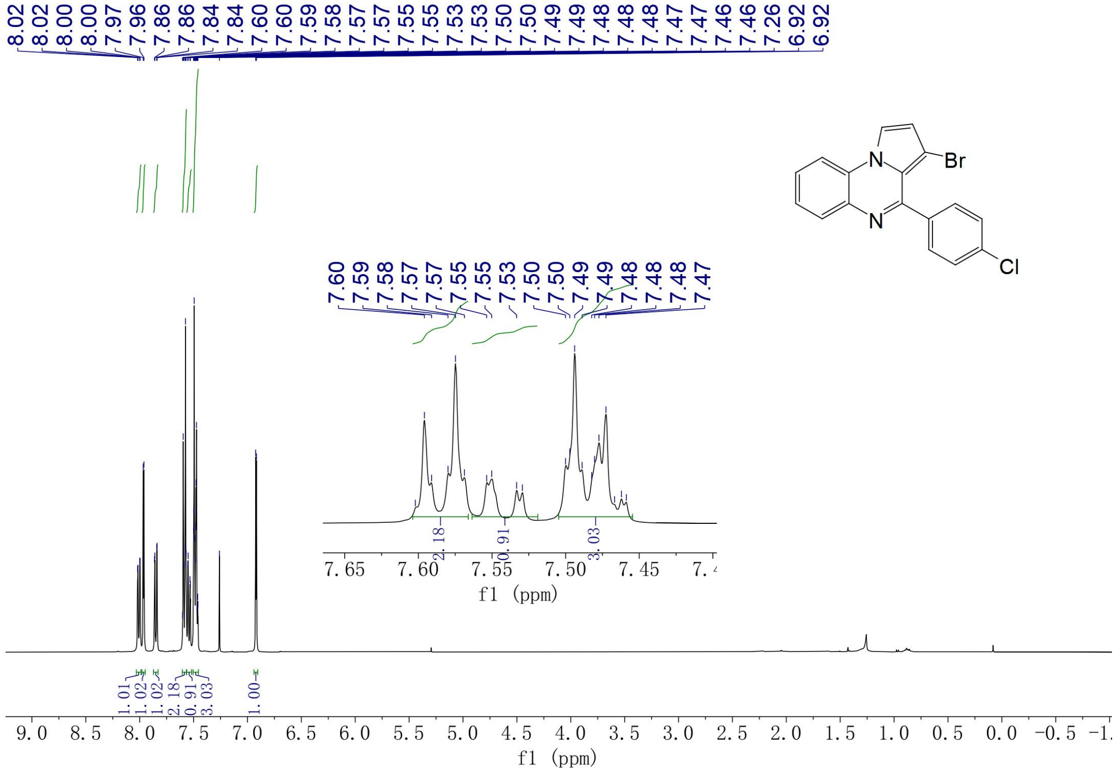

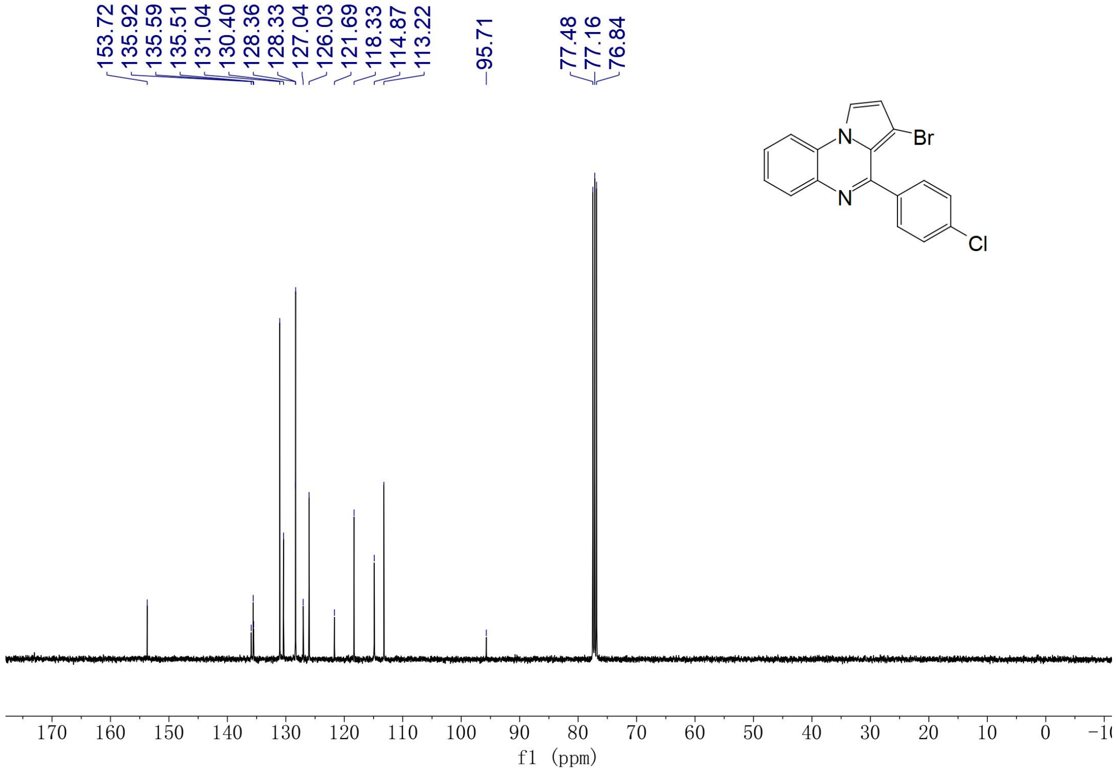

3-bromo-4-(4-bromophenyl)pyrrolo[1,2-*a*]quinoxaline [3f]

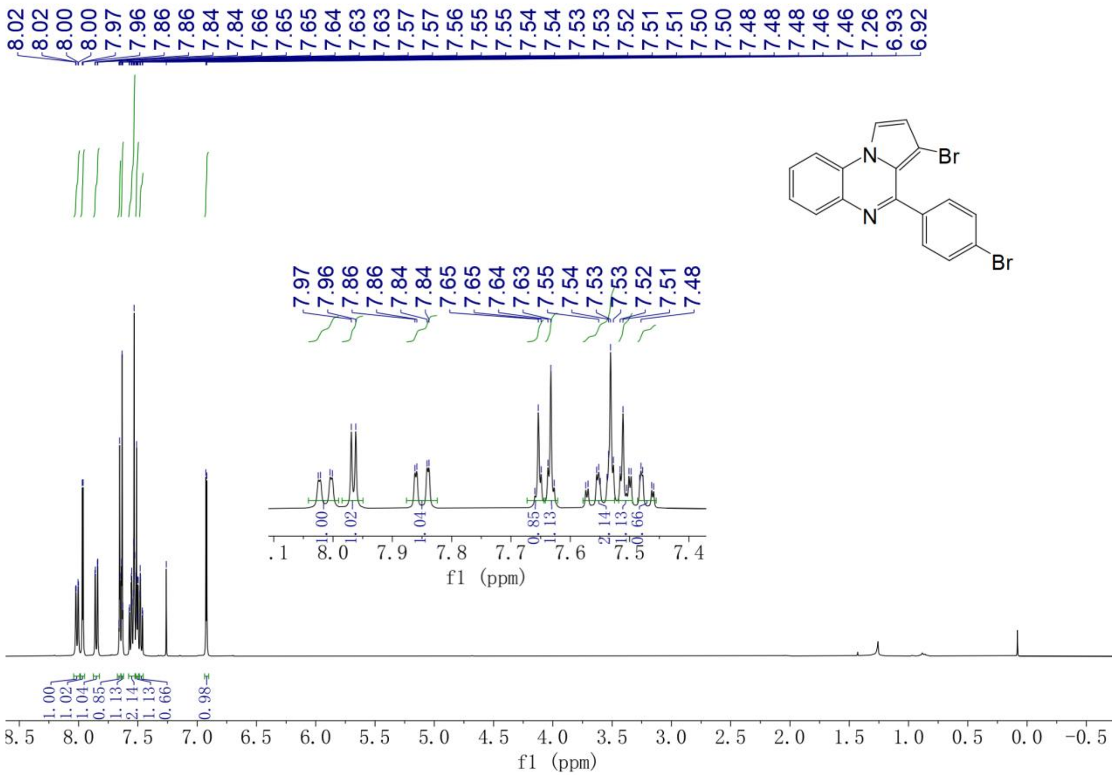

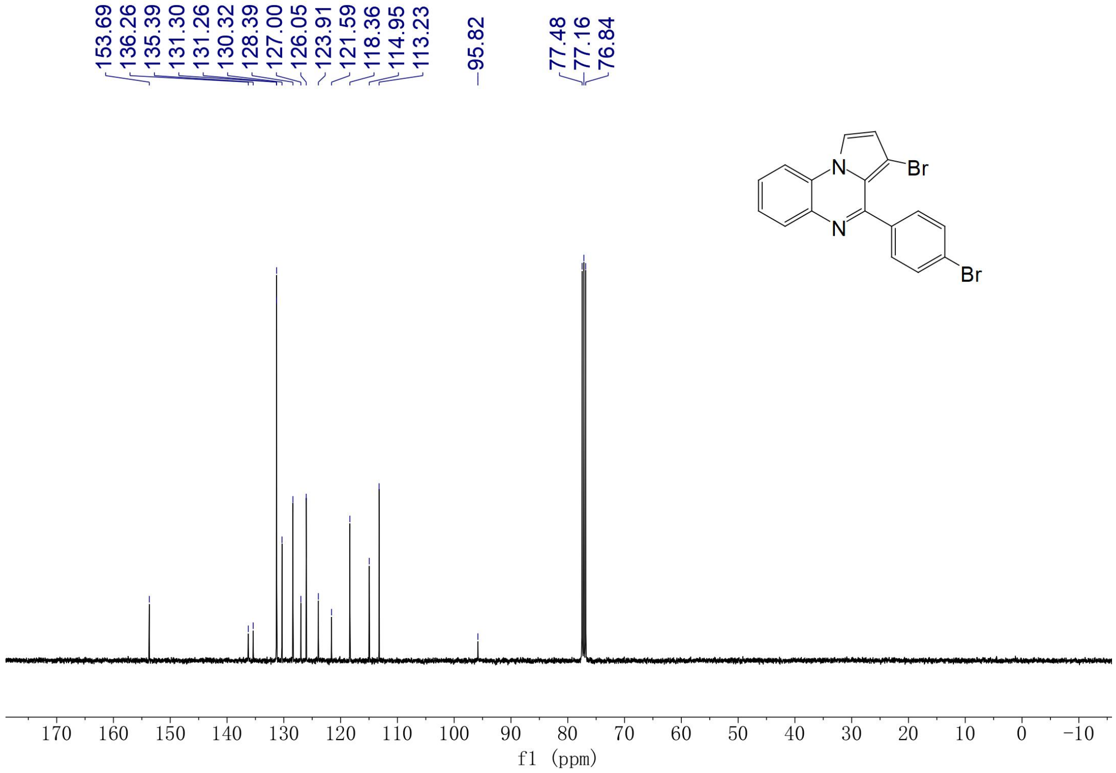

3-bromo-4-(4-fluorophenyl)pyrrolo[1,2-*a*]quinoxaline [3g]

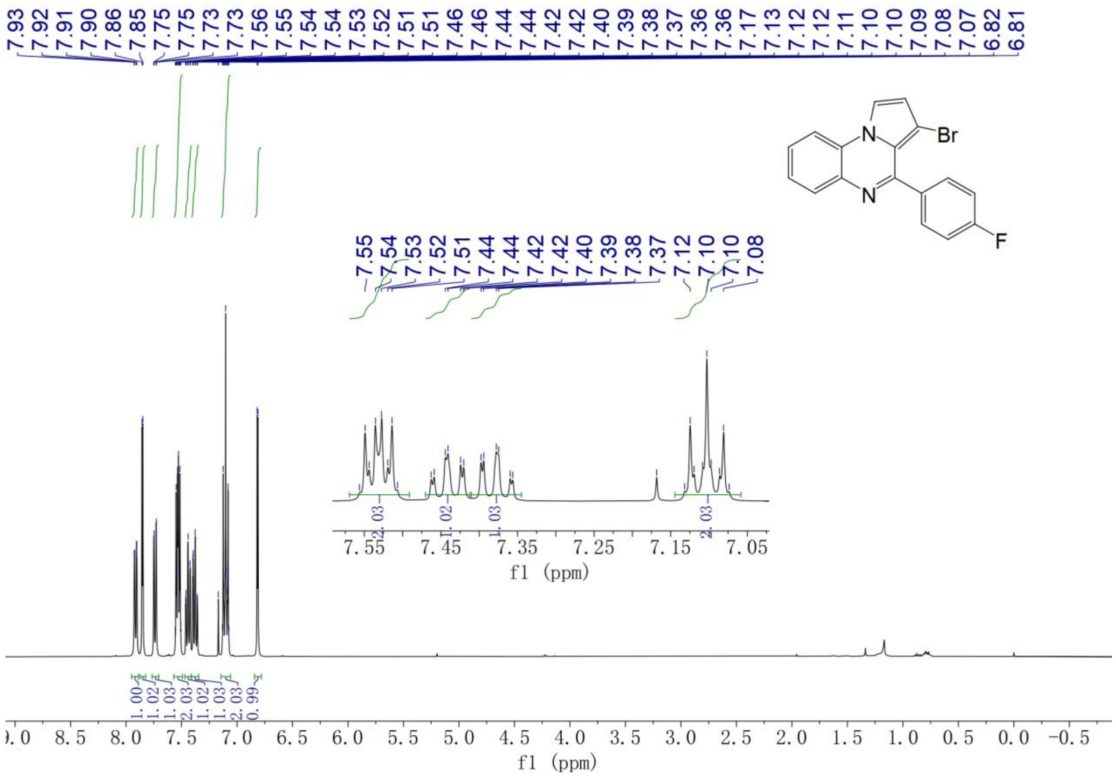

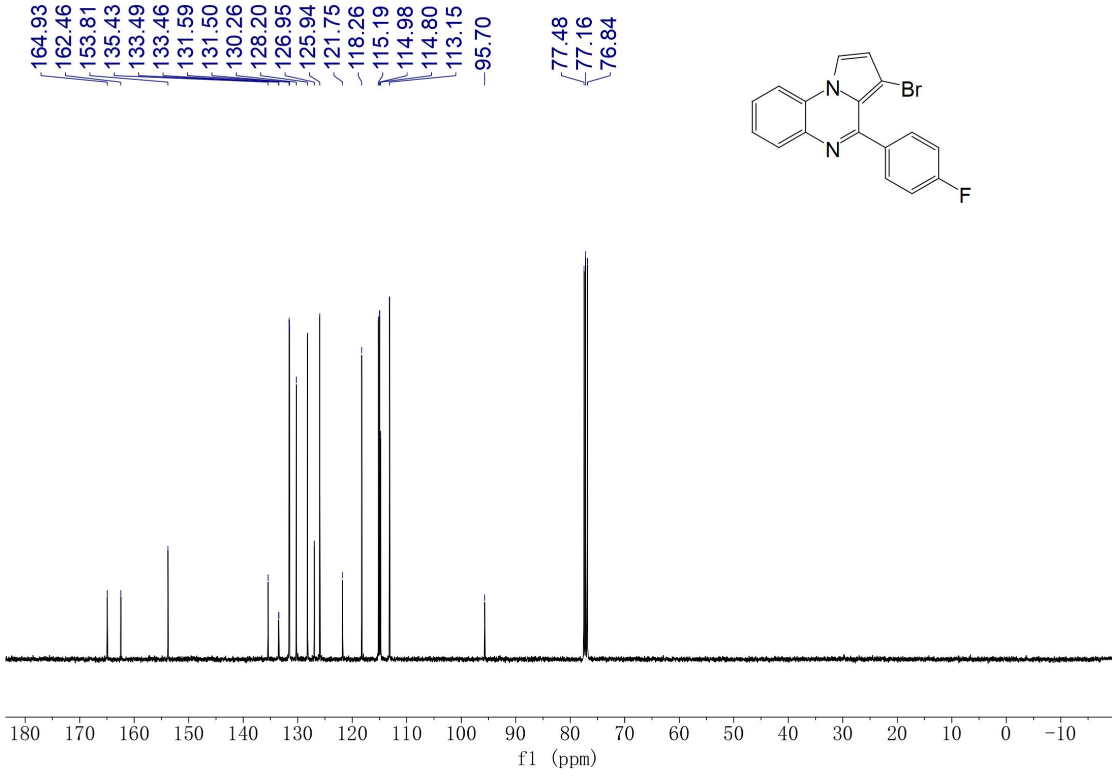

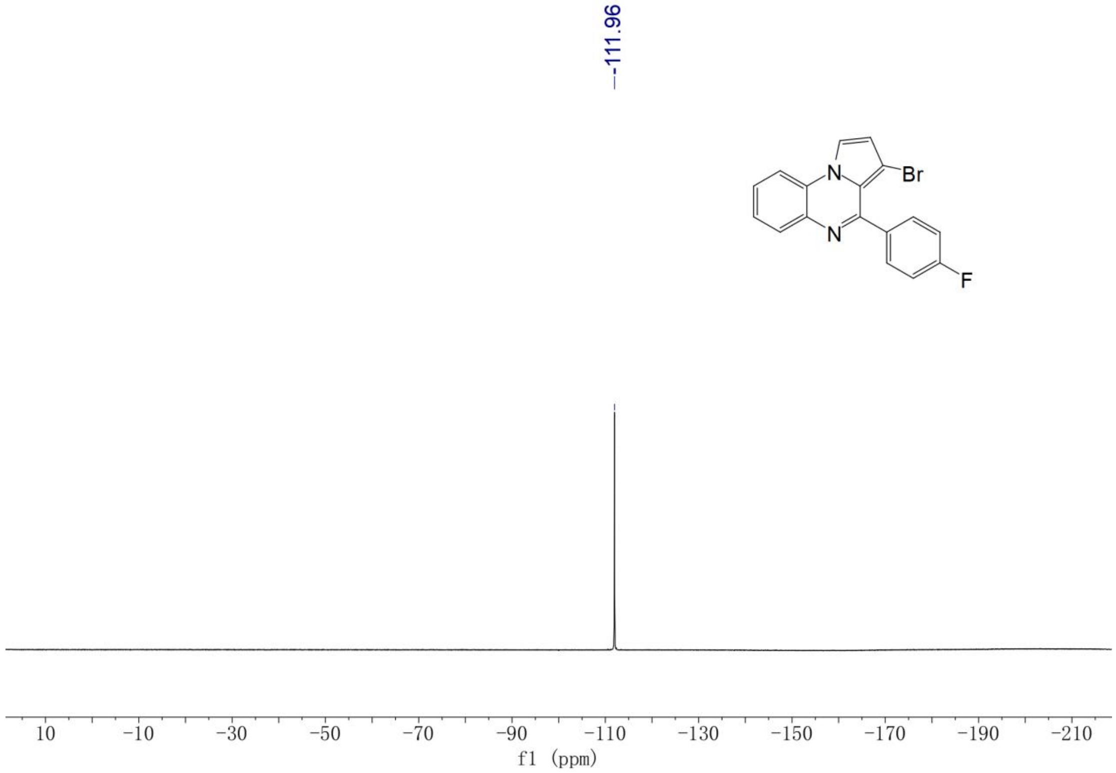

3-bromo-4-(4-nitrophenyl)pyrrolo[1,2-*a*]quinoxaline [3h]

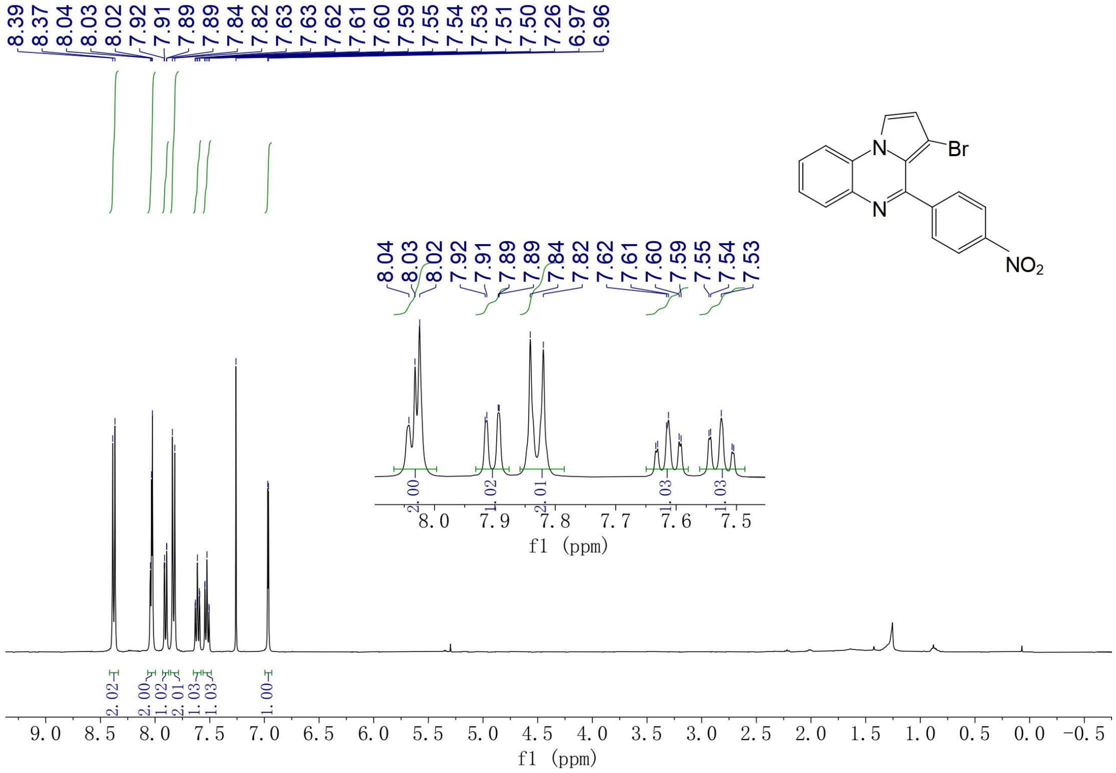

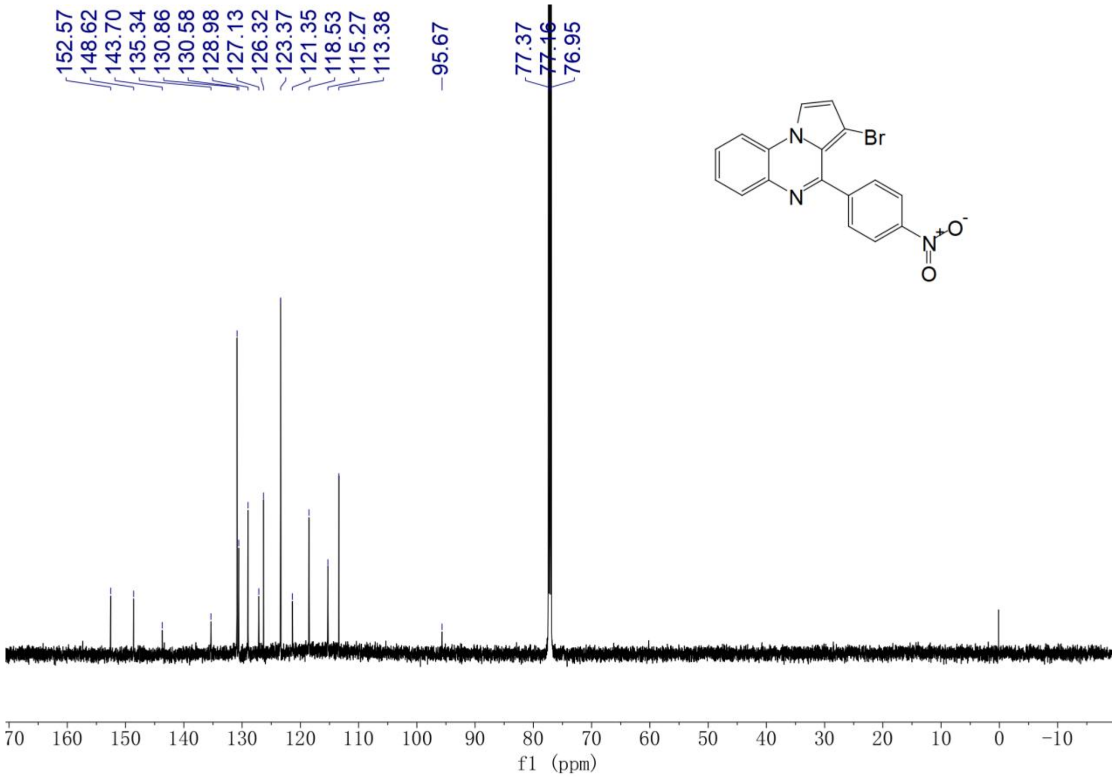

3-bromo-4-(2,4-dichlorophenyl)pyrrolo[1,2-*a*]quinoxaline [3i]

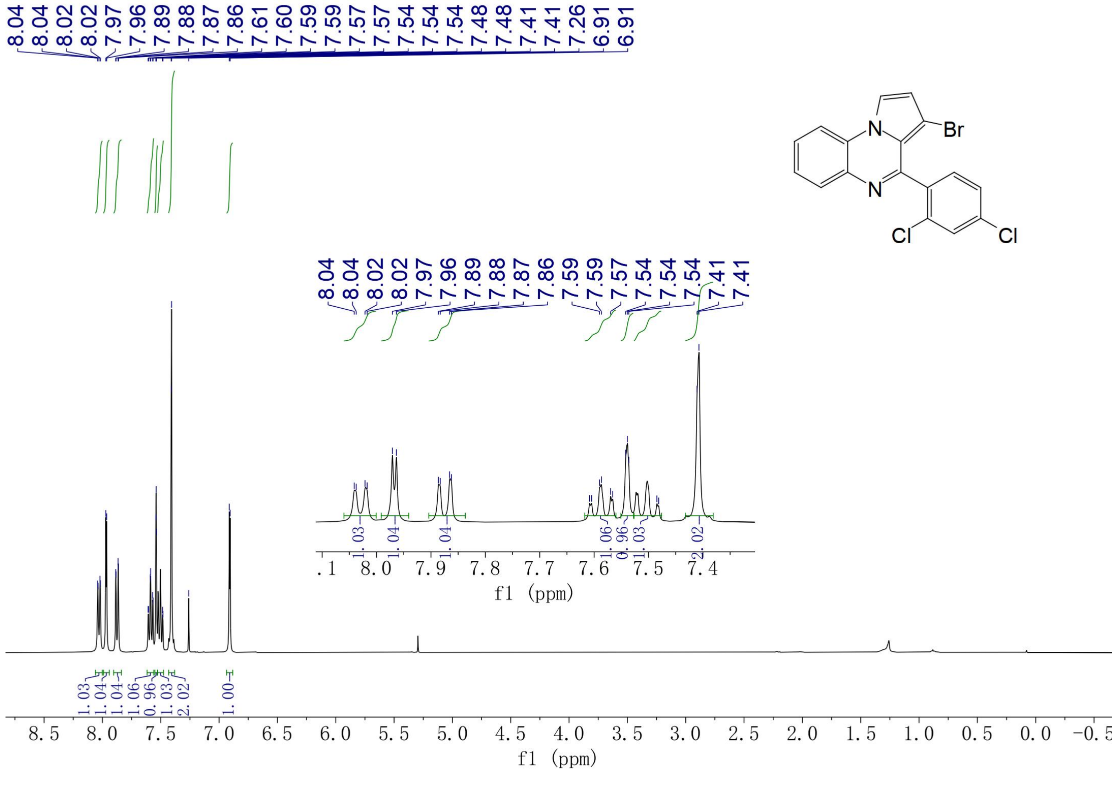

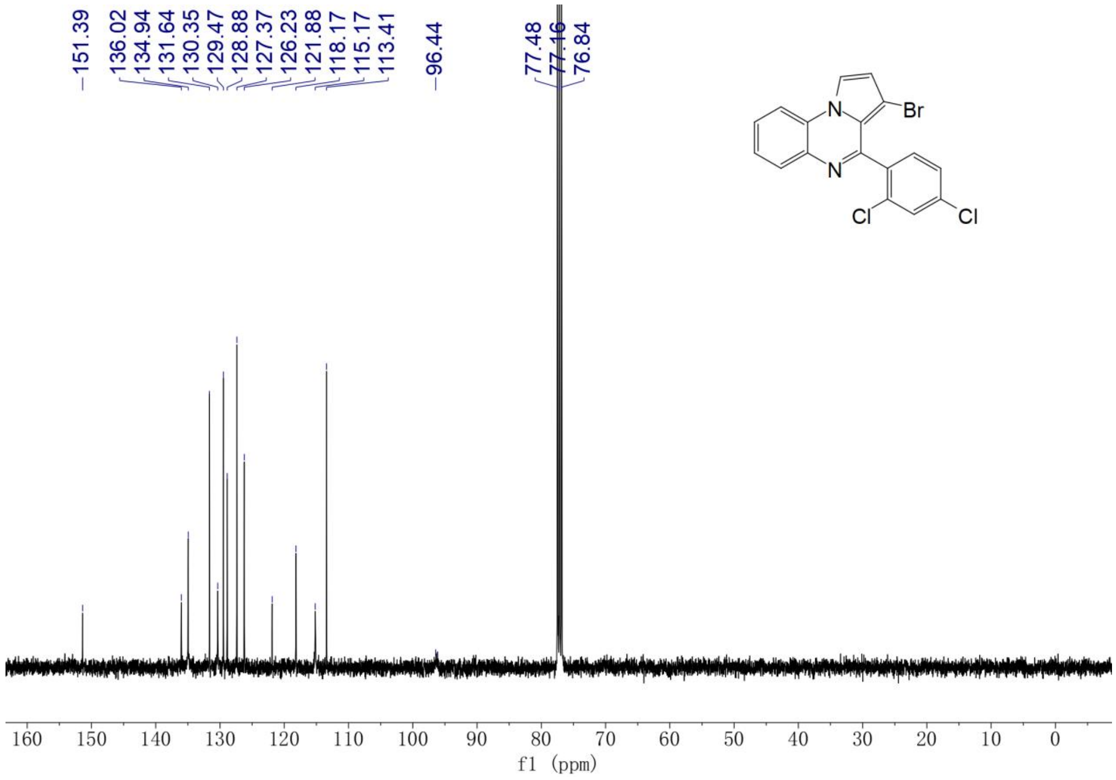

3-bromo-1-phenylpyrrolo[1,2-*a*]quinoxaline [3j]

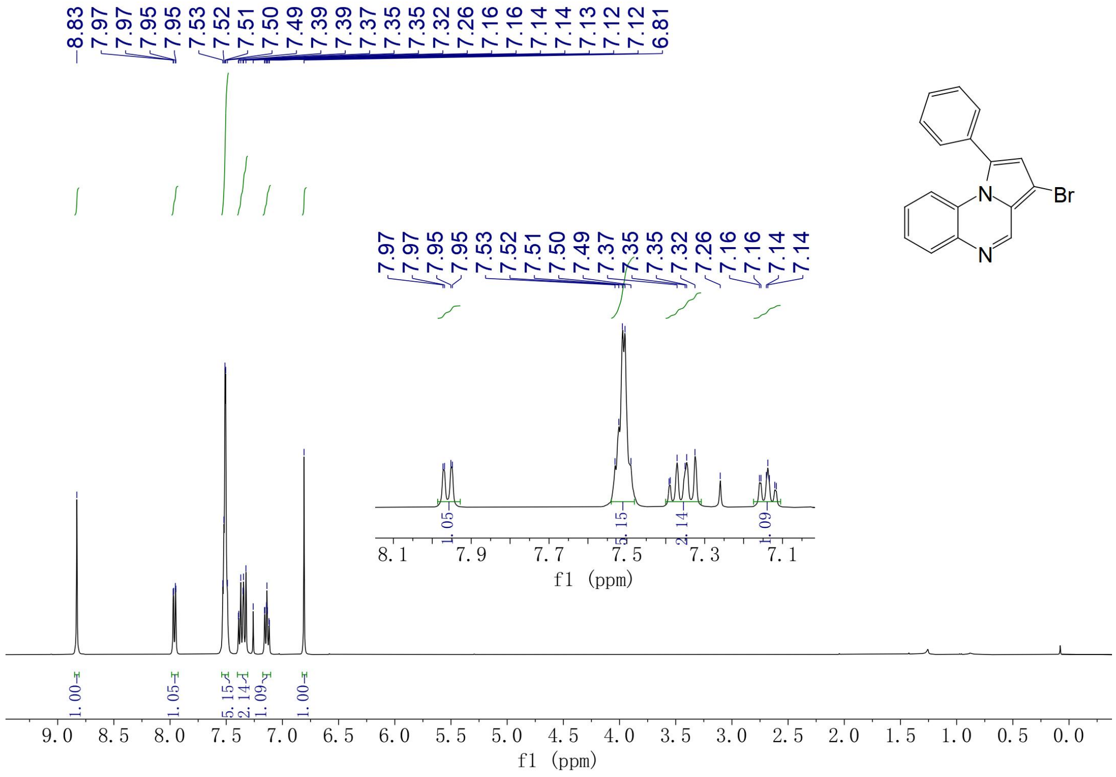

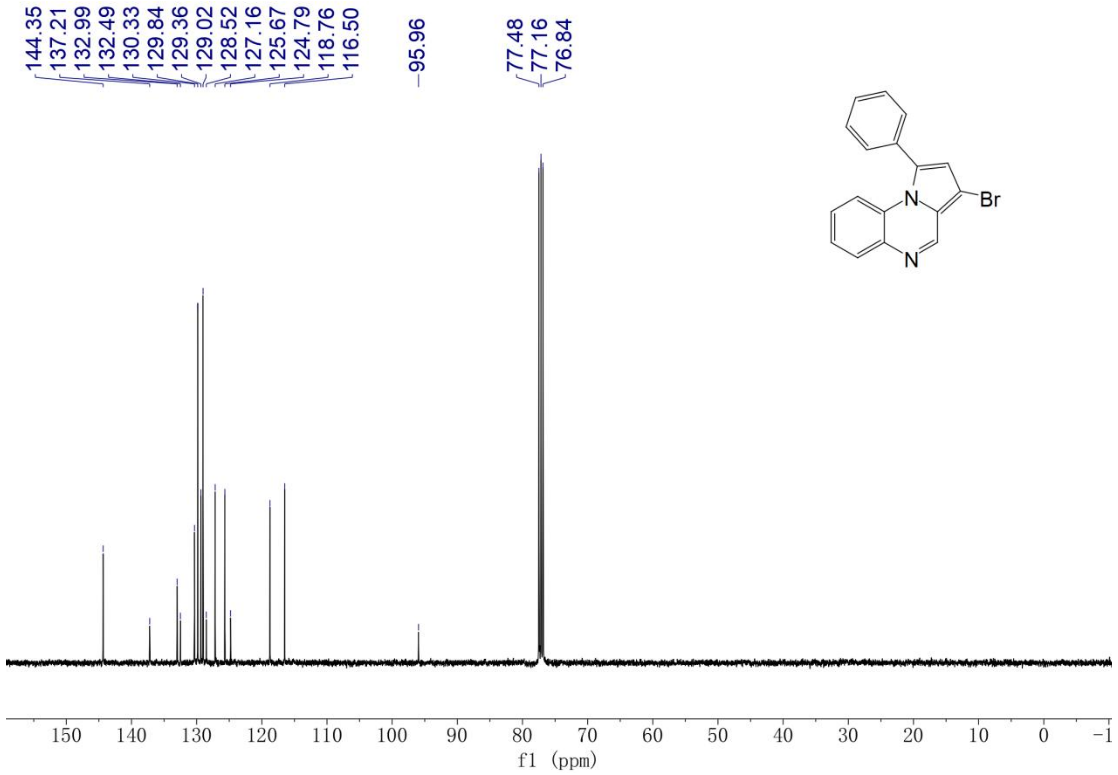

3-bromo-1-(p-tolyl)pyrrolo[1,2-*a*]quinoxaline [3k]

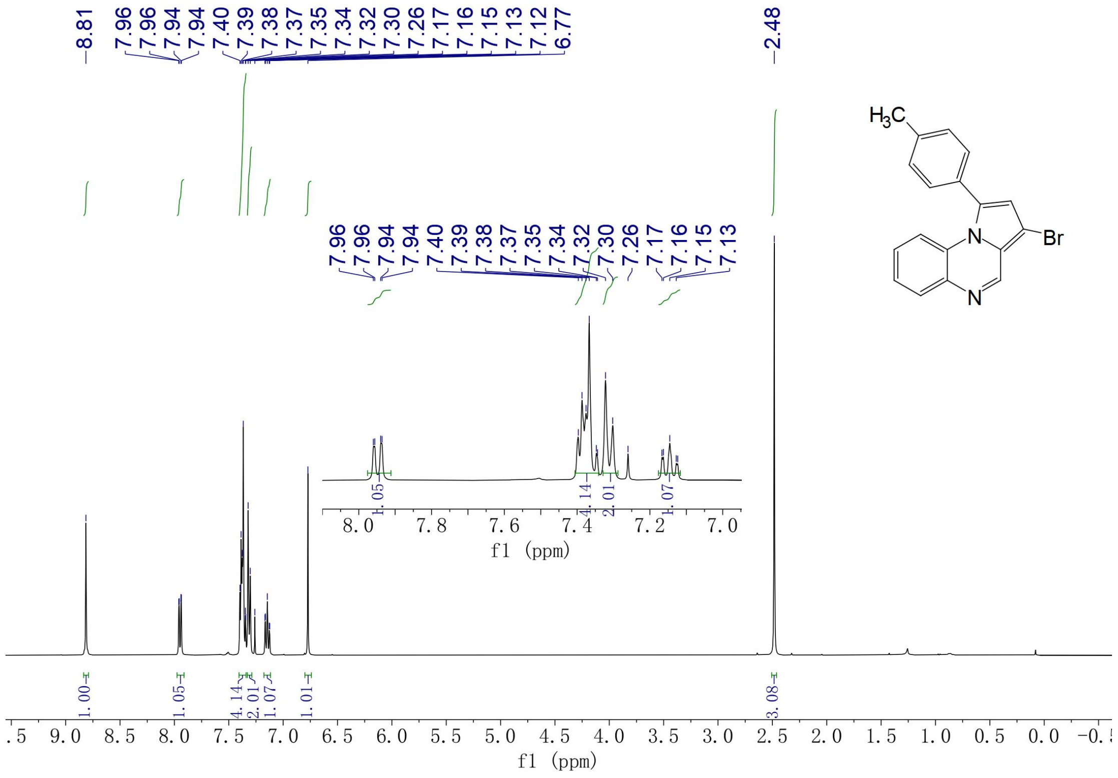

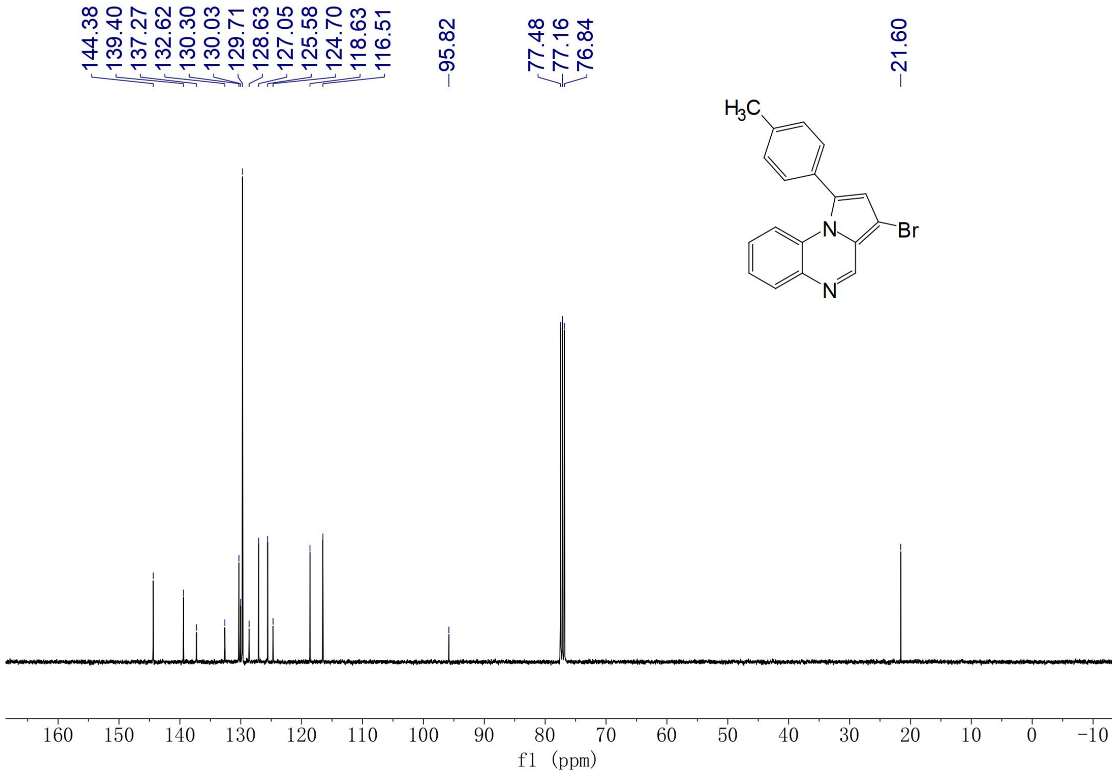

3-bromo-1-(4-methoxyphenyl)pyrrolo[1,2-*a*]quinoxaline [3l]

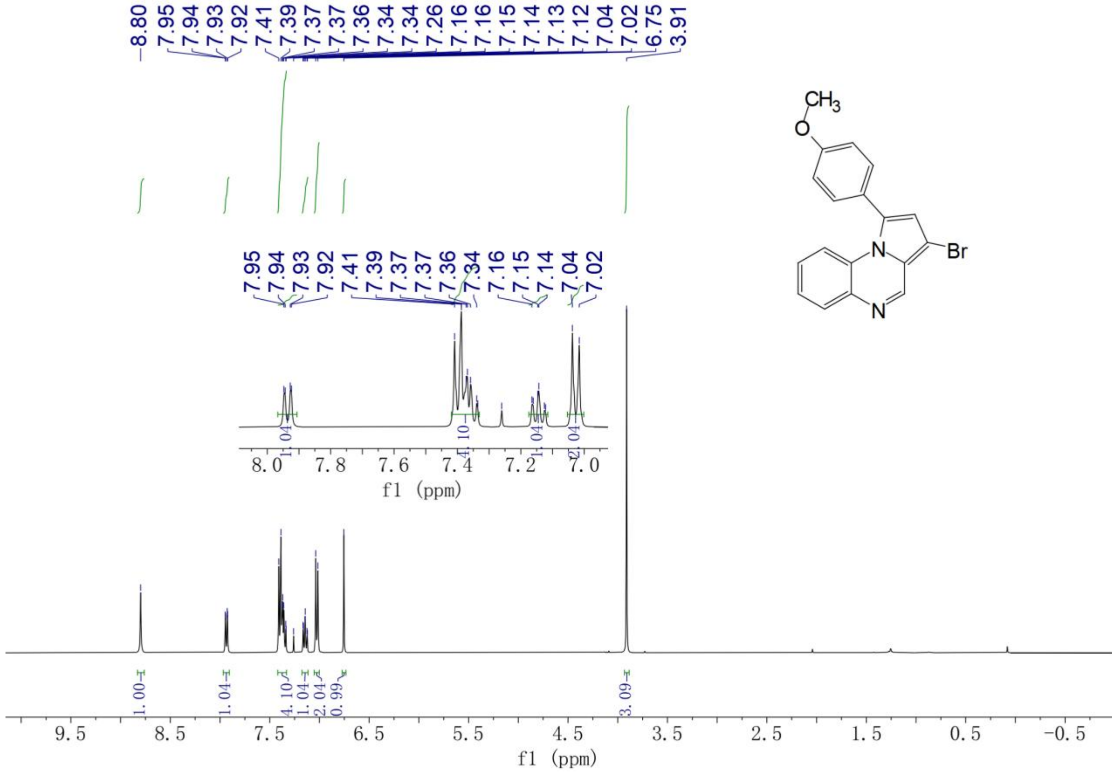

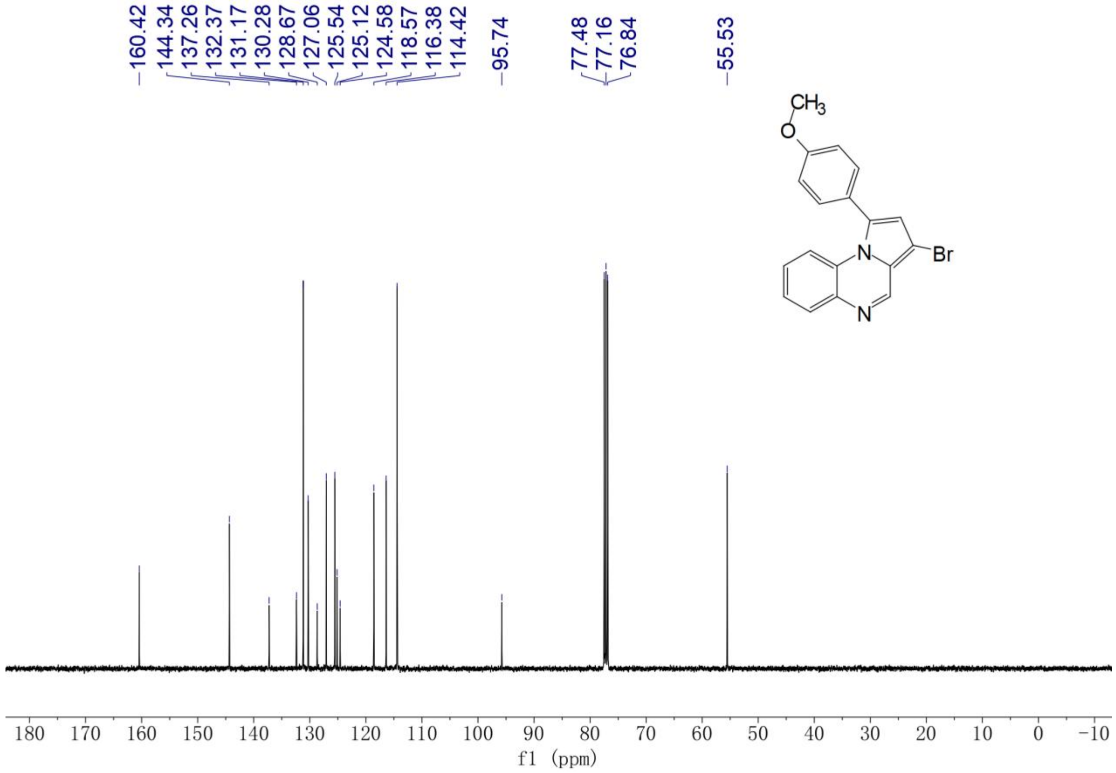

3-bromo-7-methylpyrrolo[1,2-*a*]quinoxaline [3m]

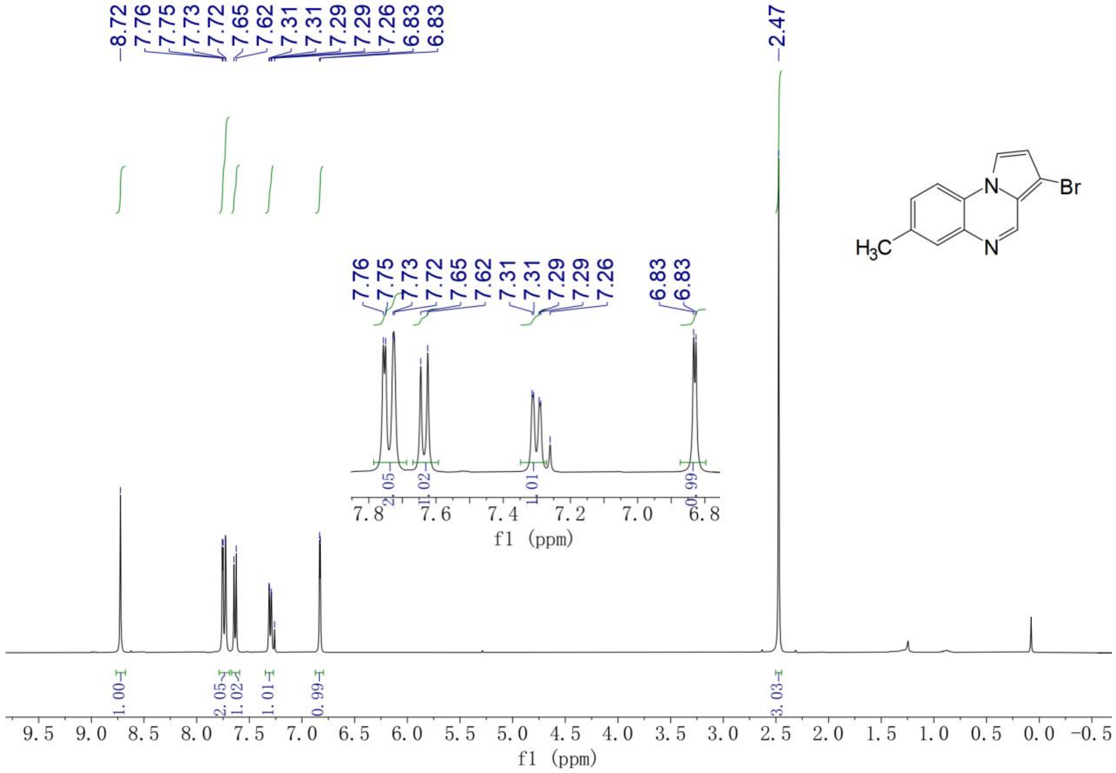

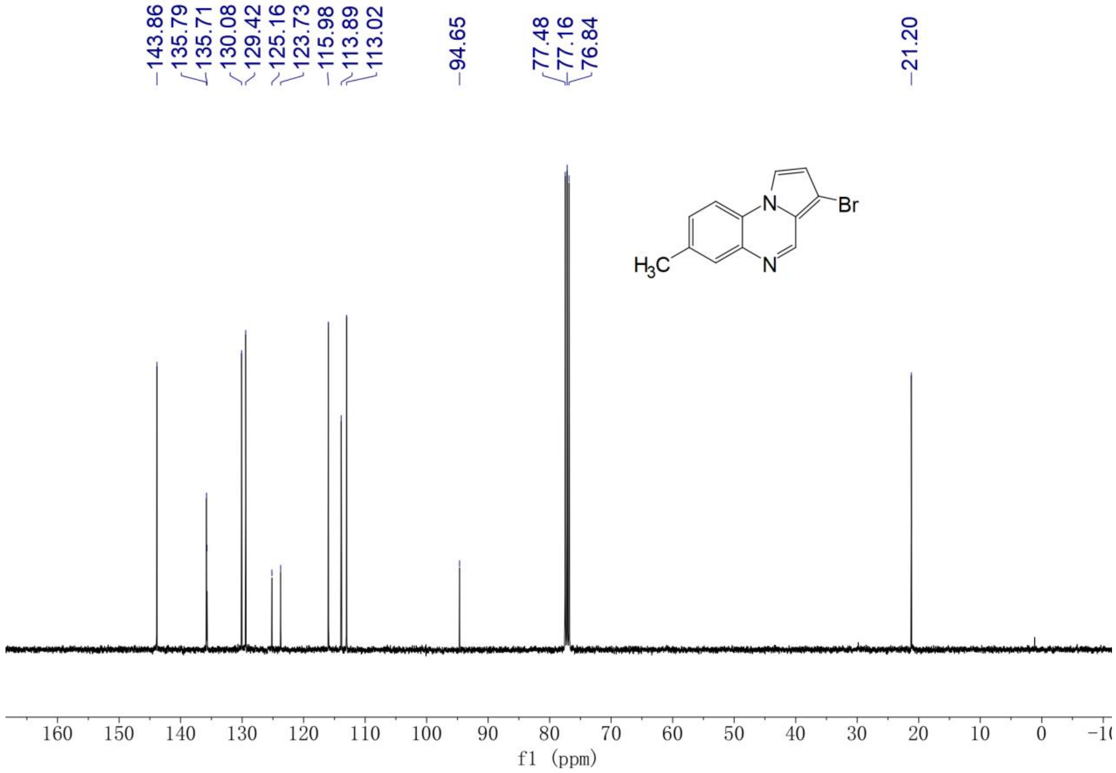

3-bromo-7-chloropyrrolo[1,2-*a*]quinoxaline [3n]

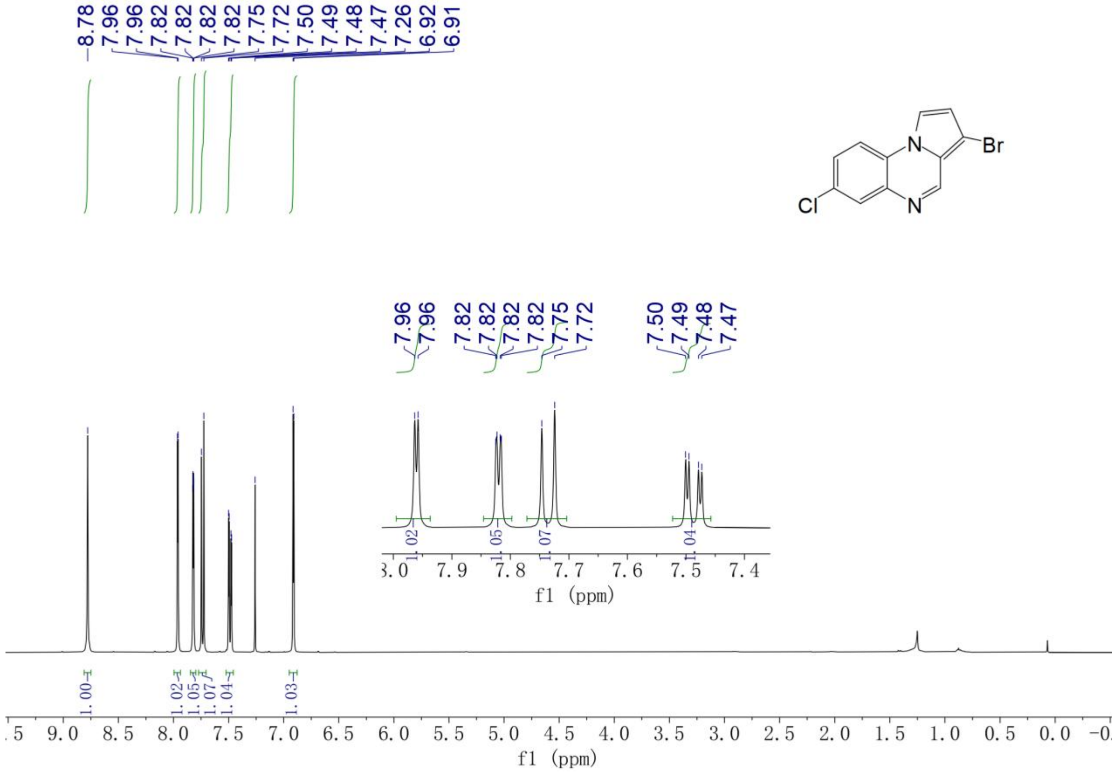

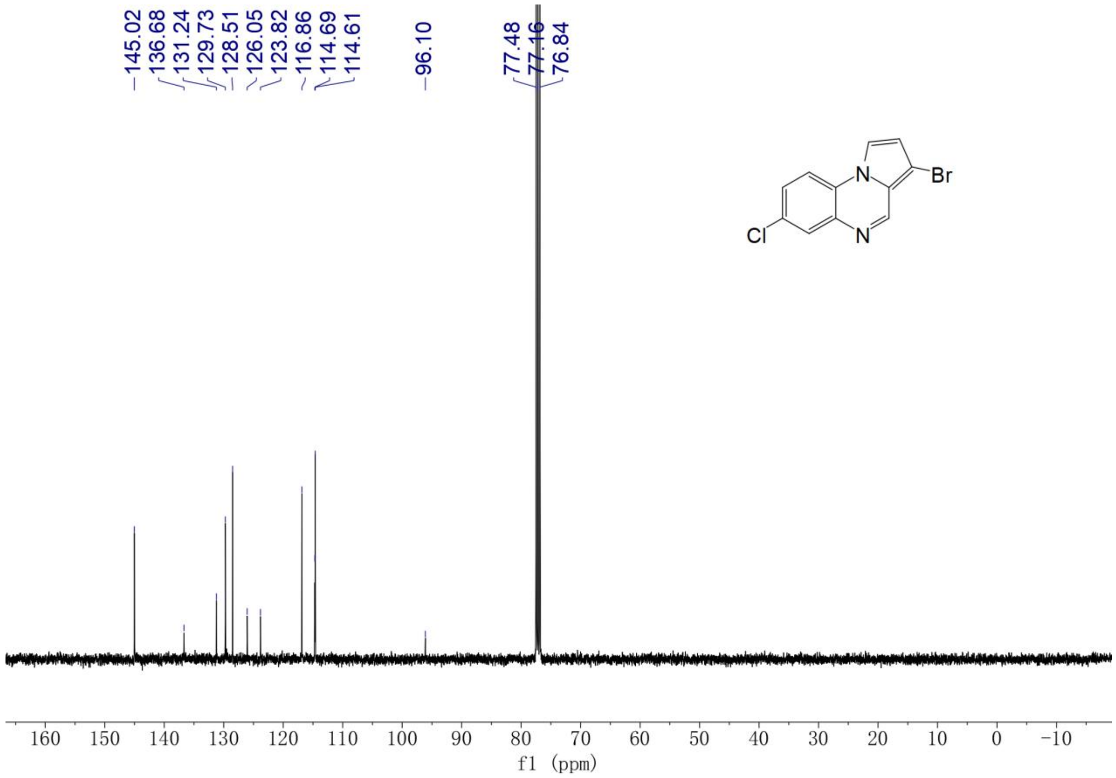

3-bromo-4-(4-methoxyphenyl)-7-methylpyrrolo[1,2-*a*]quinoxaline [3o]

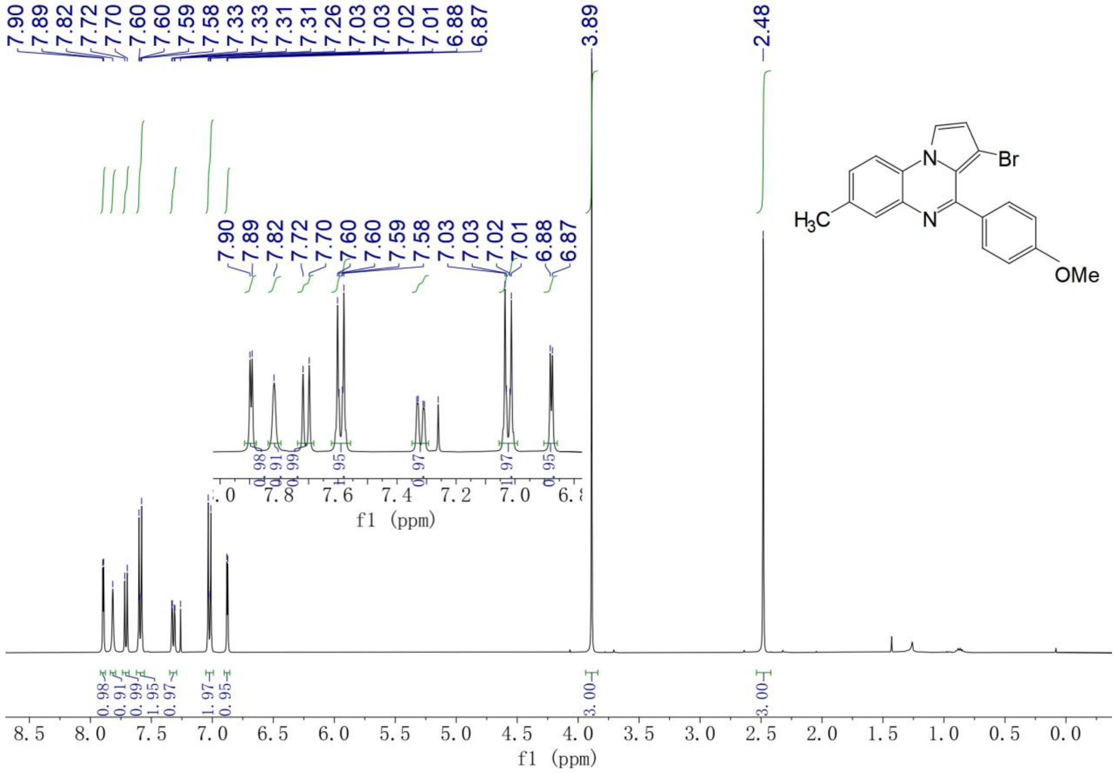

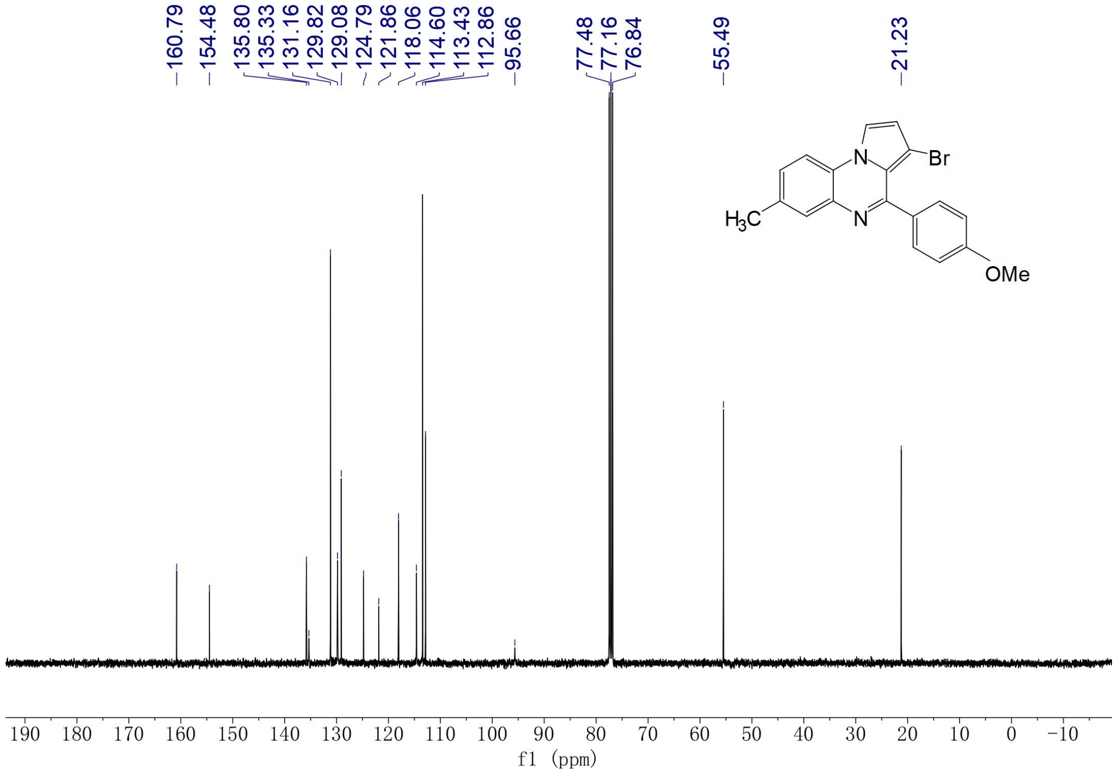

3-bromo-4-(4-bromophenyl)-7-methylpyrrolo[1,2-*a*]quinoxaline [3p]

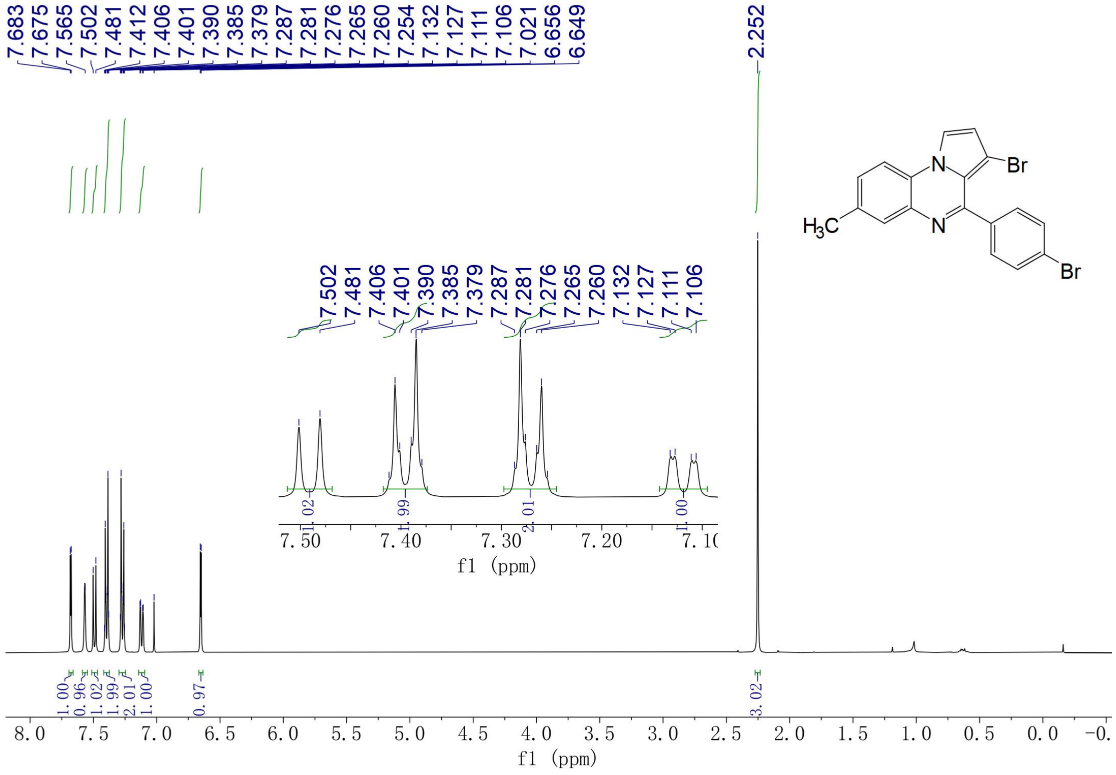

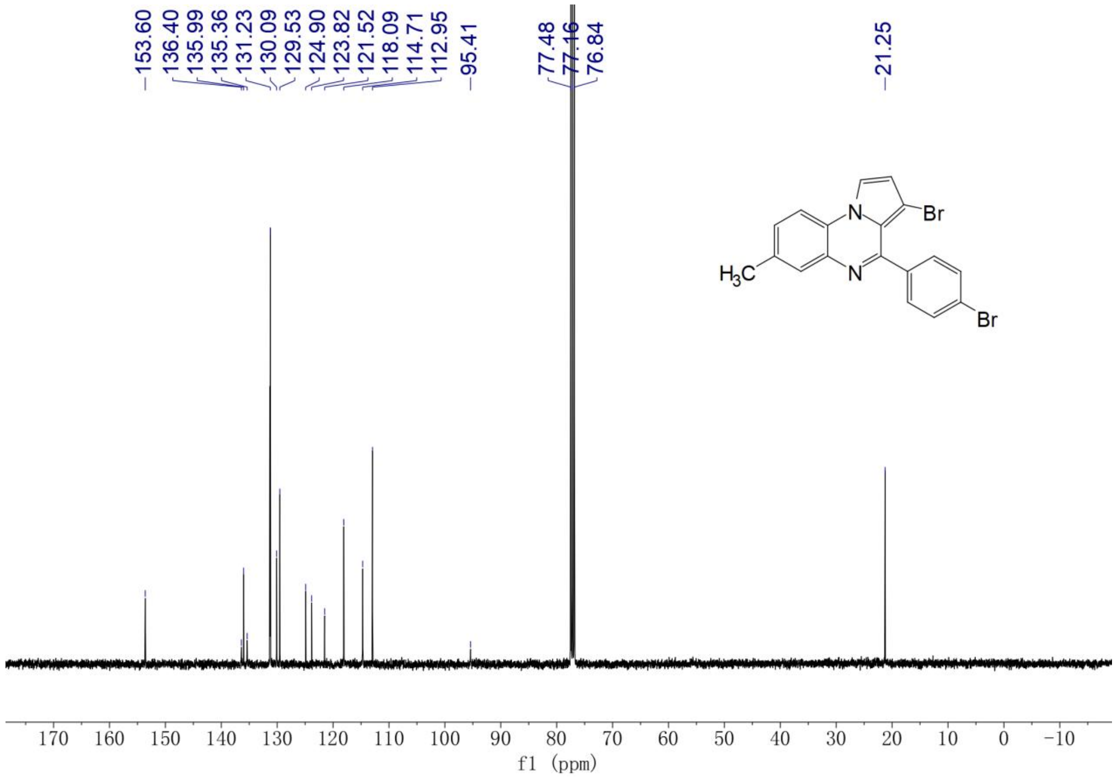

3-bromo-8-methylpyrrolo[1,2-*a*]quinoxaline [3q]

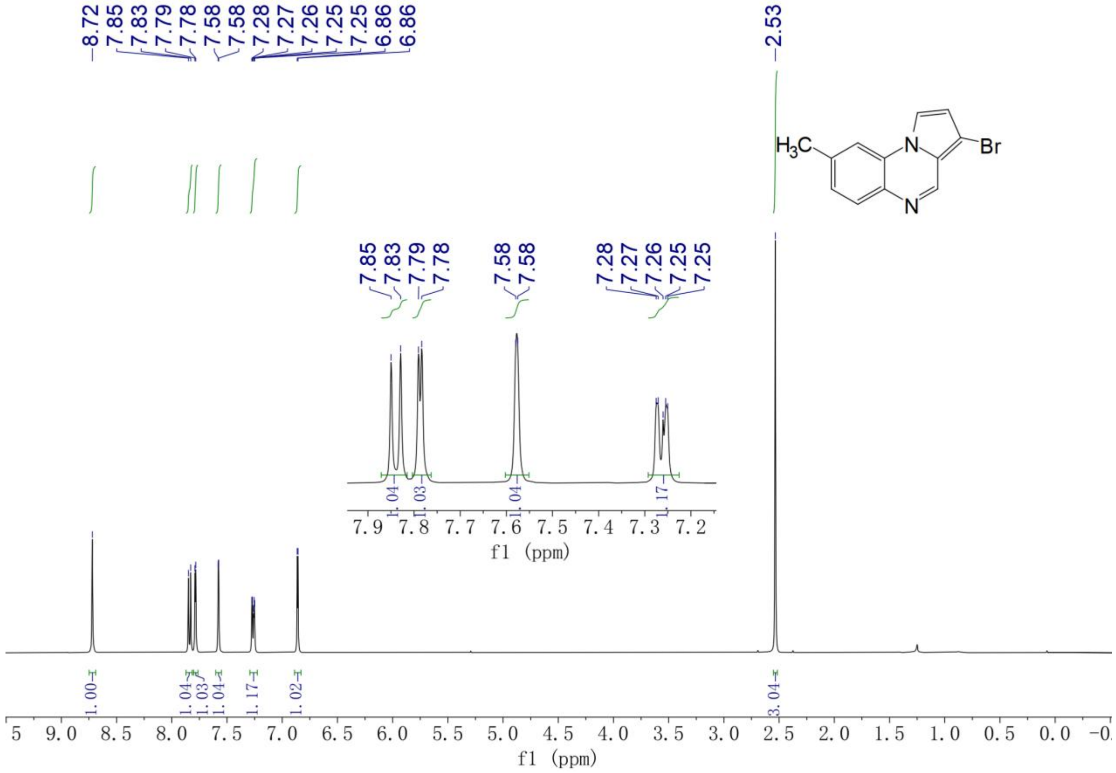

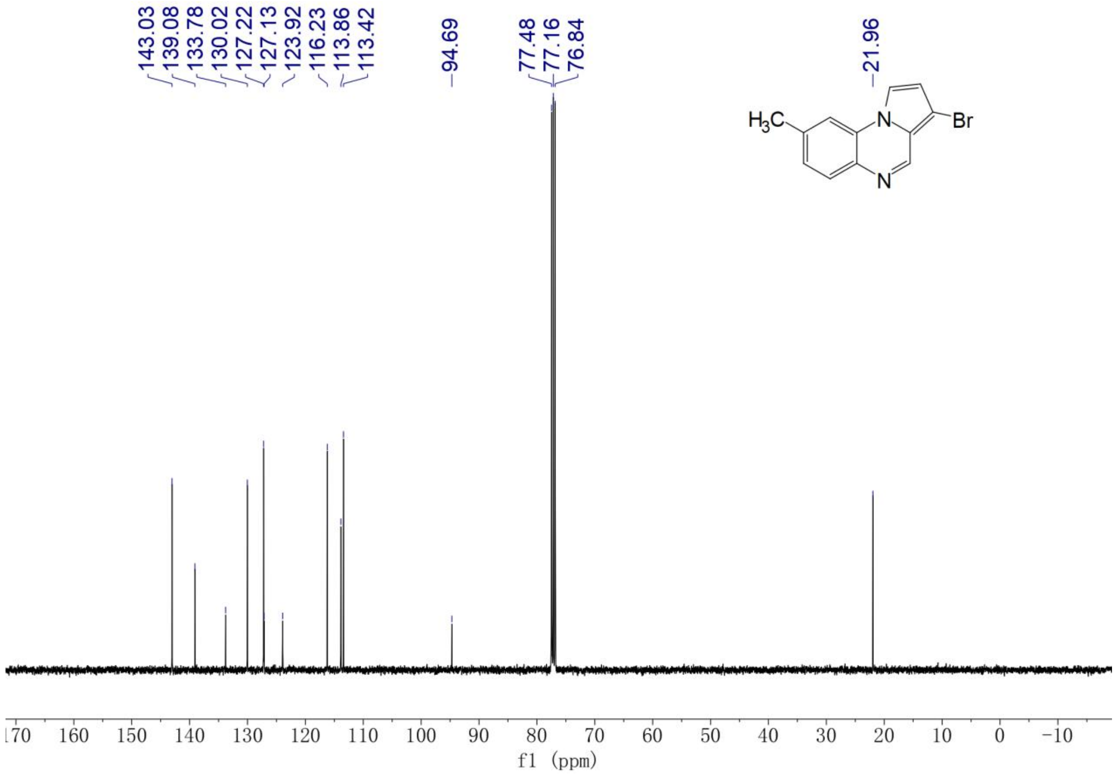

3-bromo-8-chloropyrrolo[1,2-*a*]quinoxaline [3r]

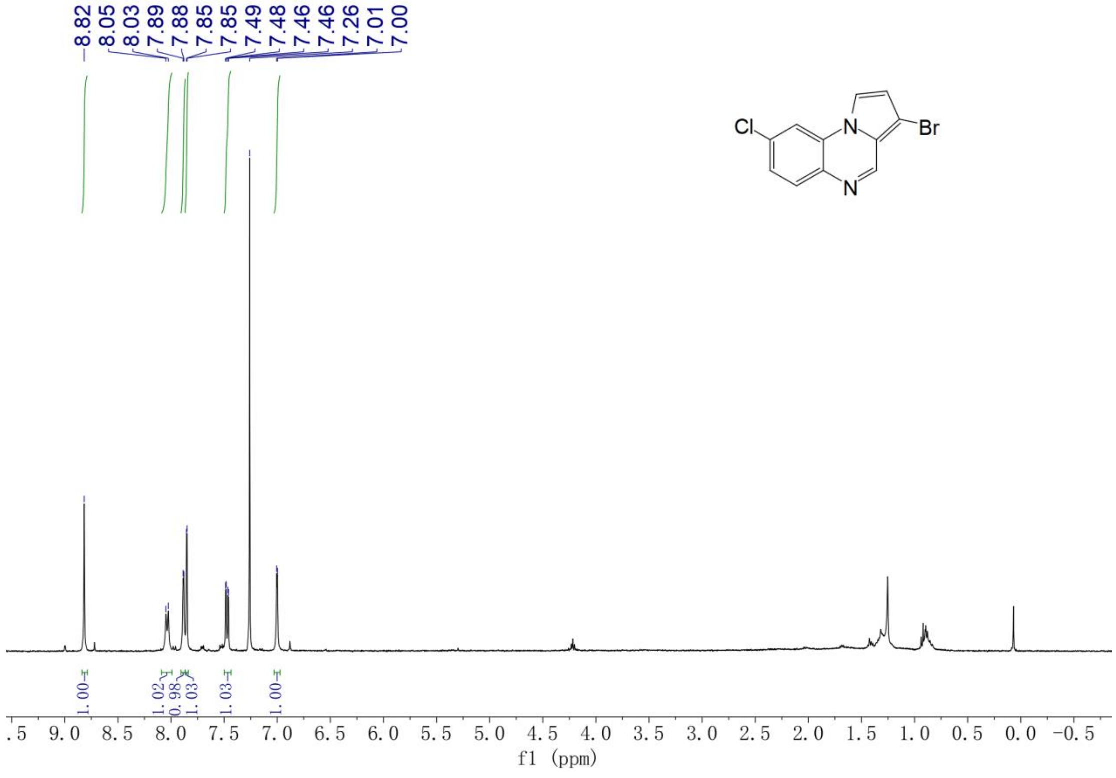

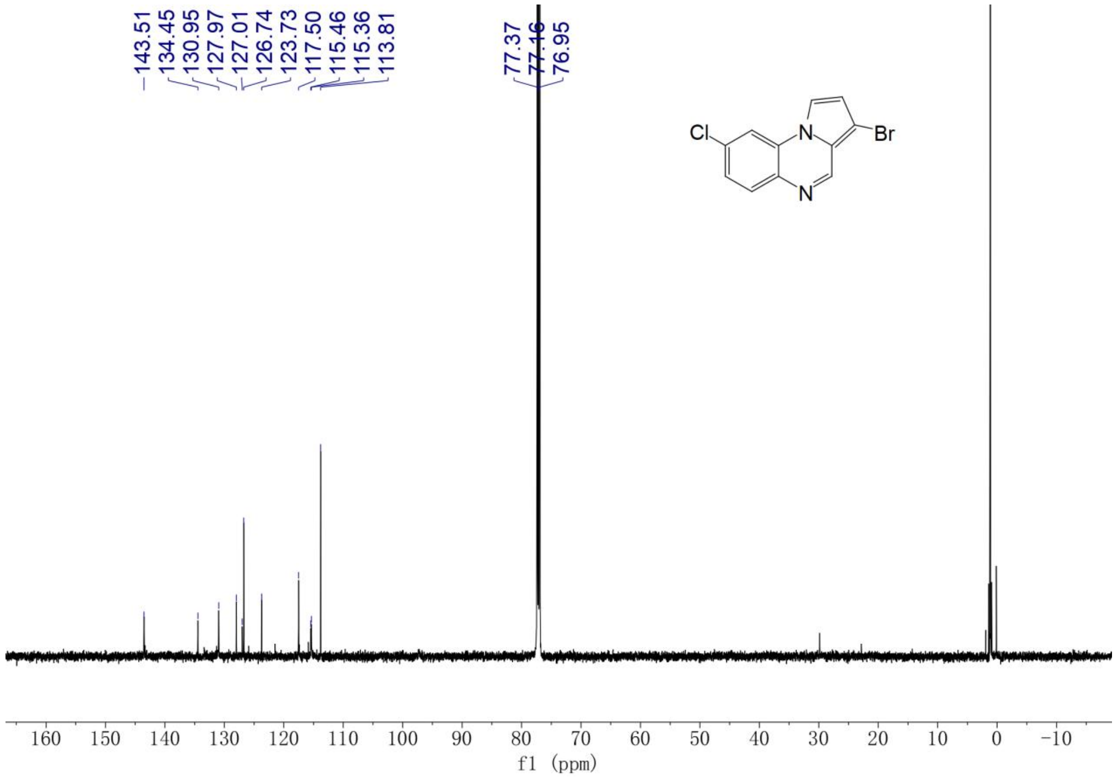

3-bromo-8-fluoropyrrolo[1,2-*a*]quinoxaline [3s]

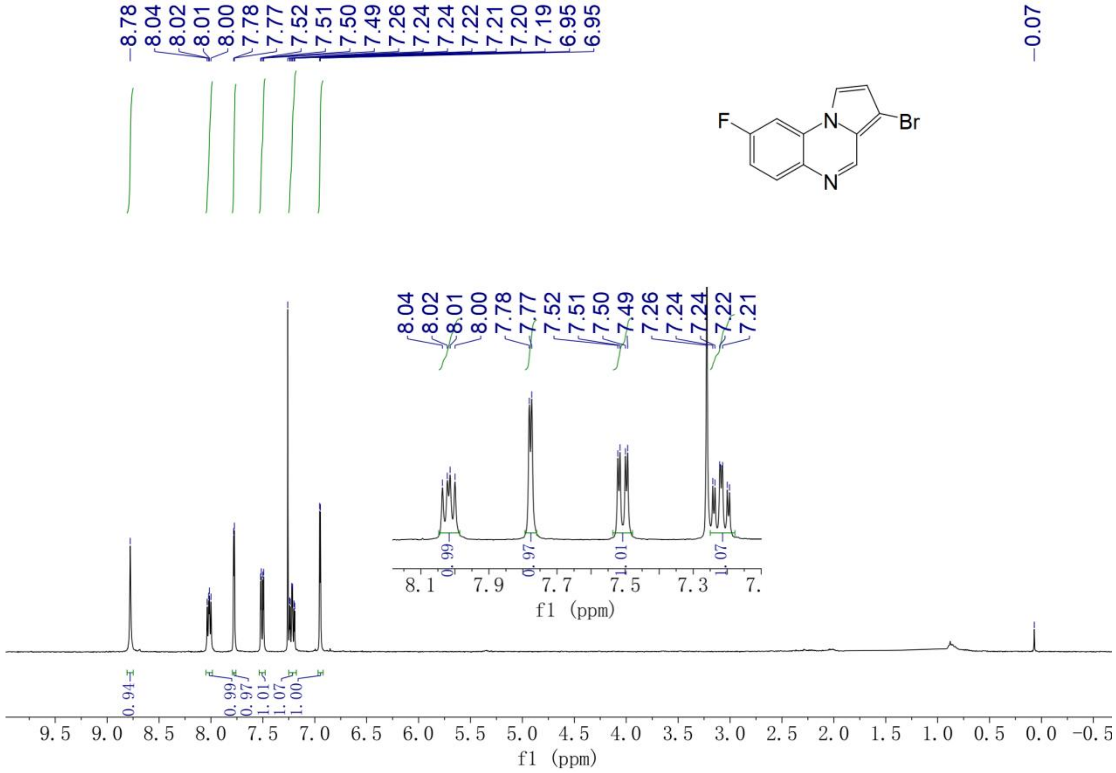

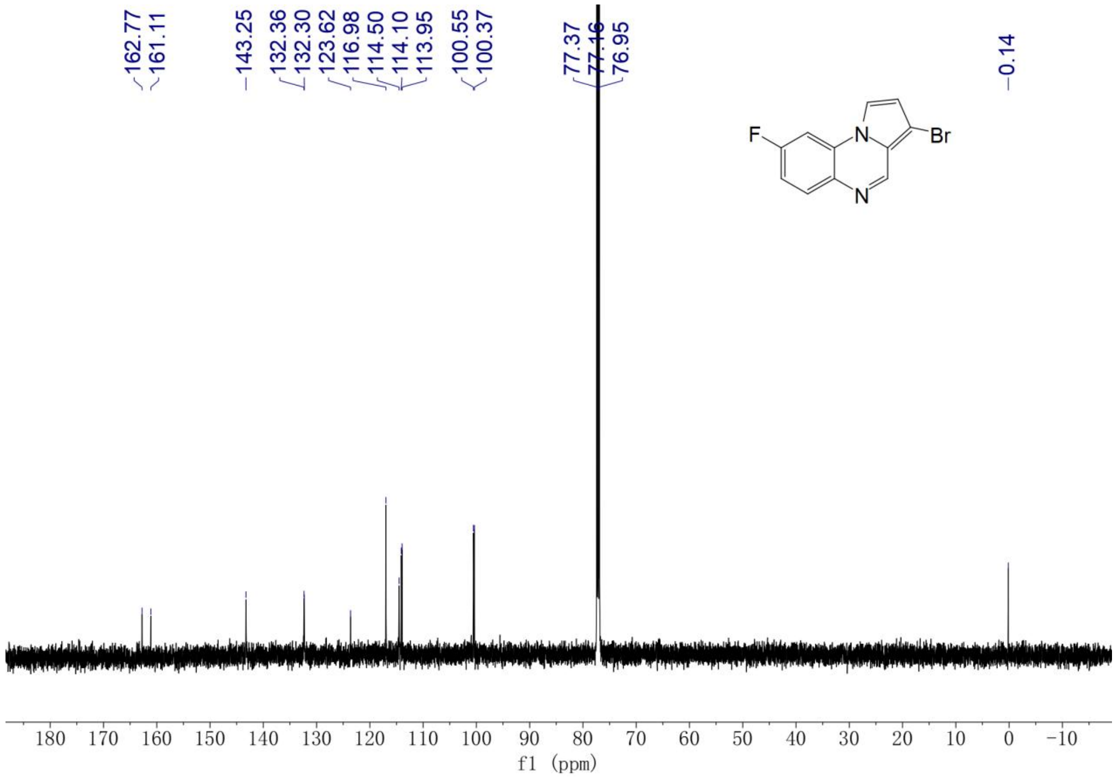
162.77
161.11
143.25
132.36
132.30
123.62
116.98
114.50
114.10
113.95
100.55
100.37
77.37
77.16
76.95
0.14
F
N
Br
N
180 170 160 150 140 130 120 110 100 90 80 70 60 50 40 30 20 10 0 −10
f1 (ppm)

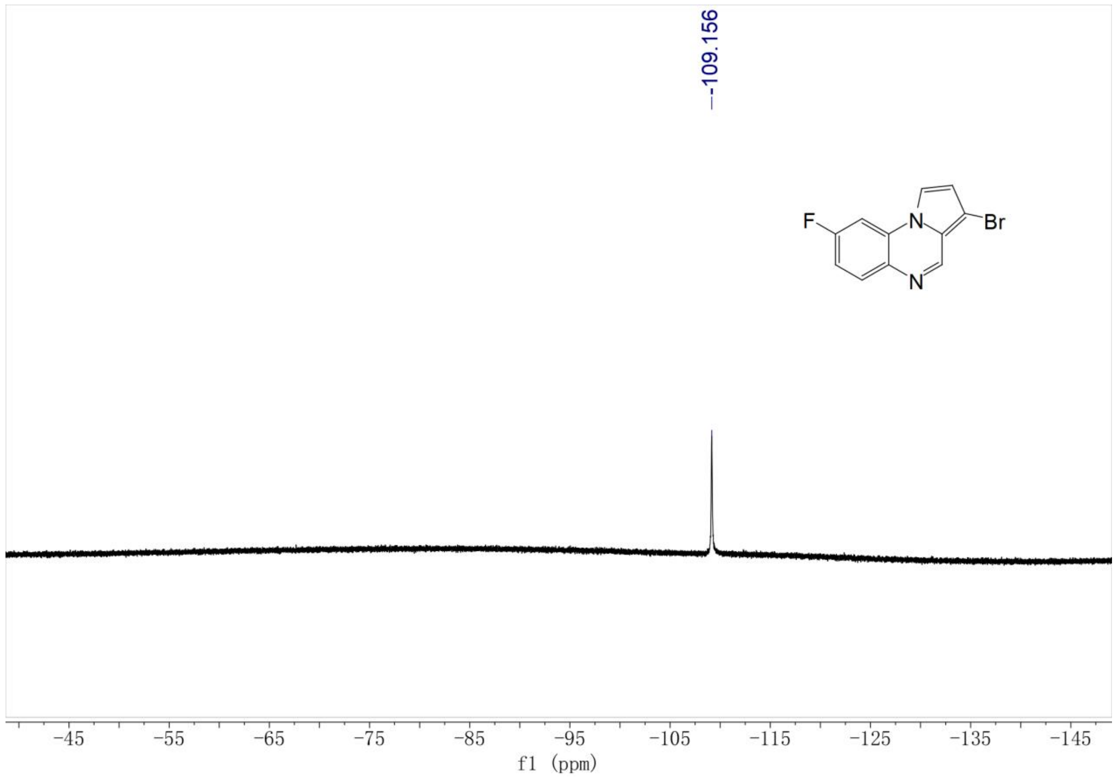
-109.156
F
N
Br
N
−45 −55 −65 −75 −85 −95 −105 −115 −125 −135 −145
f1 (ppm)

1-bromo-3-phenylpyrrolo[1,2-*a*]quinoxaline [3t]

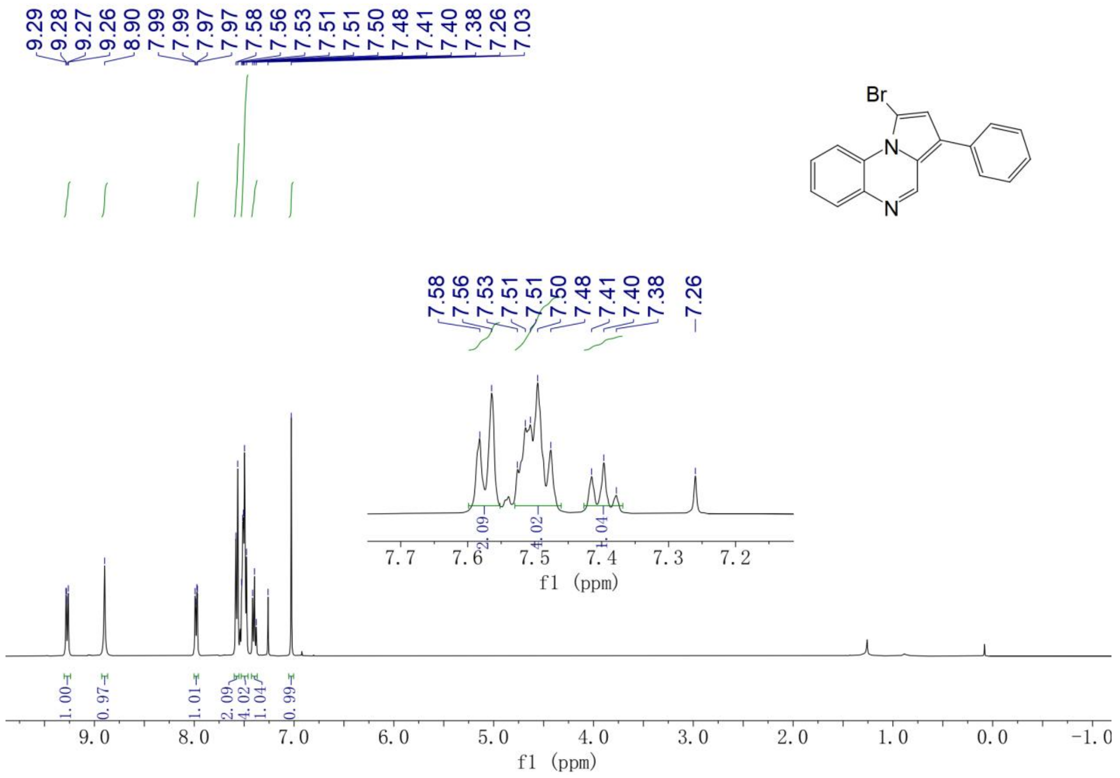

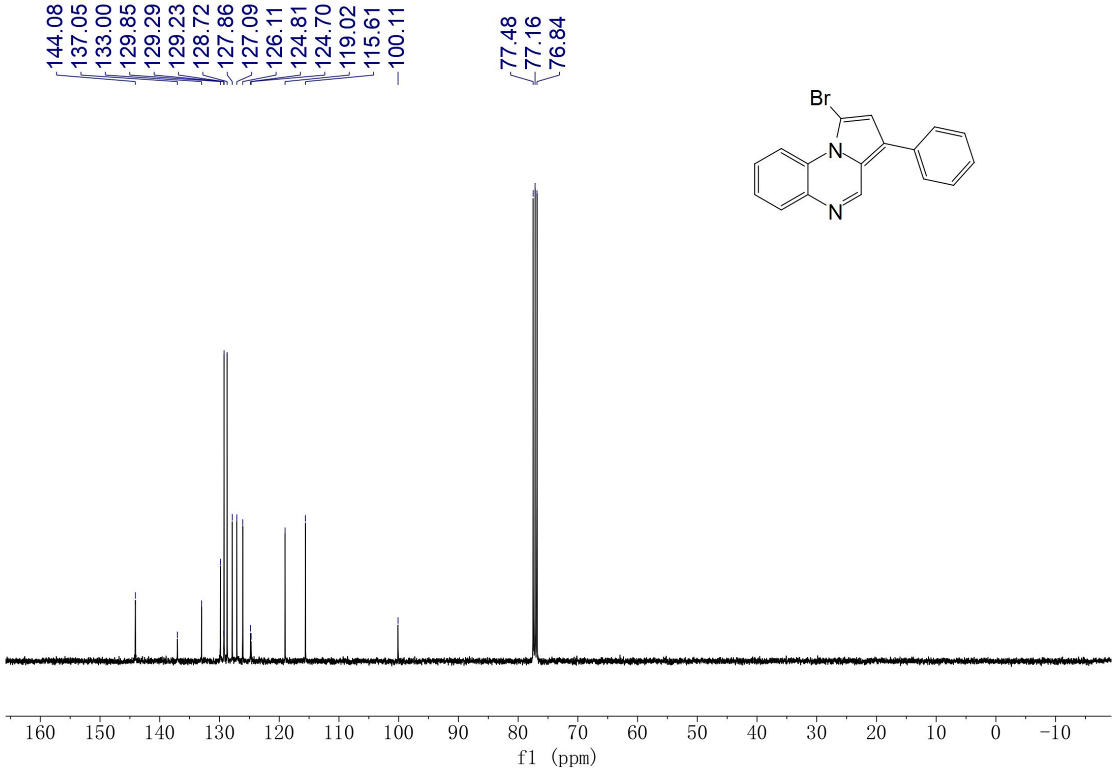

1-bromo-3-(p-tolyl)pyrrolo[1,2-*a*]quinoxaline [3u]

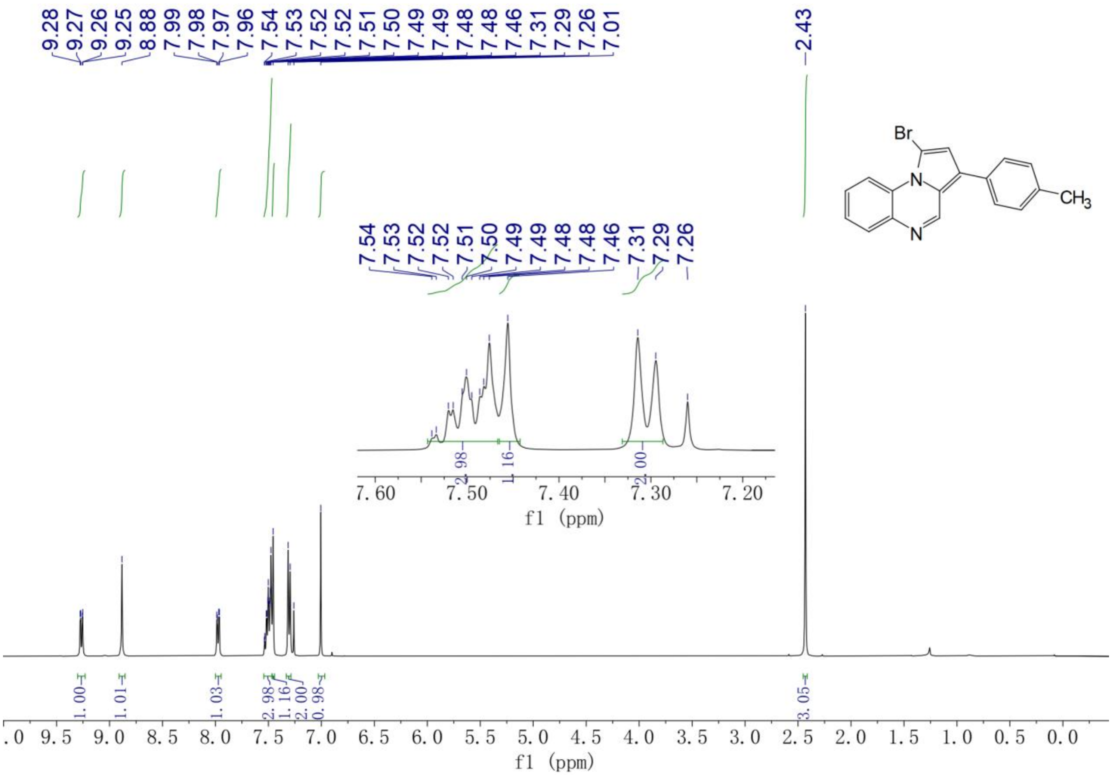

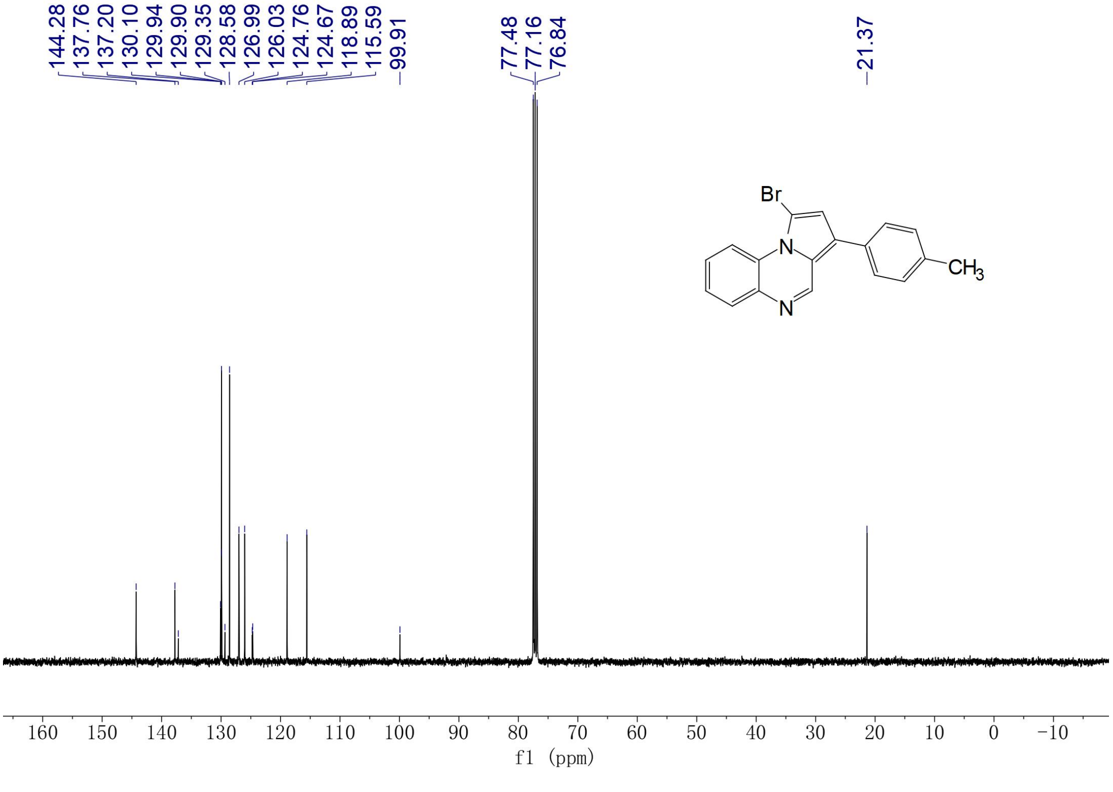

4-(1-bromopyrrolo[1,2-*a*]quinoxalin-3-yl)benzonitrile [3v]

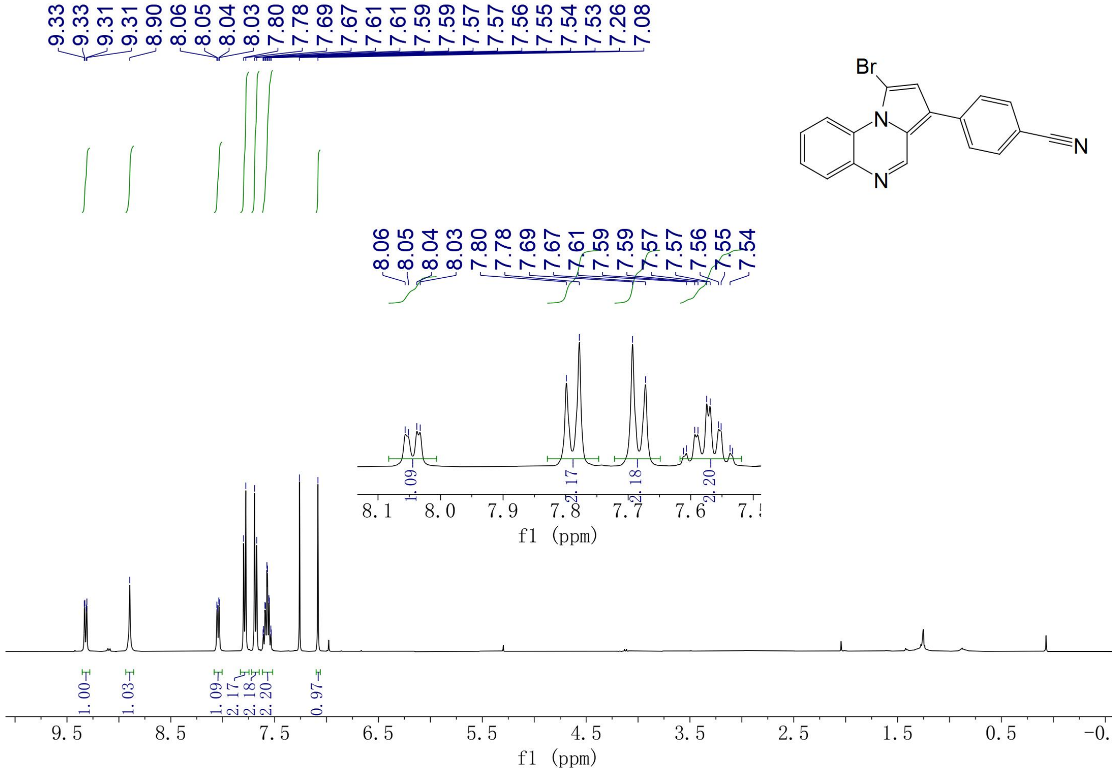

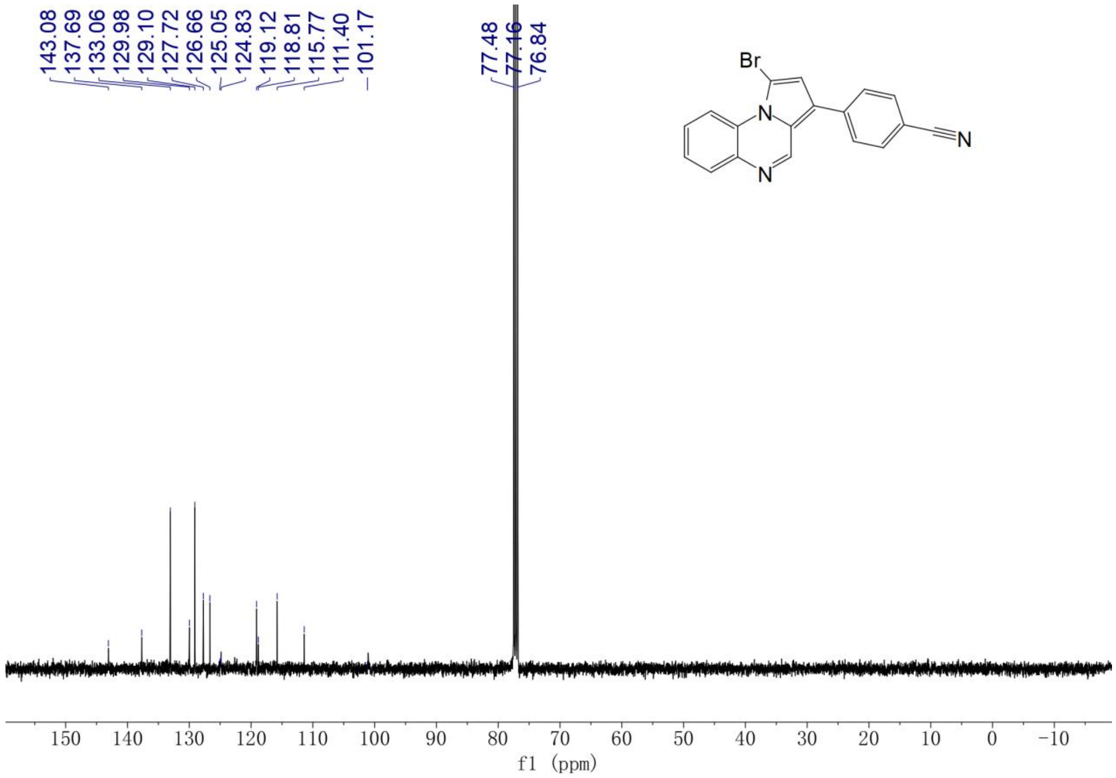

1,3-dibromopyrrolo[1,2-*a*]quinoxaline [4a]

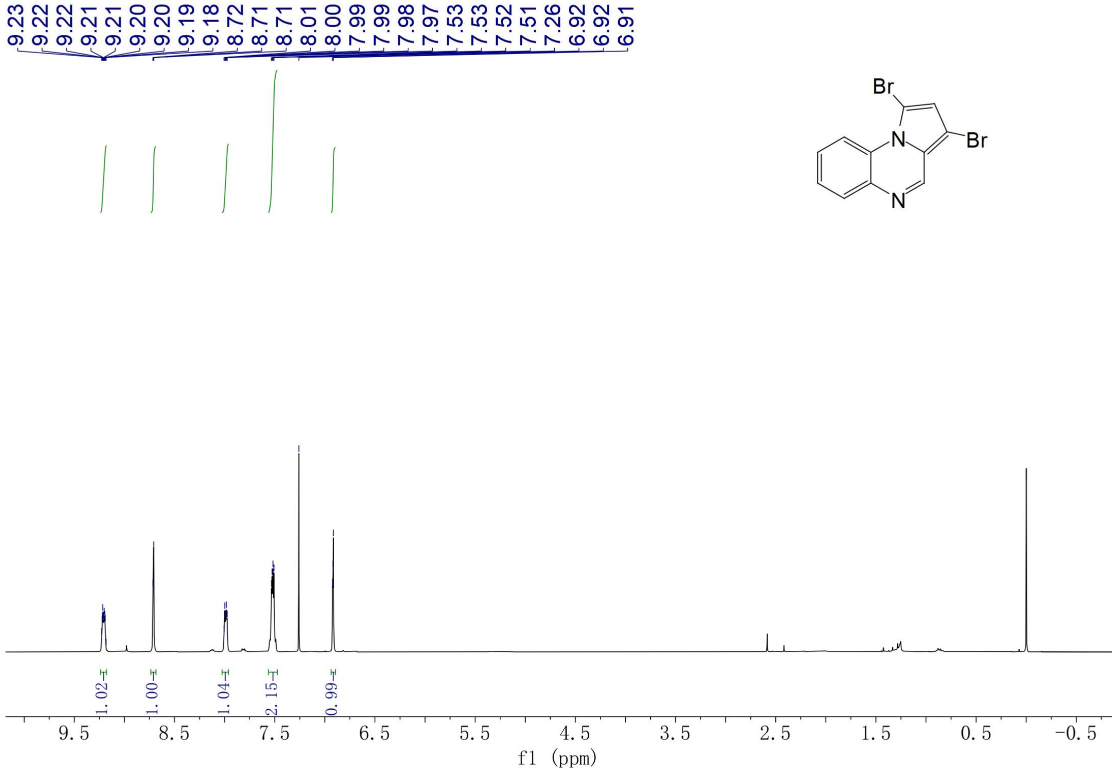

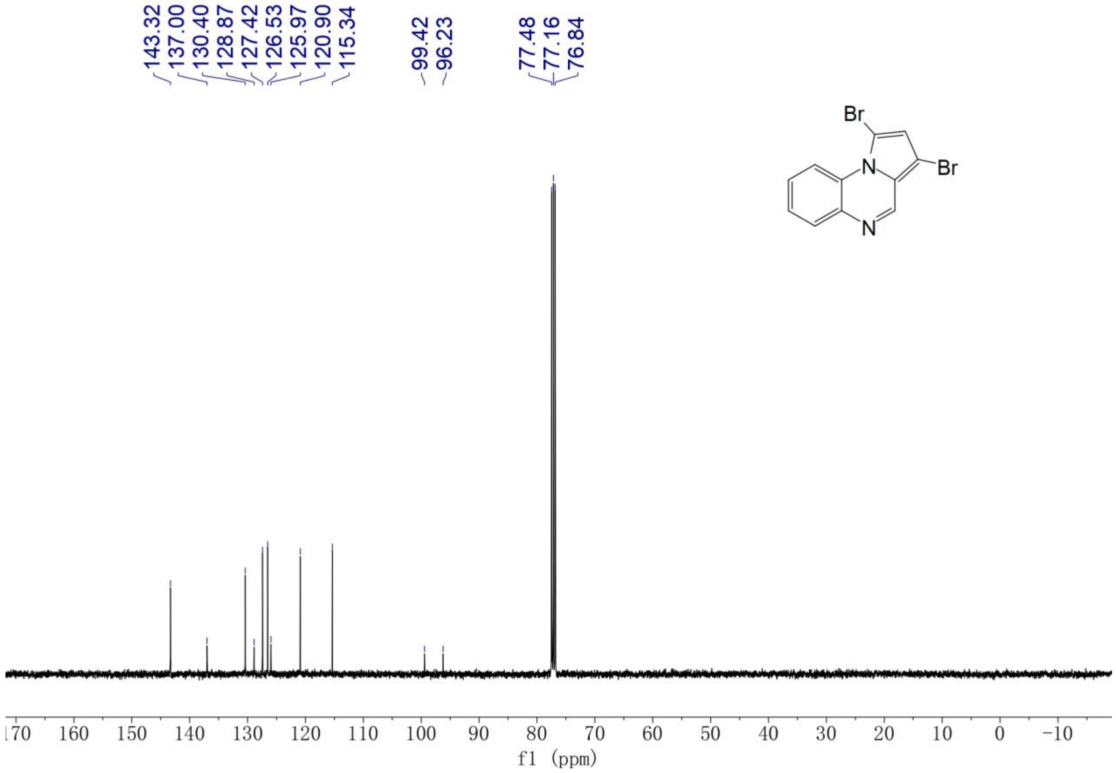

1,3-dibromo-4-phenylpyrrolo[1,2-*a*]quinoxaline [4b]

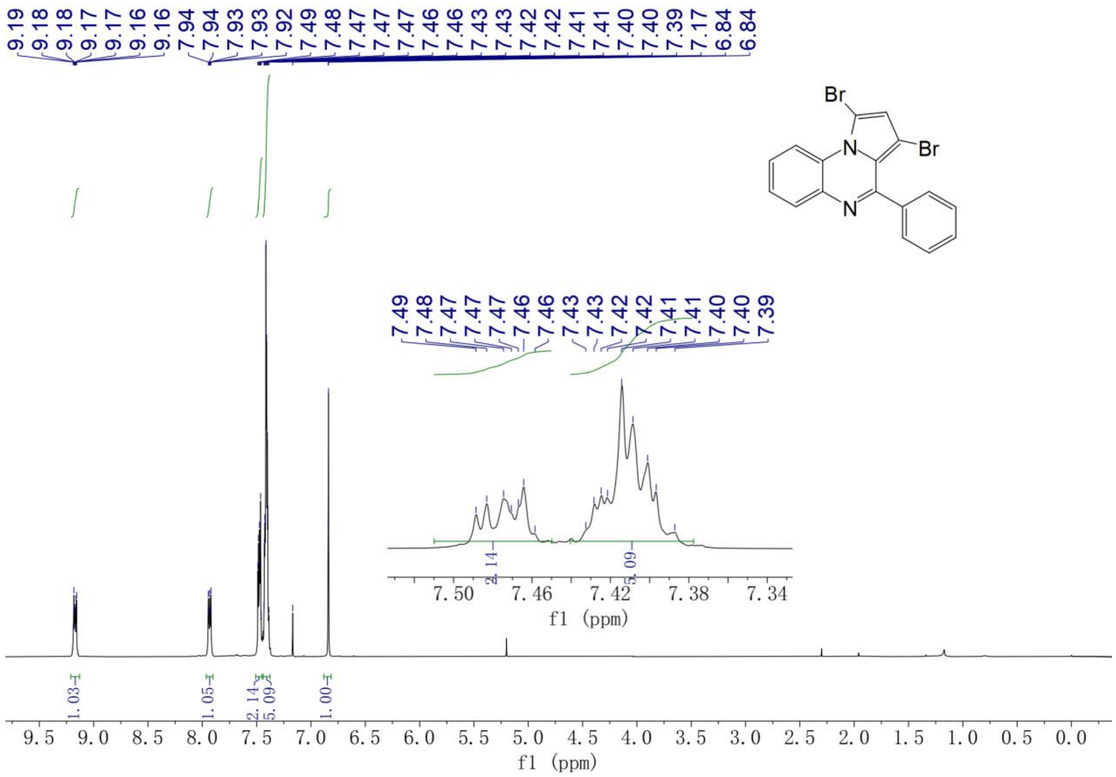

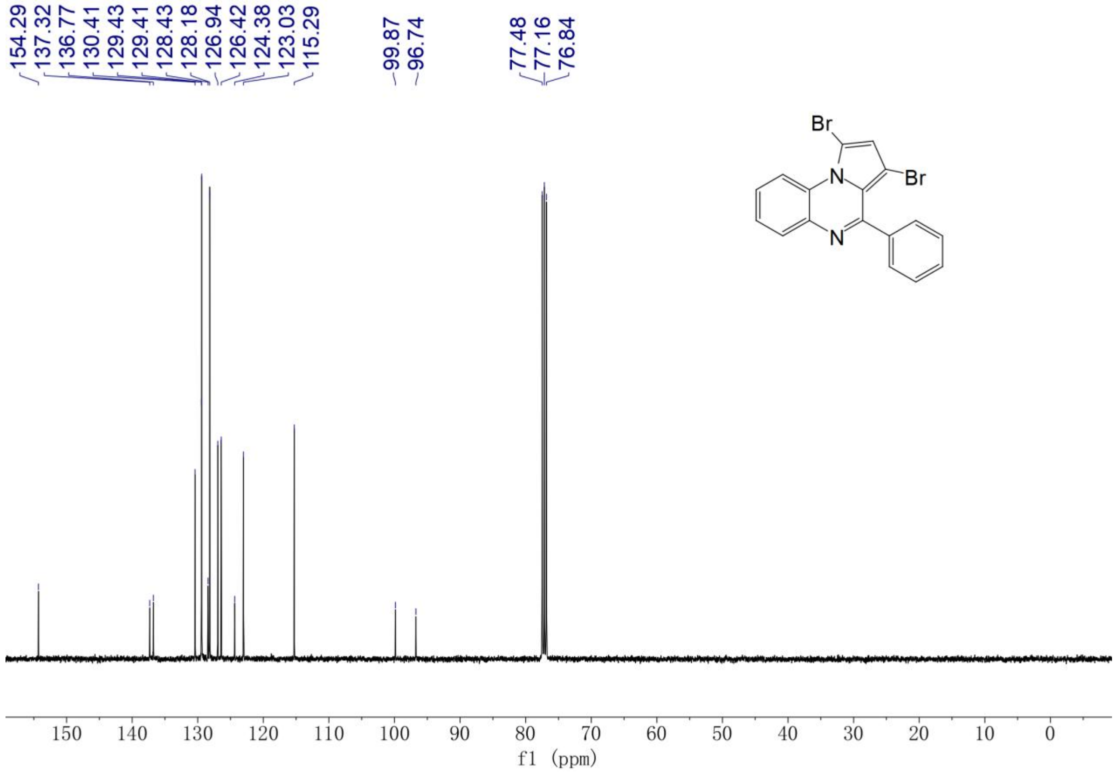

1,3-dibromo-4-(p-tolyl)pyrrolo[1,2-*a*]quinoxaline [4c]

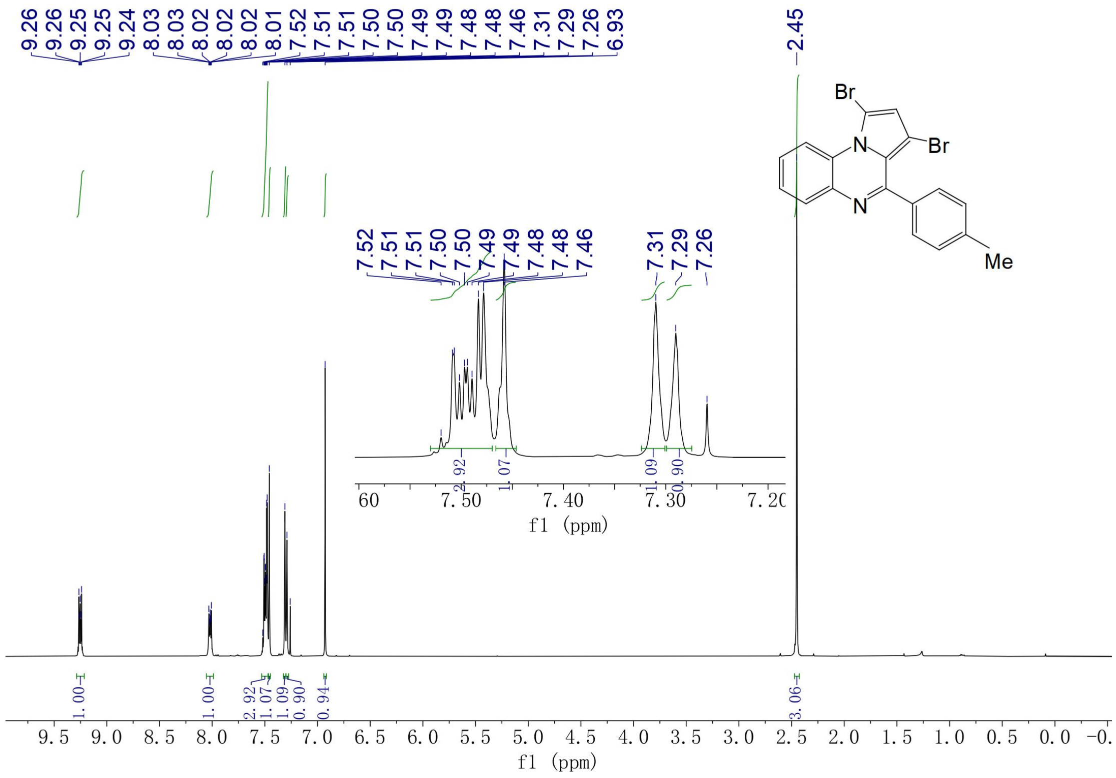

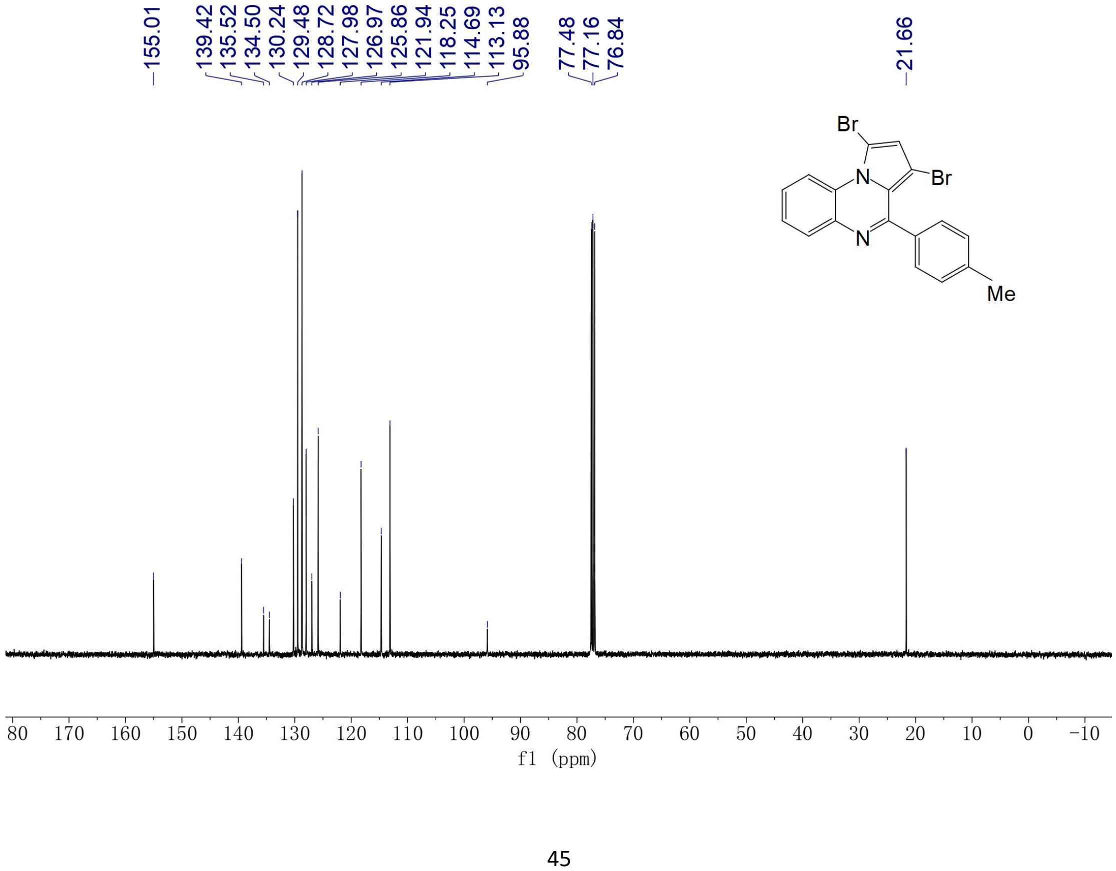

1,3-dibromo-4-(4-methoxyphenyl)pyrrolo[1,2-*a*]quinoxaline [4d]

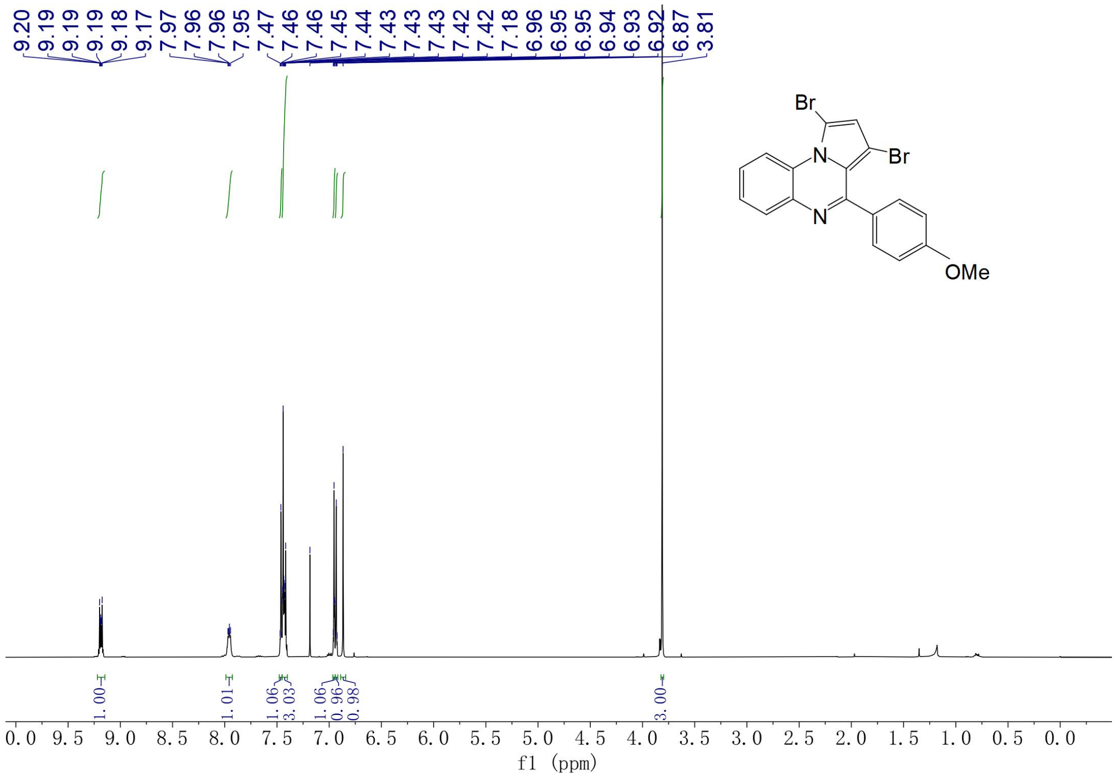

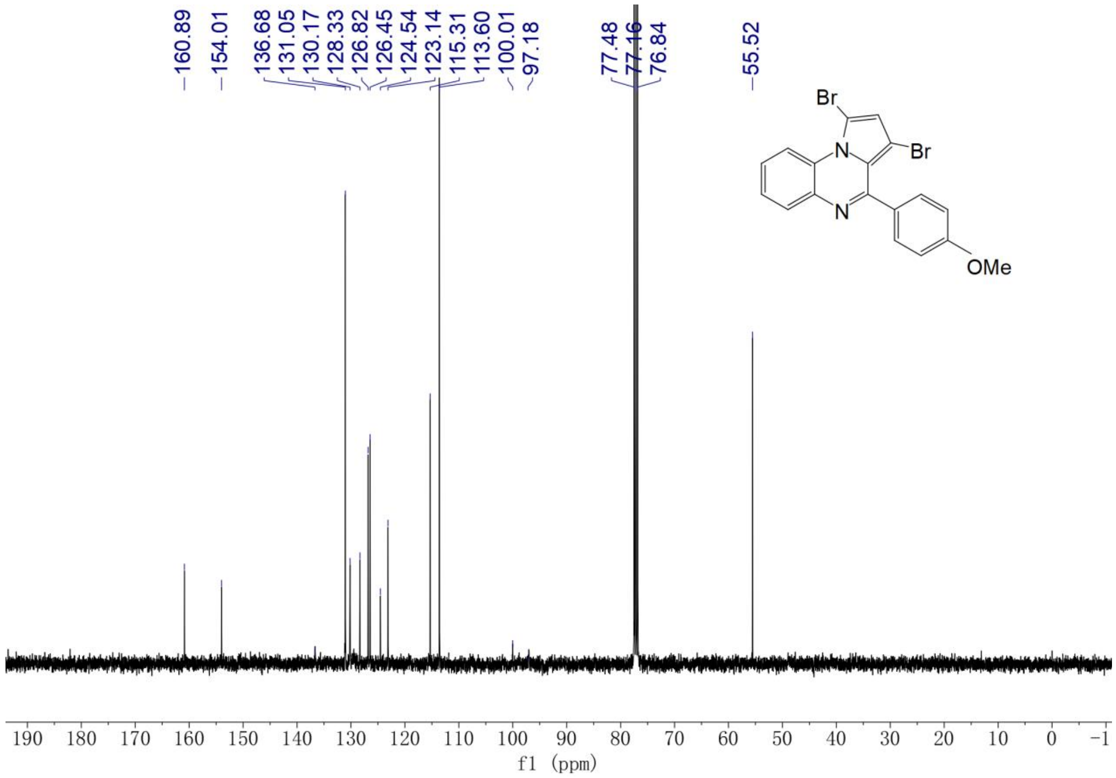

1,3-dibromo-4-(4-chlorophenyl)pyrrolo[1,2-*a*]quinoxaline [4e]

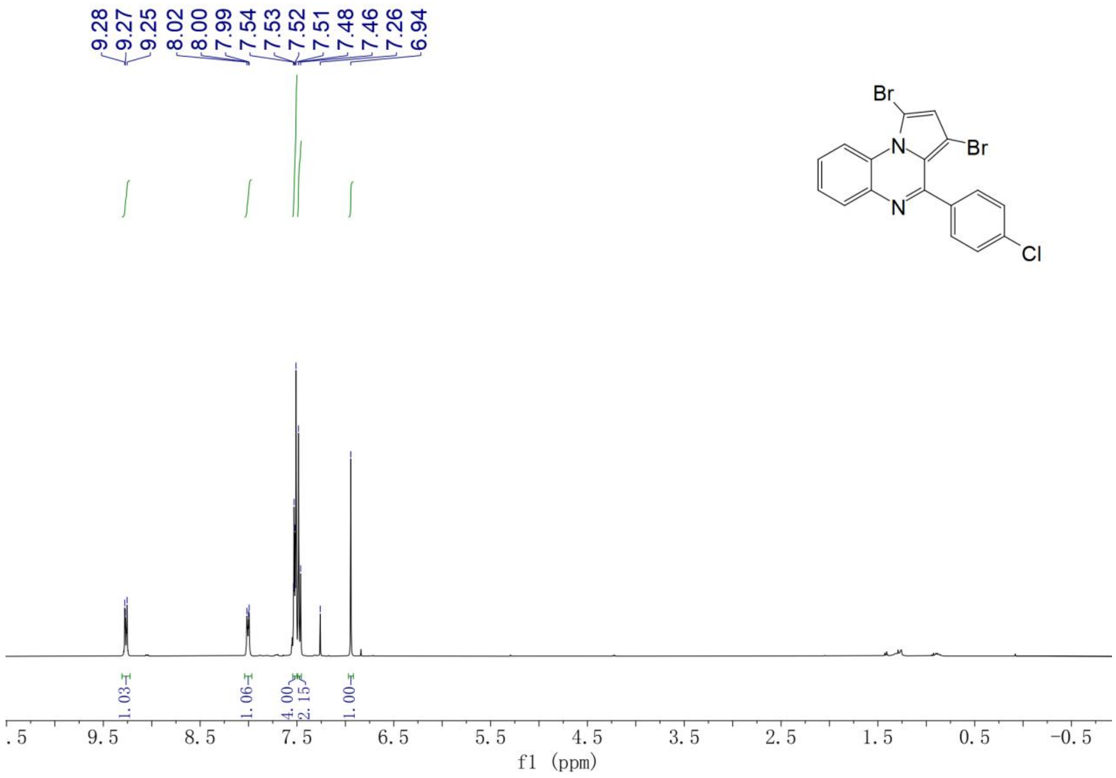

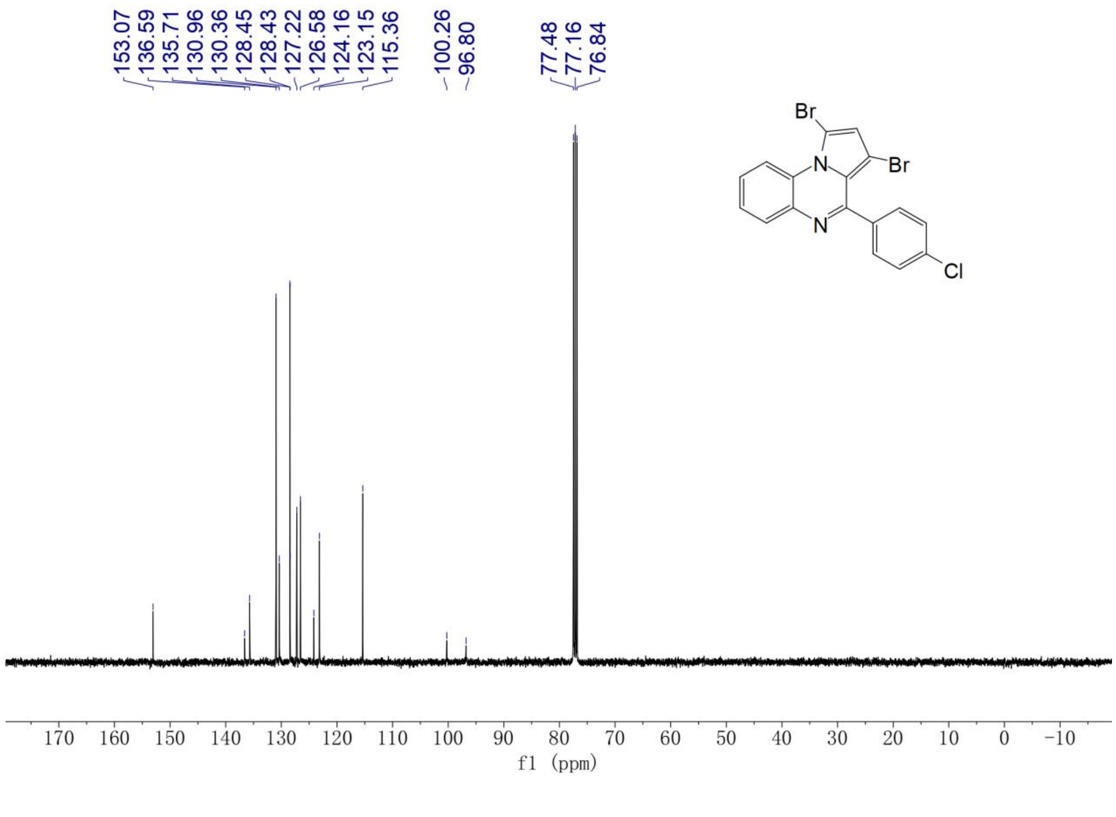

1,3-dibromo-4-(4-bromophenyl)pyrrolo[1,2-*a*]quinoxaline [4f]

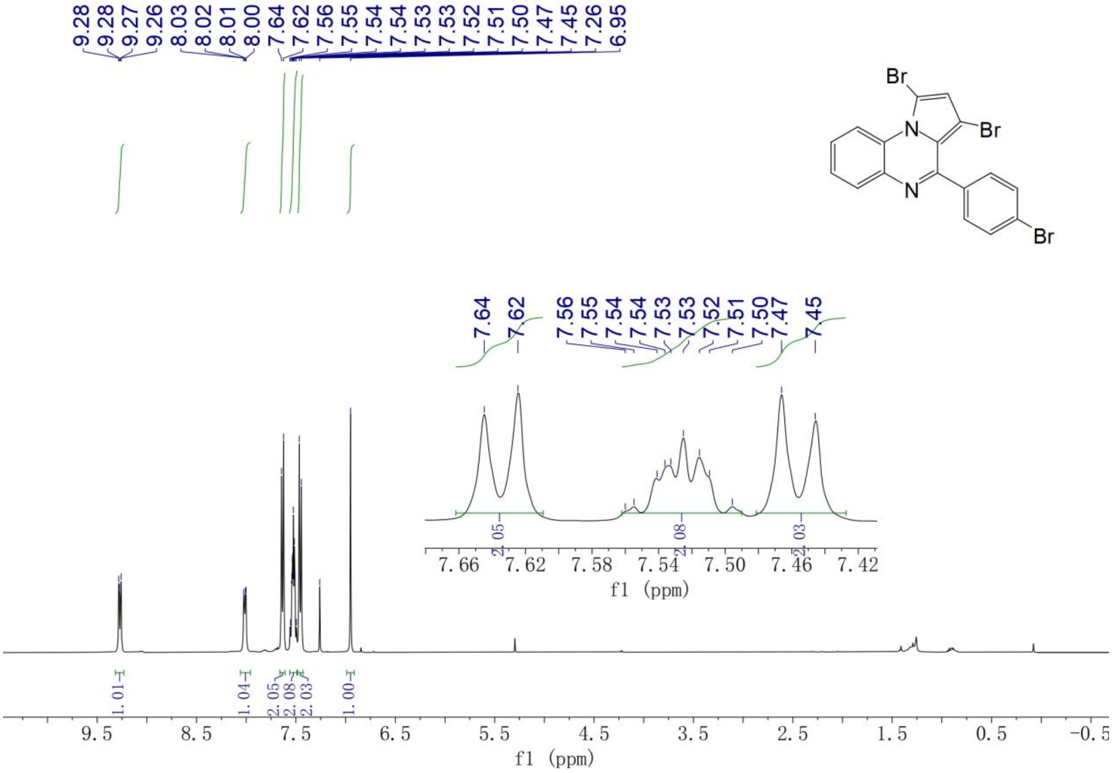

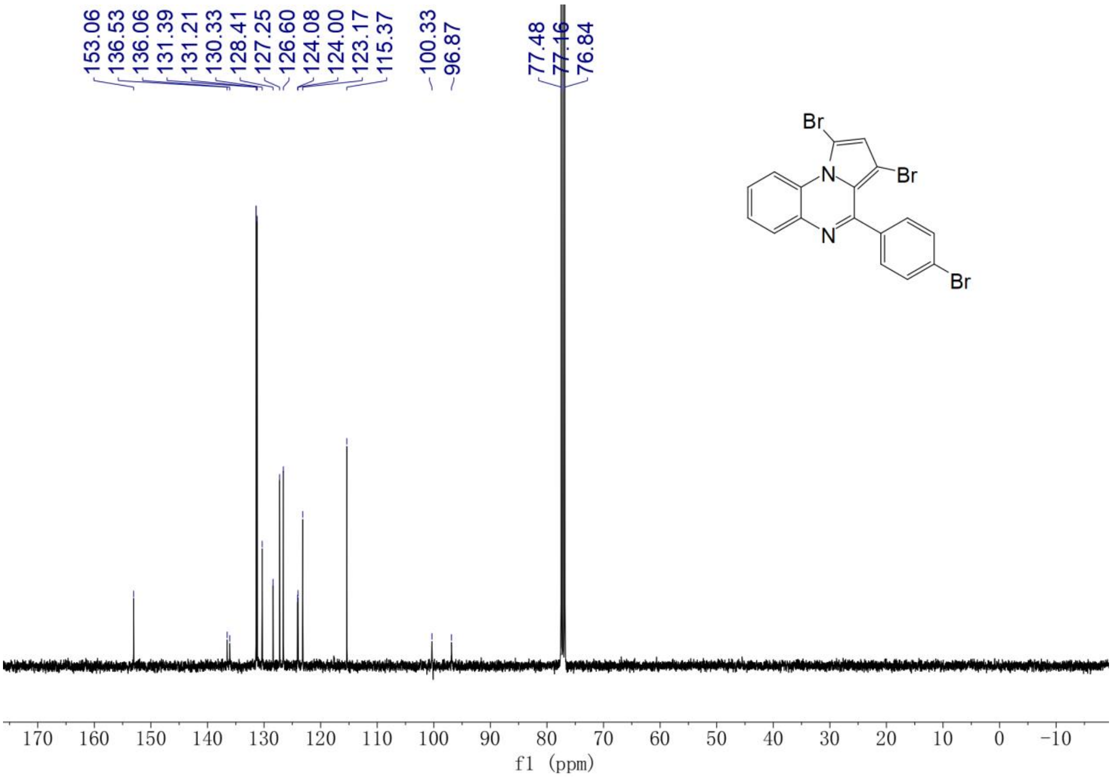

1,3-dibromo-4-(4-fluorophenyl)pyrrolo[1,2-*a*]quinoxaline [4g]

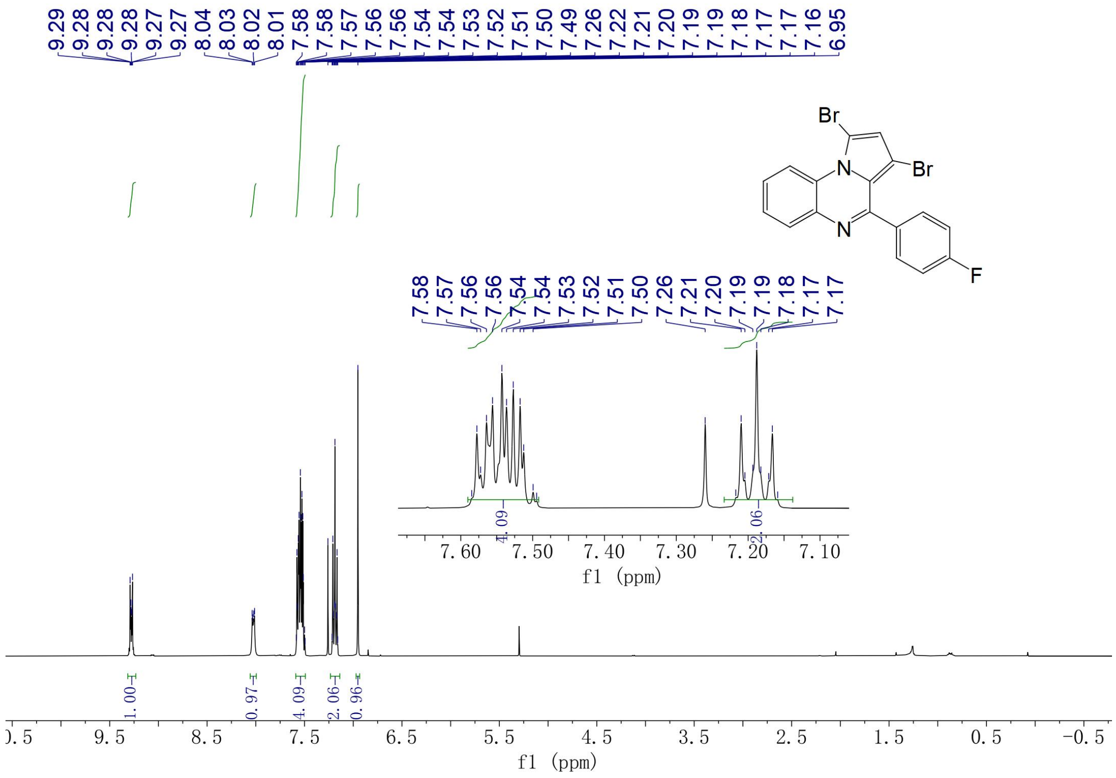

.

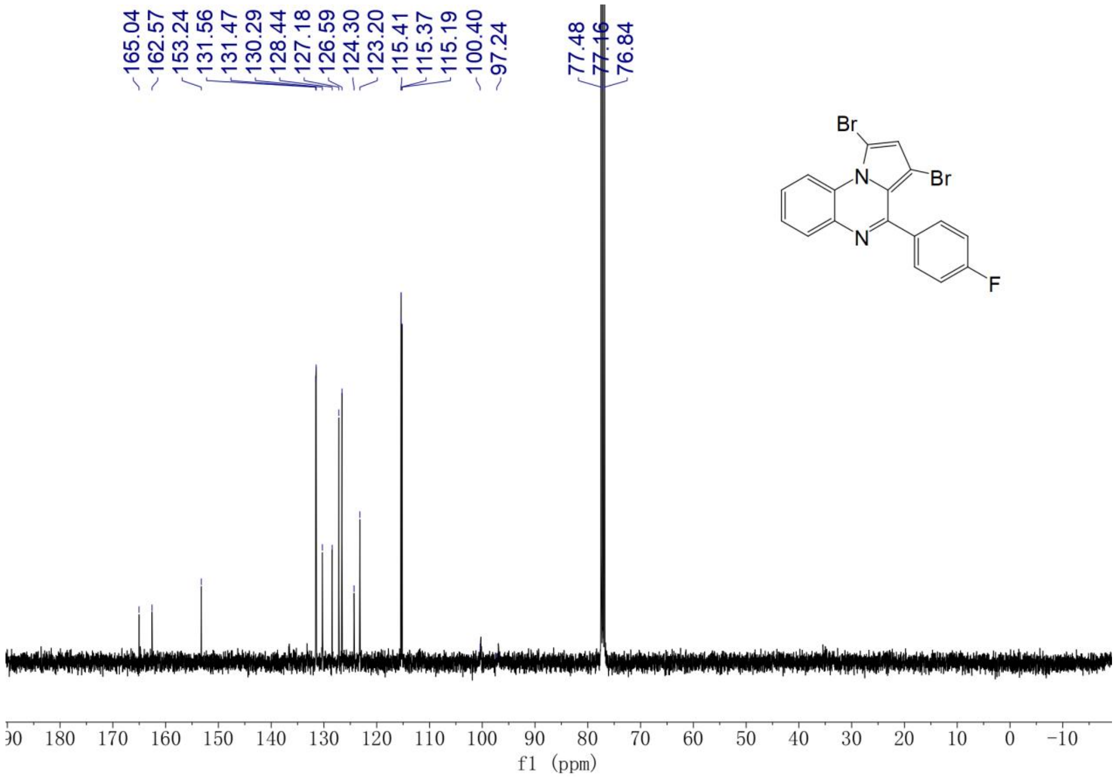

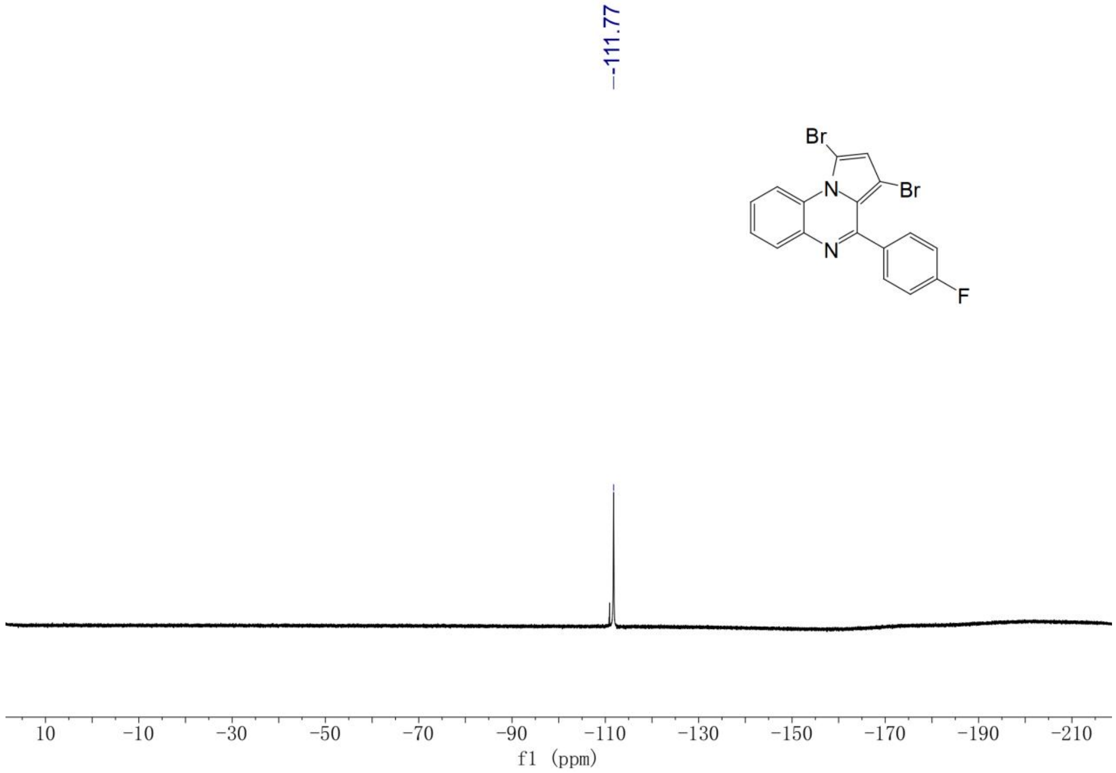

1,3-dibromo-4-(4-nitrophenyl)pyrrolo[1,2-*a*]quinoxaline [4h]

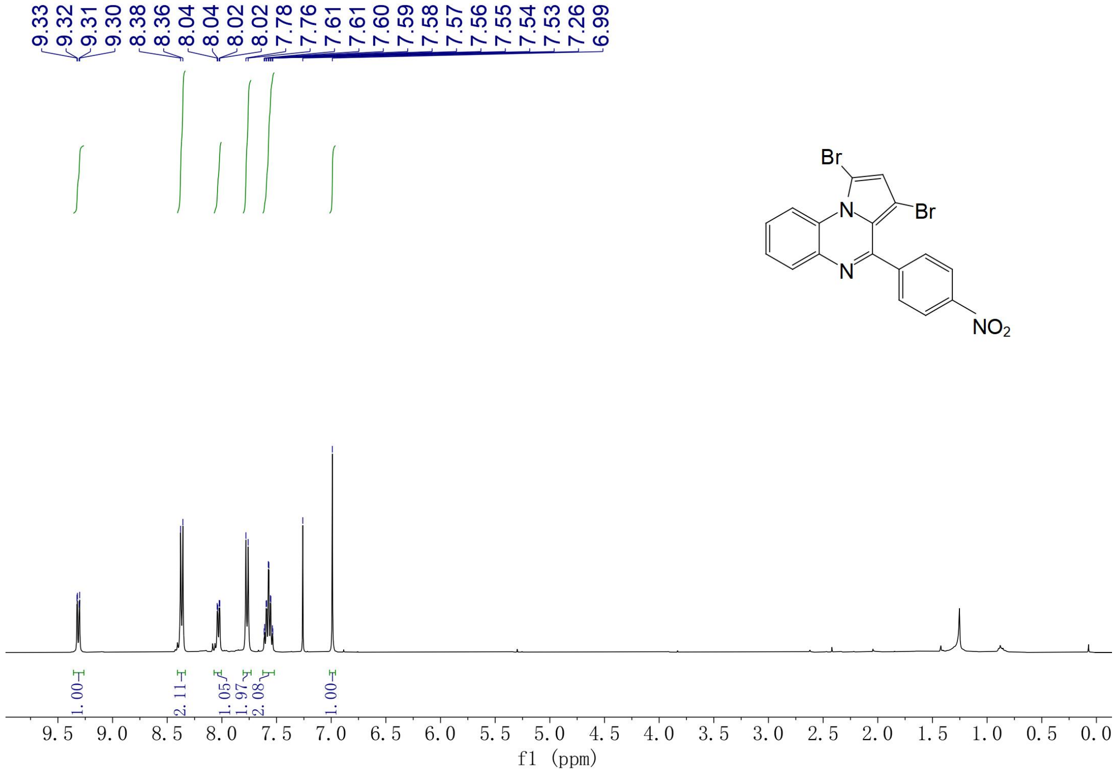

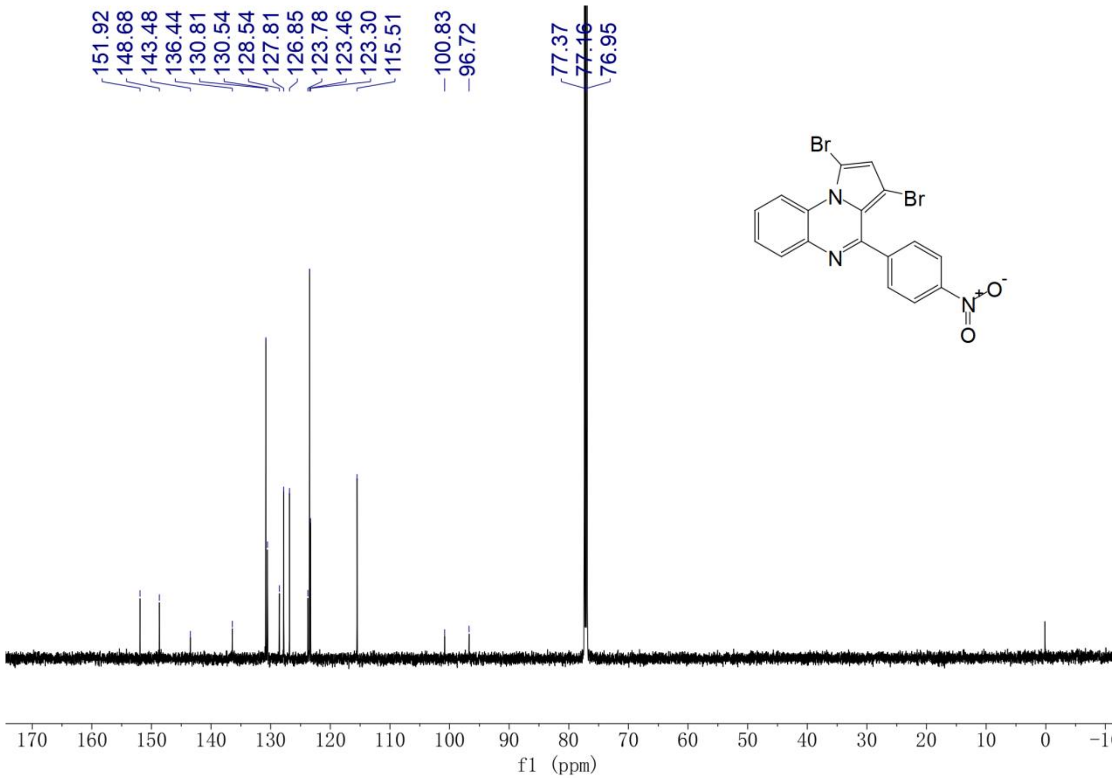

1,3-dibromo-4-(2,4-dichlorophenyl)pyrrolo[1,2-*a*]quinoxaline [4i]

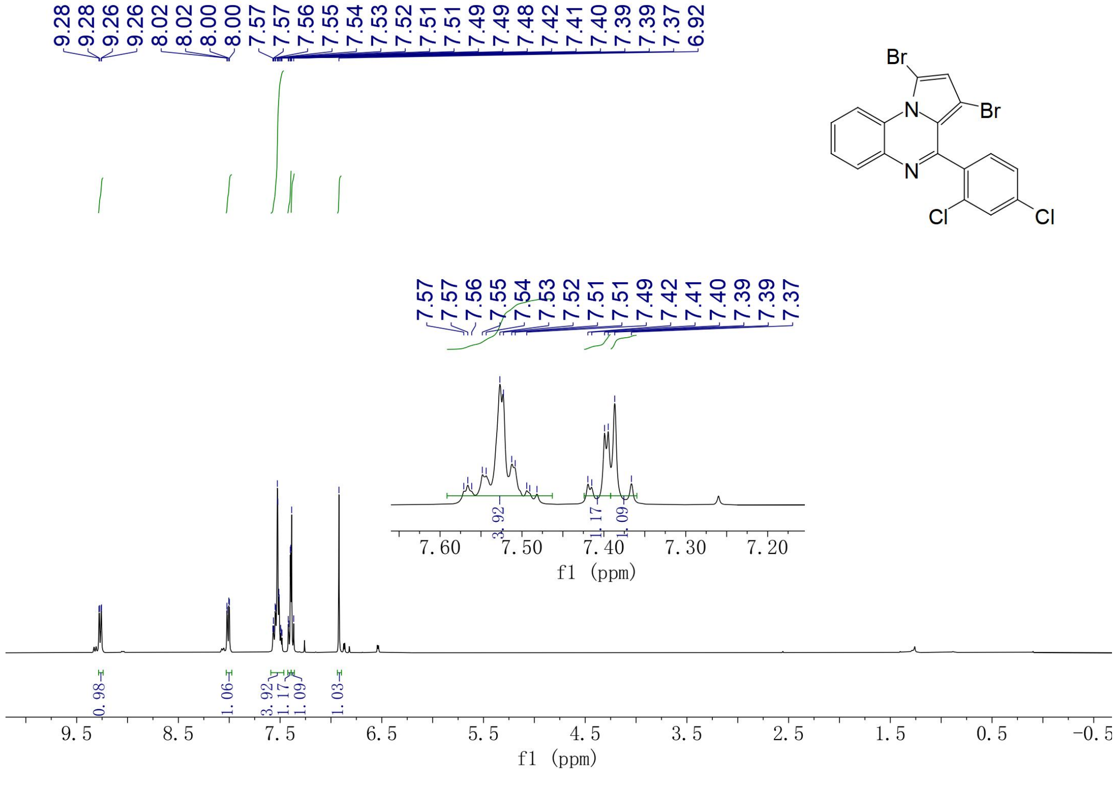

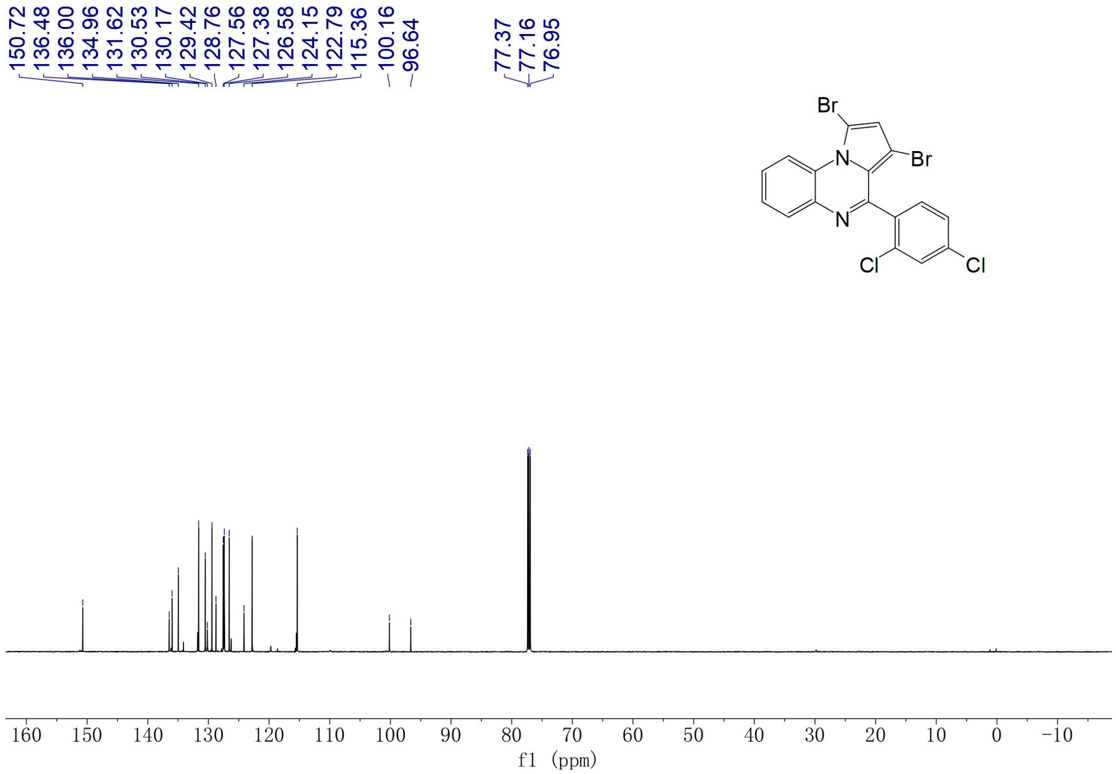

1,3-dibromo-7-methylpyrrolo[1,2-*a*]quinoxaline [4j]

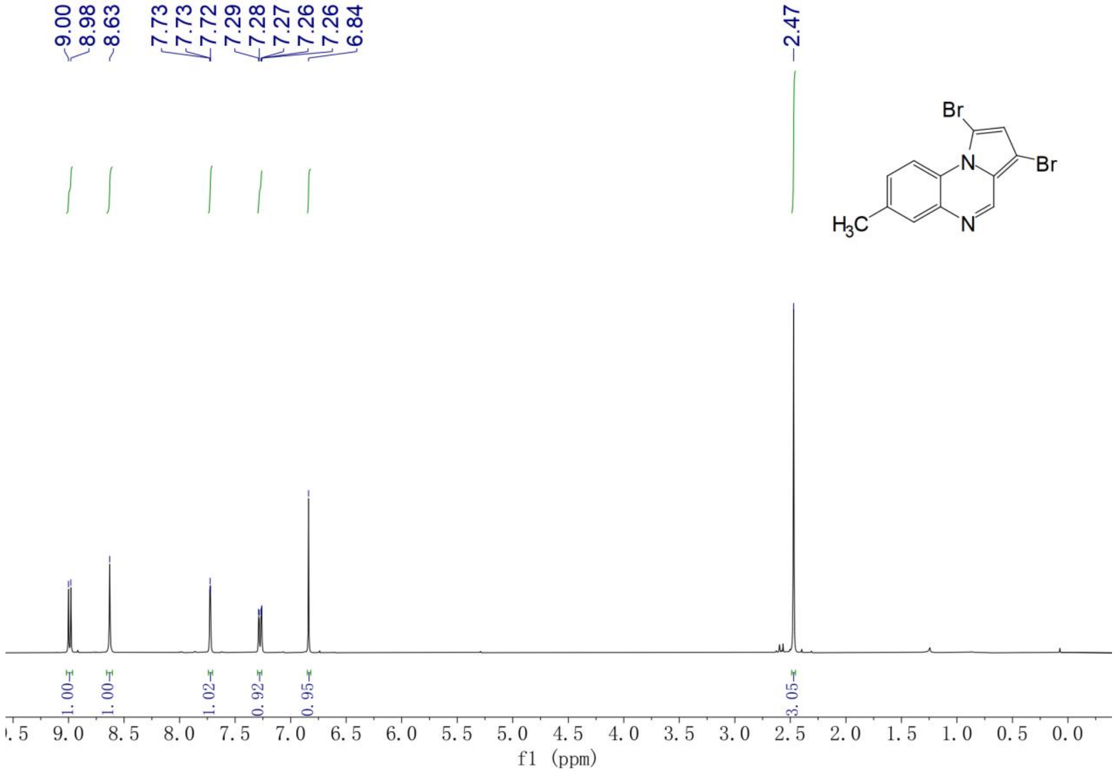

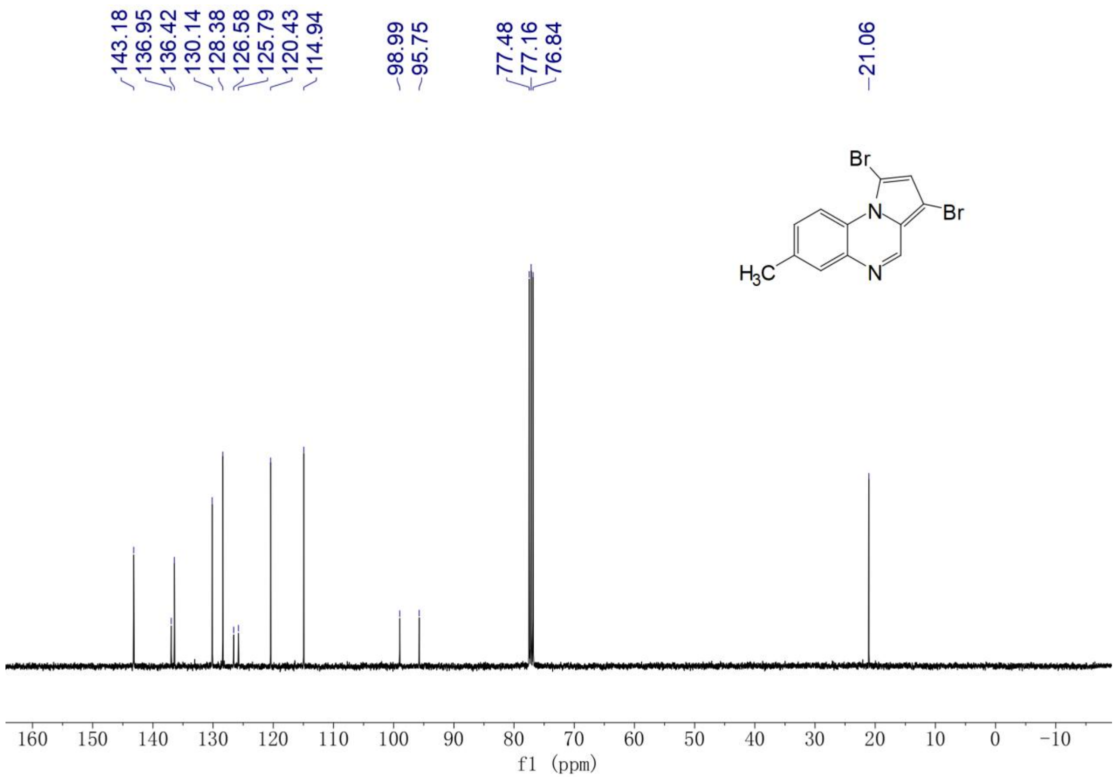

1,3-dibromo-7-chloropyrrolo[1,2-*a*]quinoxaline [4k]

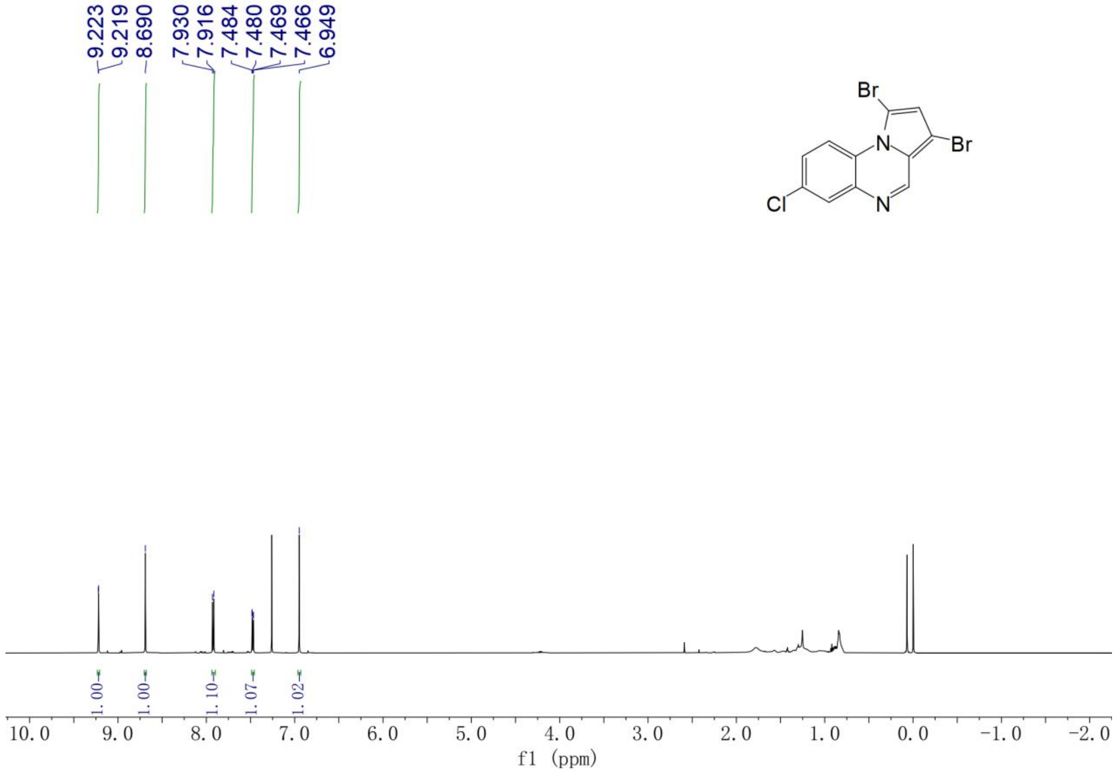

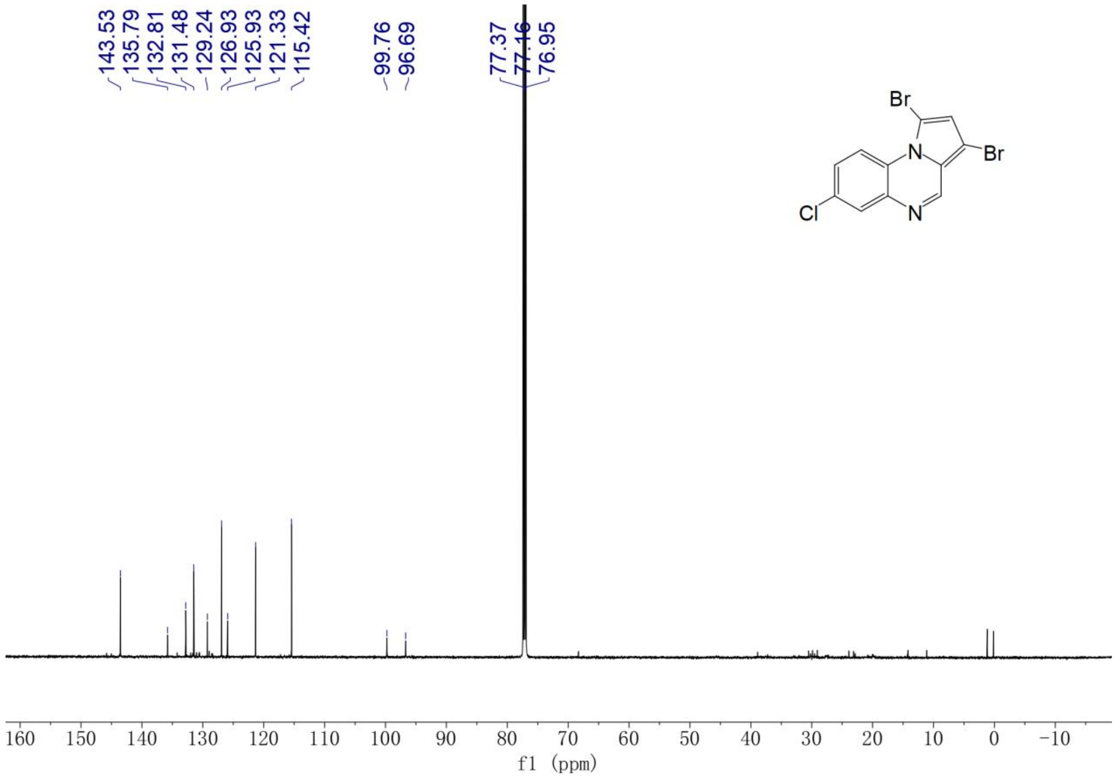

1,3-dibromo-8-methylpyrrolo[1,2-*a*]quinoxaline [4l]

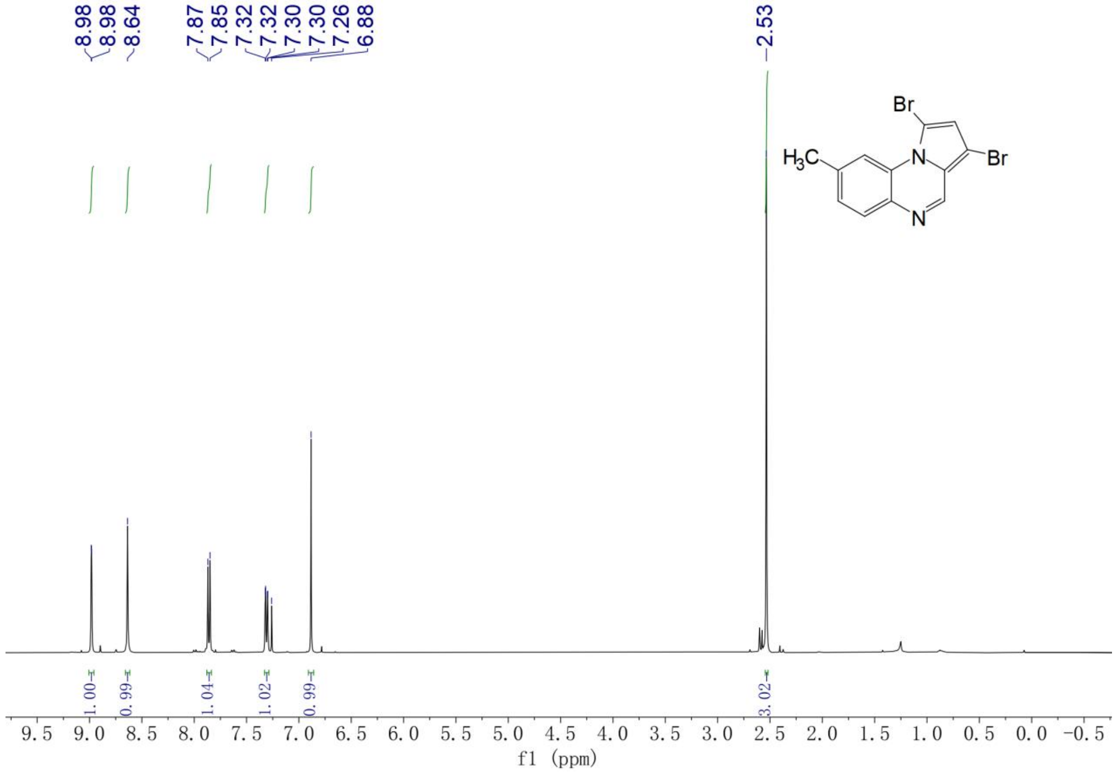

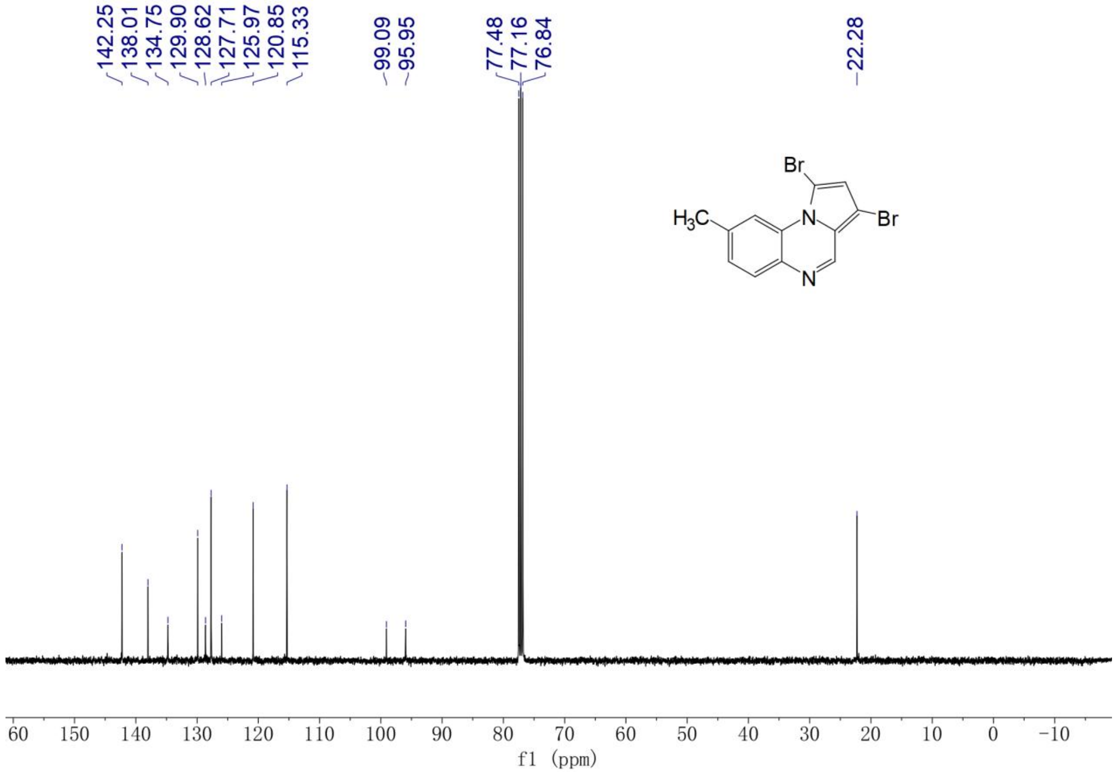

1,3-dibromo-8-chloropyrrolo[1,2-*a*]quinoxaline [4m]

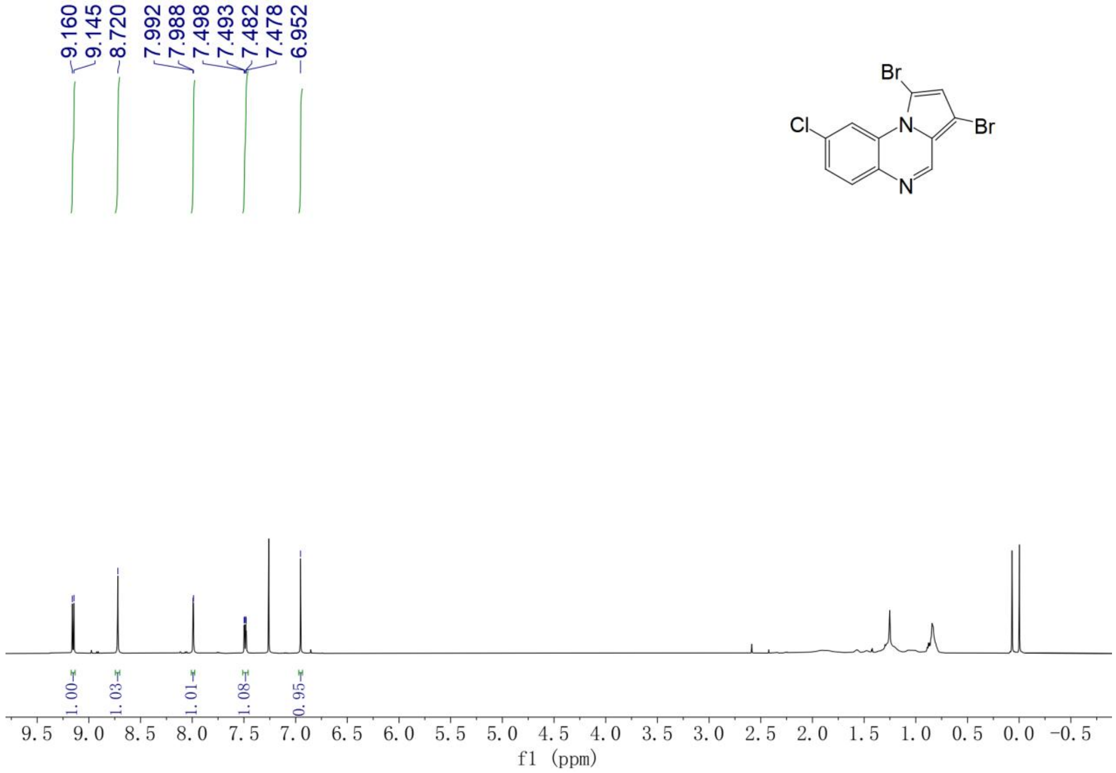

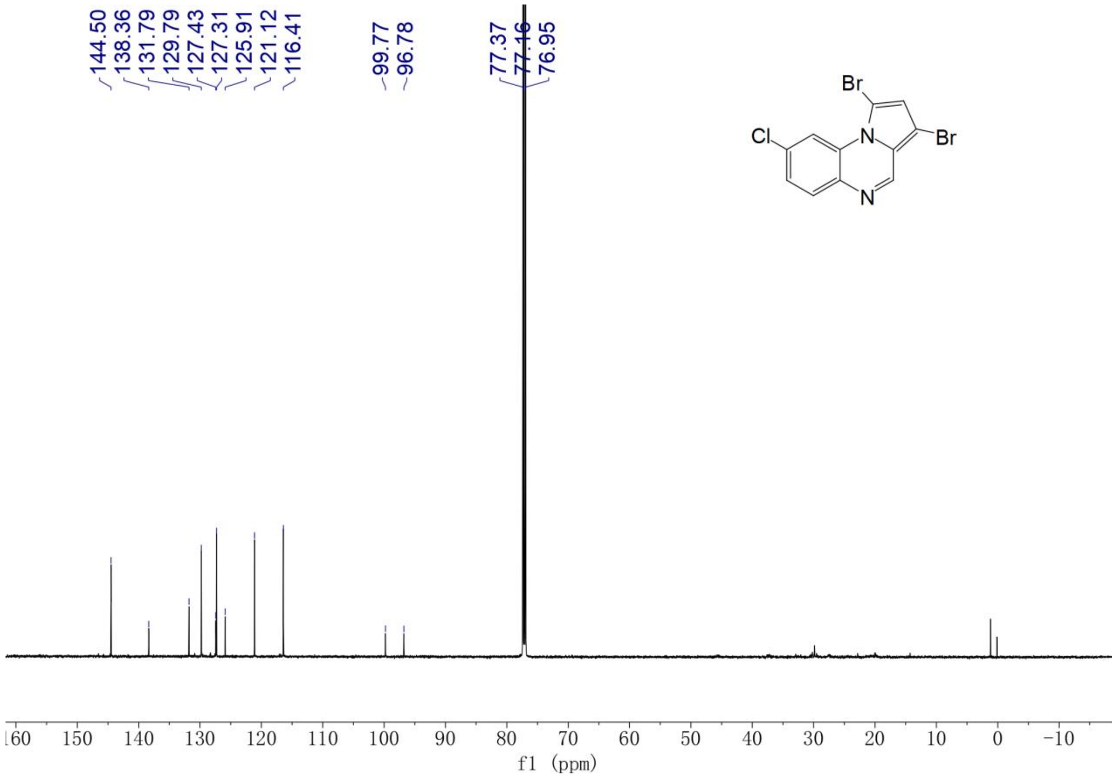

1,3-dibromo-8-fluoropyrrolo[1,2-*a*]quinoxaline [4n]

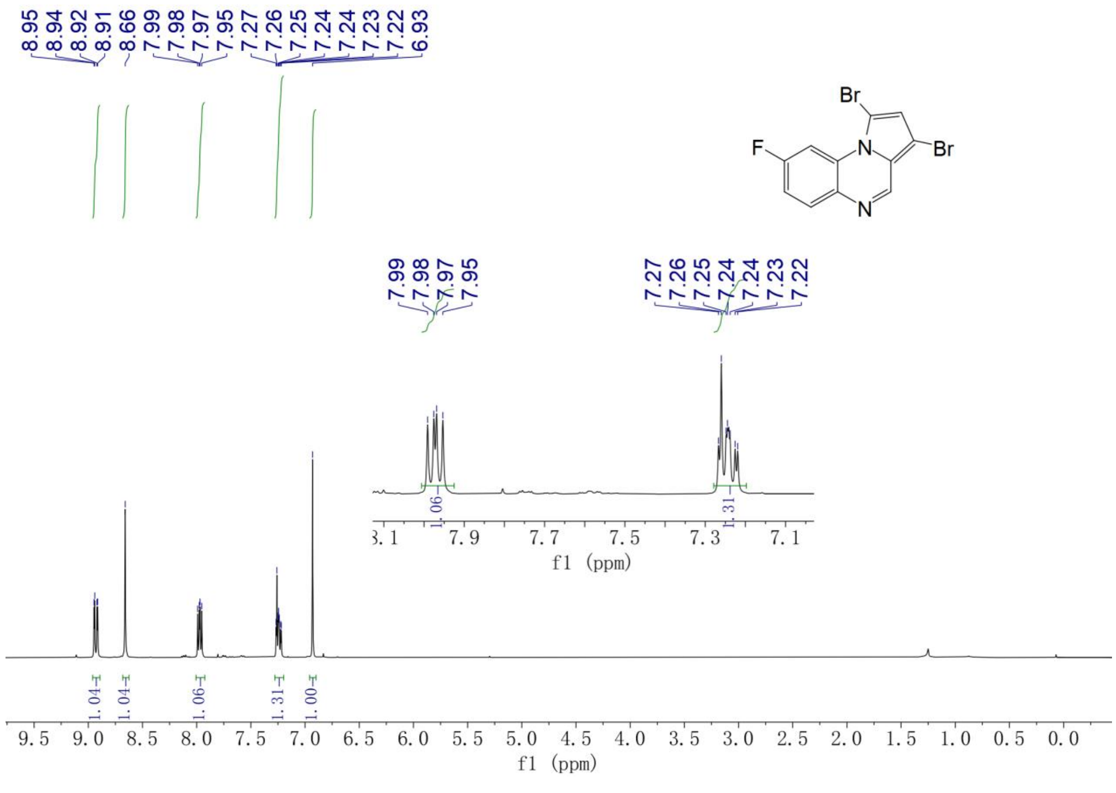

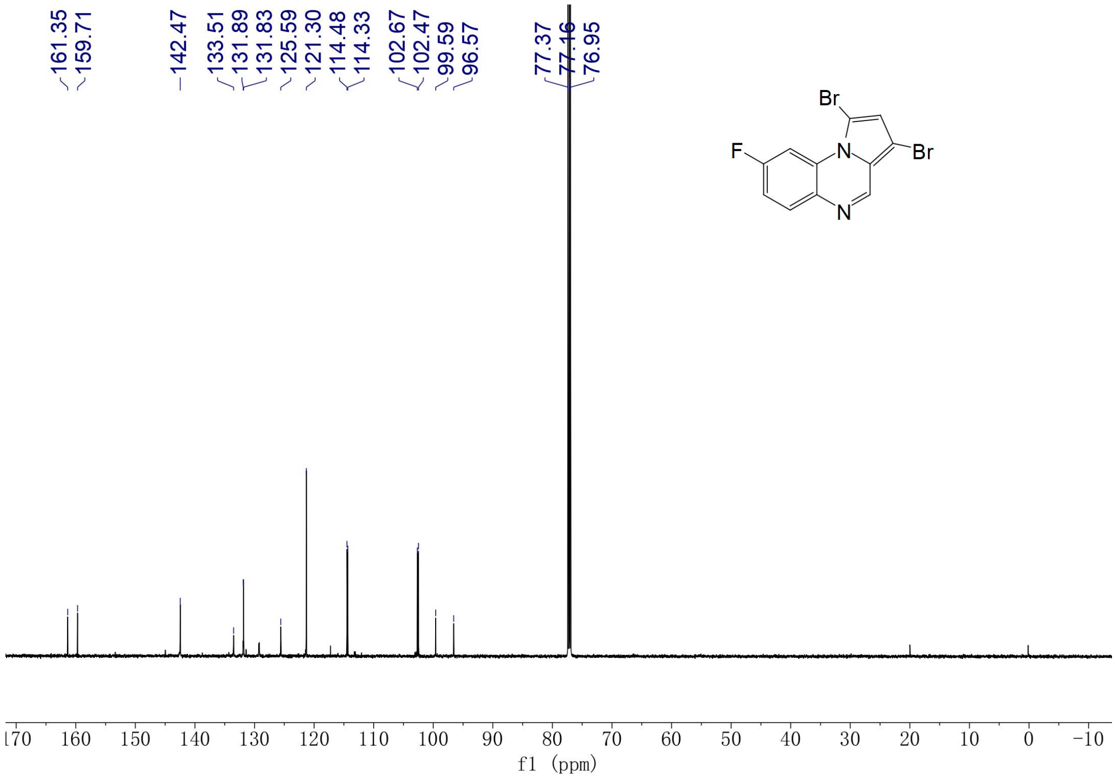
161.35
159.71
142.47
133.51
131.89
131.83
125.59
121.30
114.48
114.33
102.67
102.47
99.59
96.57
77.37
77.16
76.95
Br
F
N
Br
N
f1 (ppm)

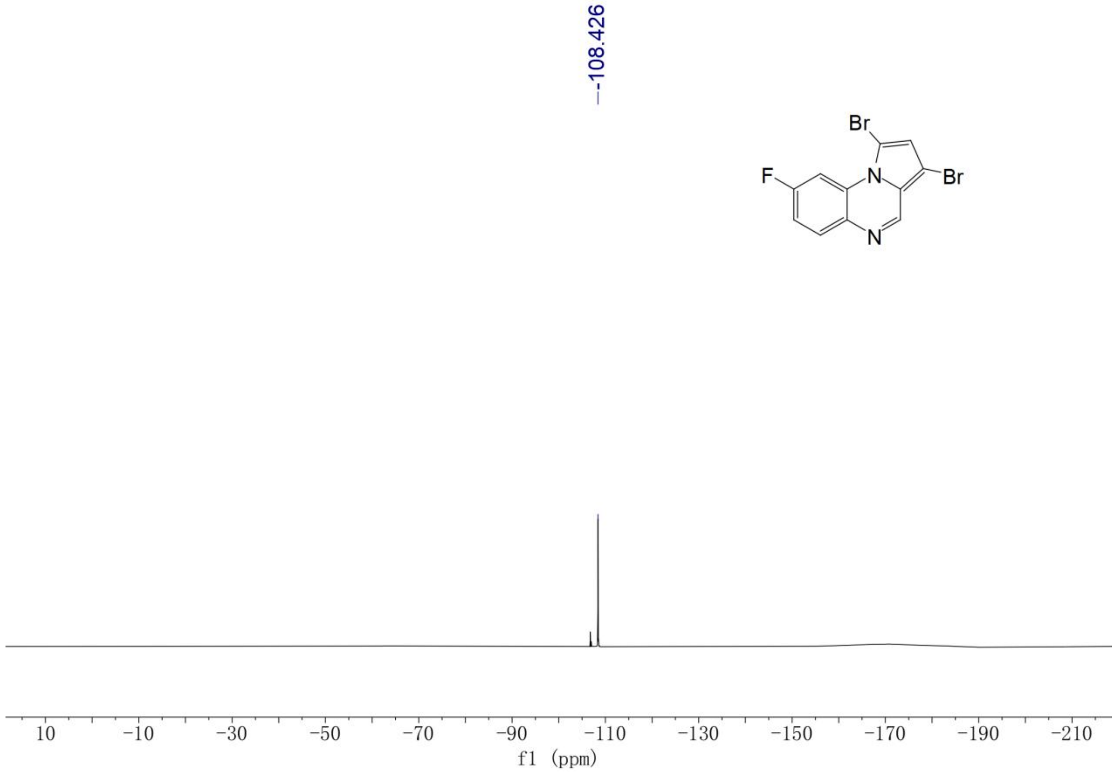
-108.426
Br
F
N
Br
N
f1 (ppm)

1,3-dibromo-4-(4-methoxyphenyl)-7-methylpyrrolo[1,2-*a*]quinoxaline [4o]

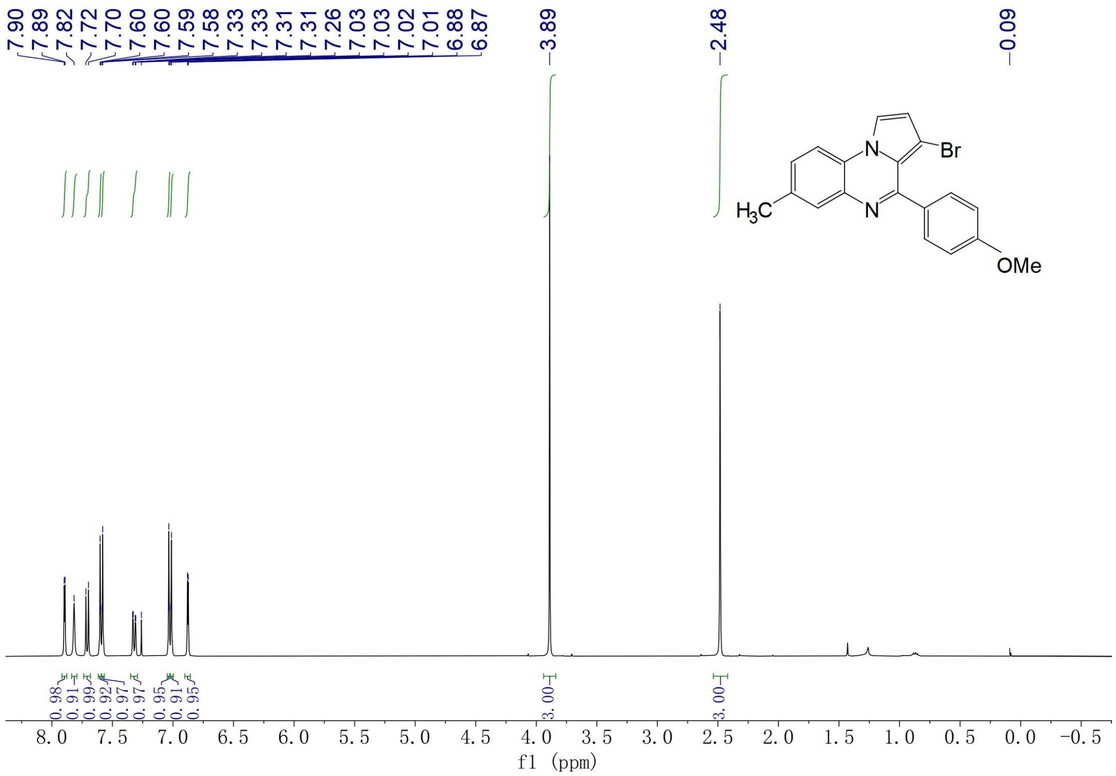

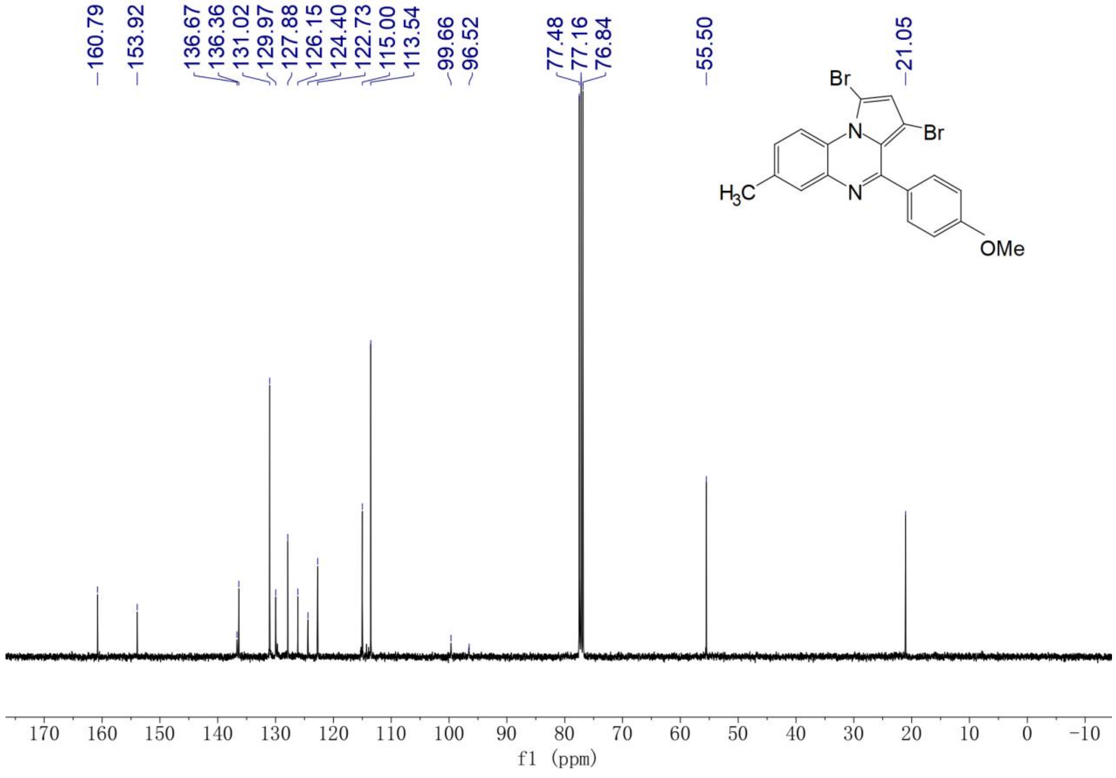

1,3-dibromo-4-(4-bromophenyl)-7-methylpyrrolo[1,2-*a*]quinoxaline [4q]

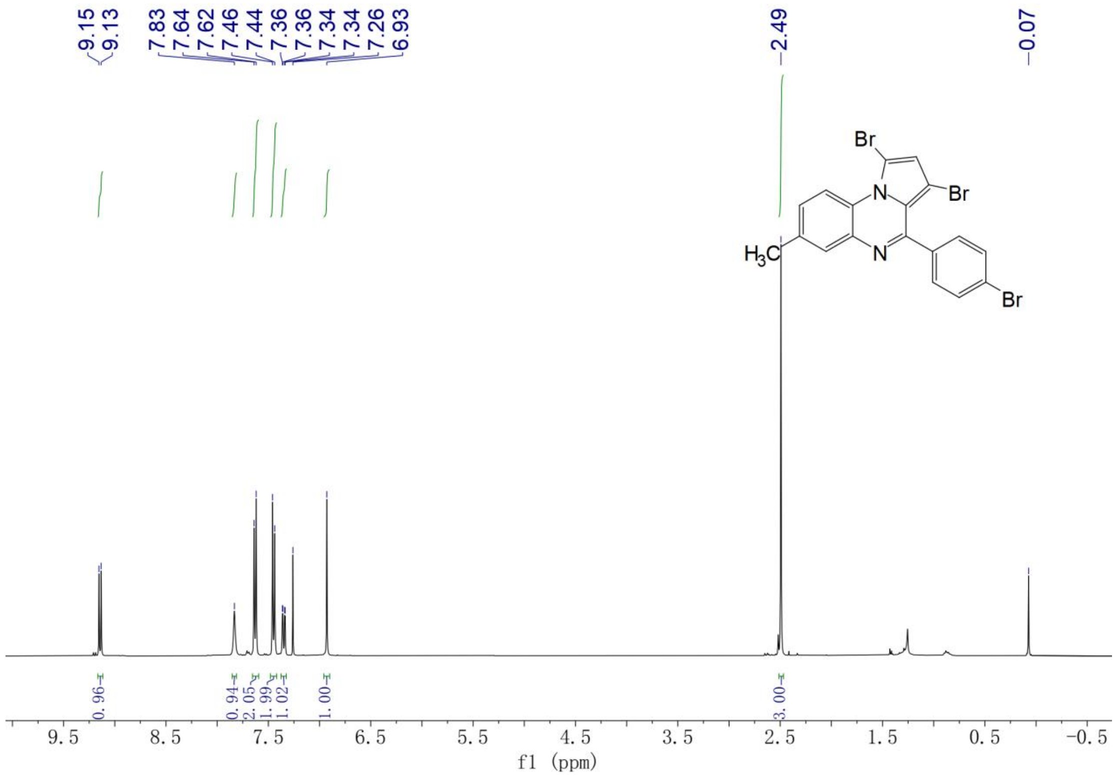

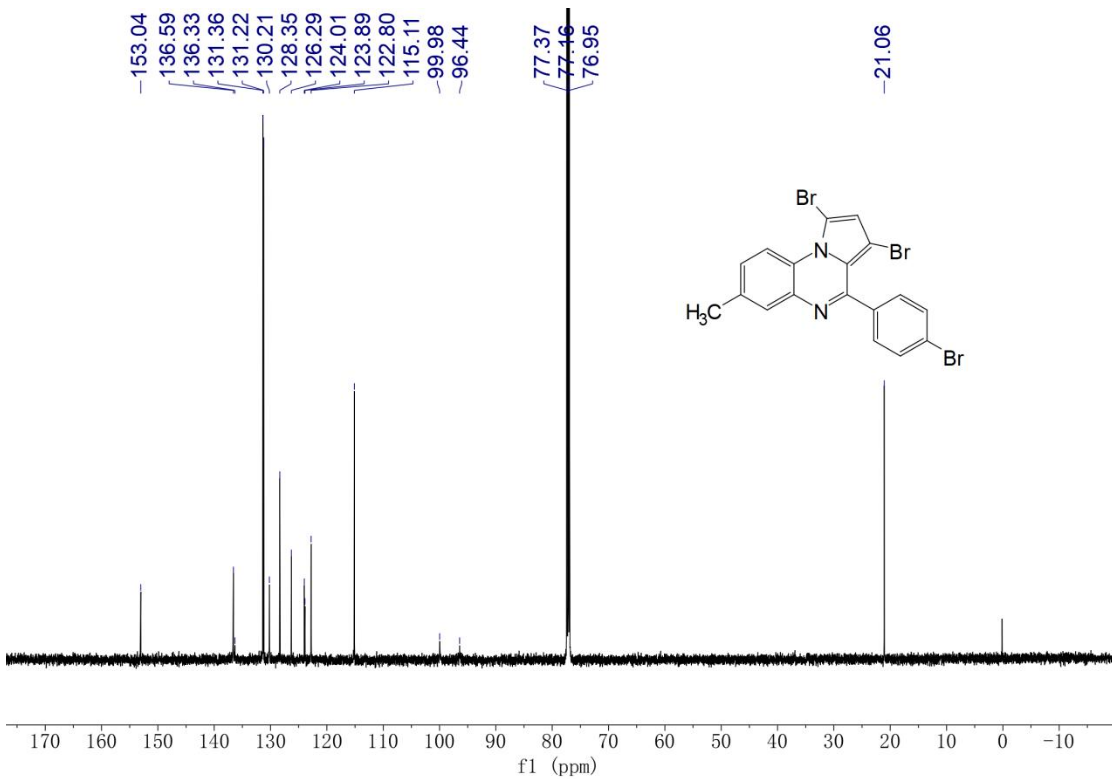

3-phenylpyrrolo[1,2-*a*]quinoxaline [3aa]

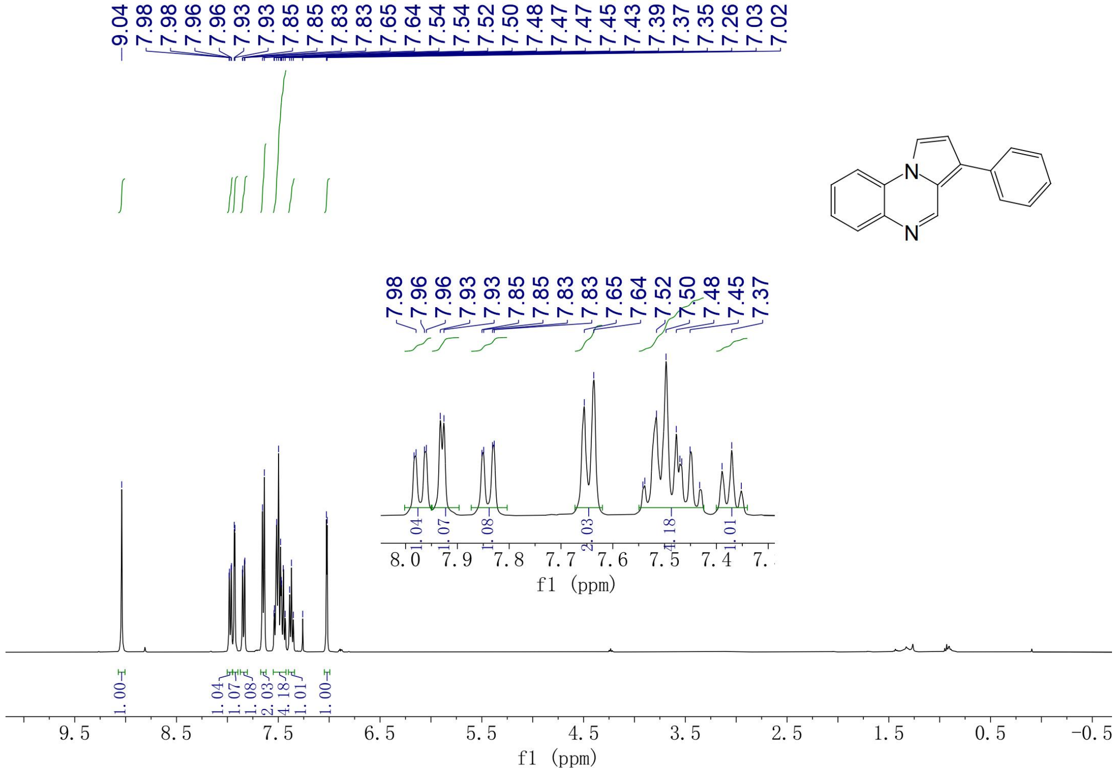

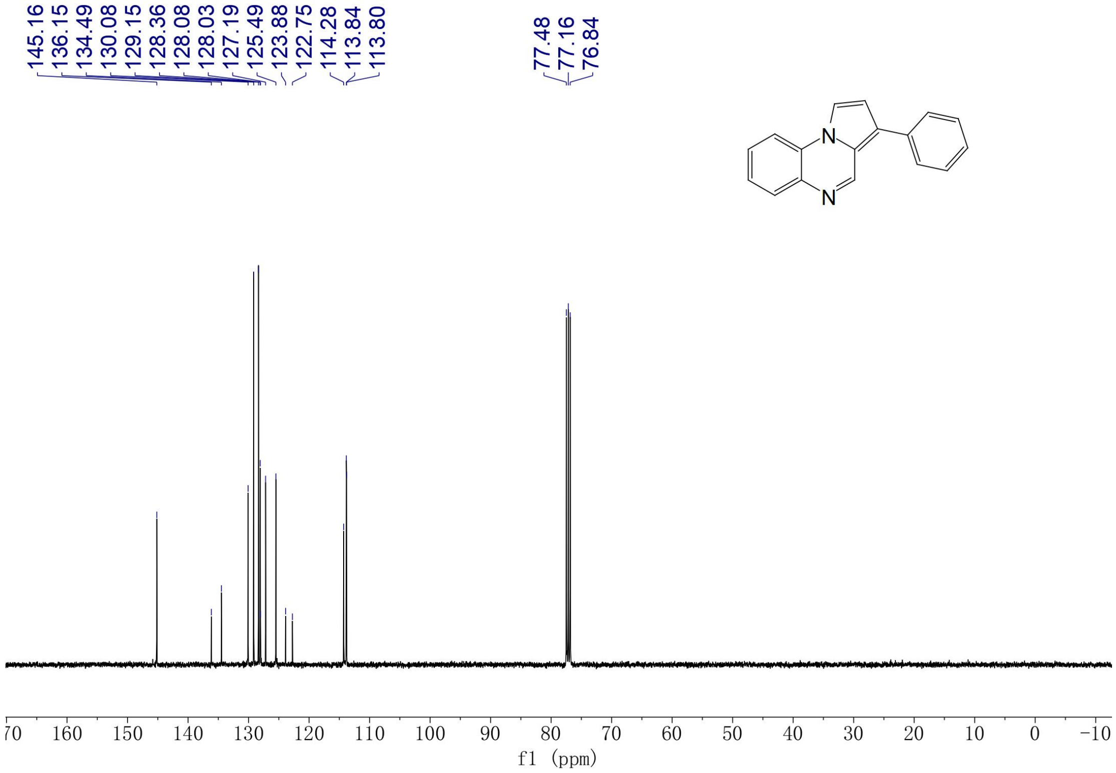

3-bromo-1-(p-tolyl)pyrrolo[1,2-*a*]quinoxaline [3ab]

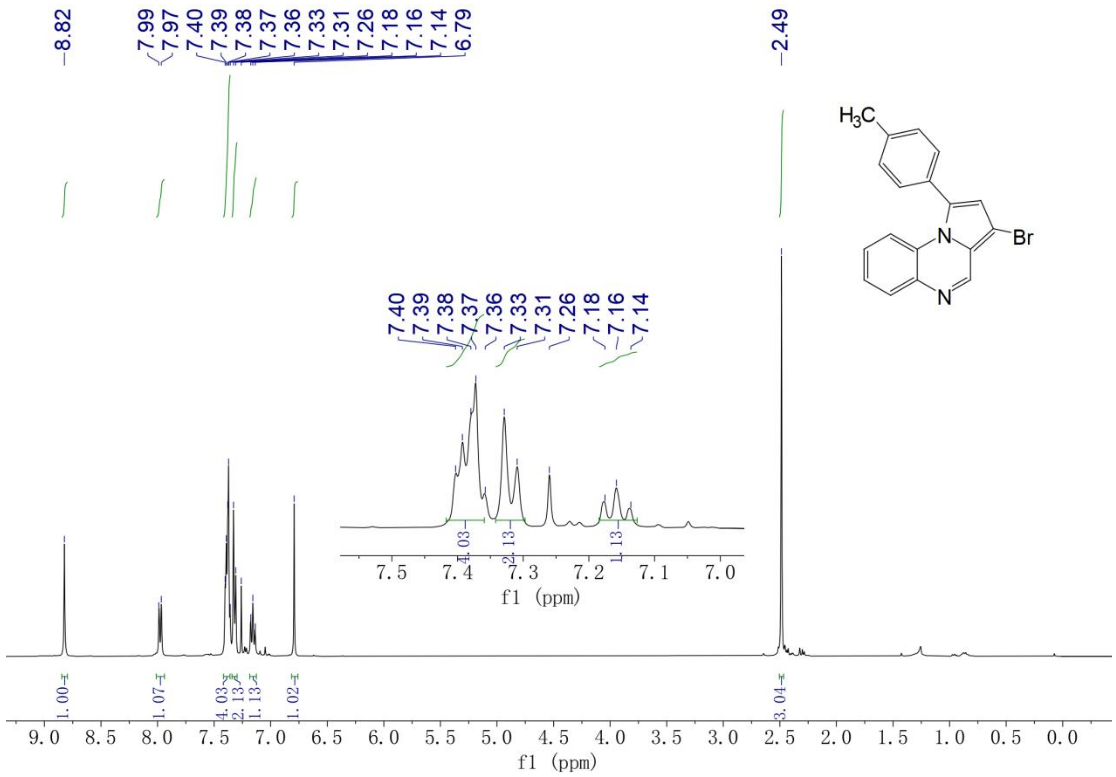

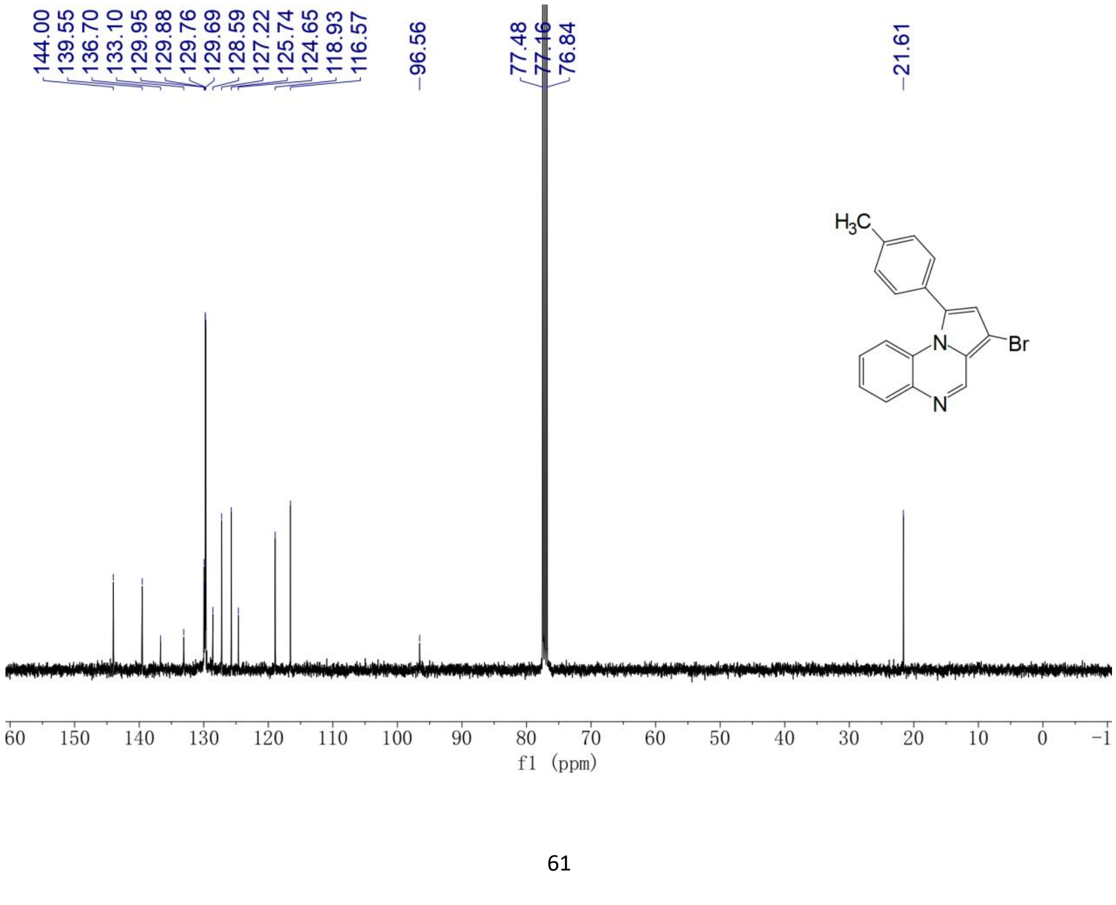

3-bromo-1-chloropyrrolo[1,2-*a*]quinoxaline [3ac]

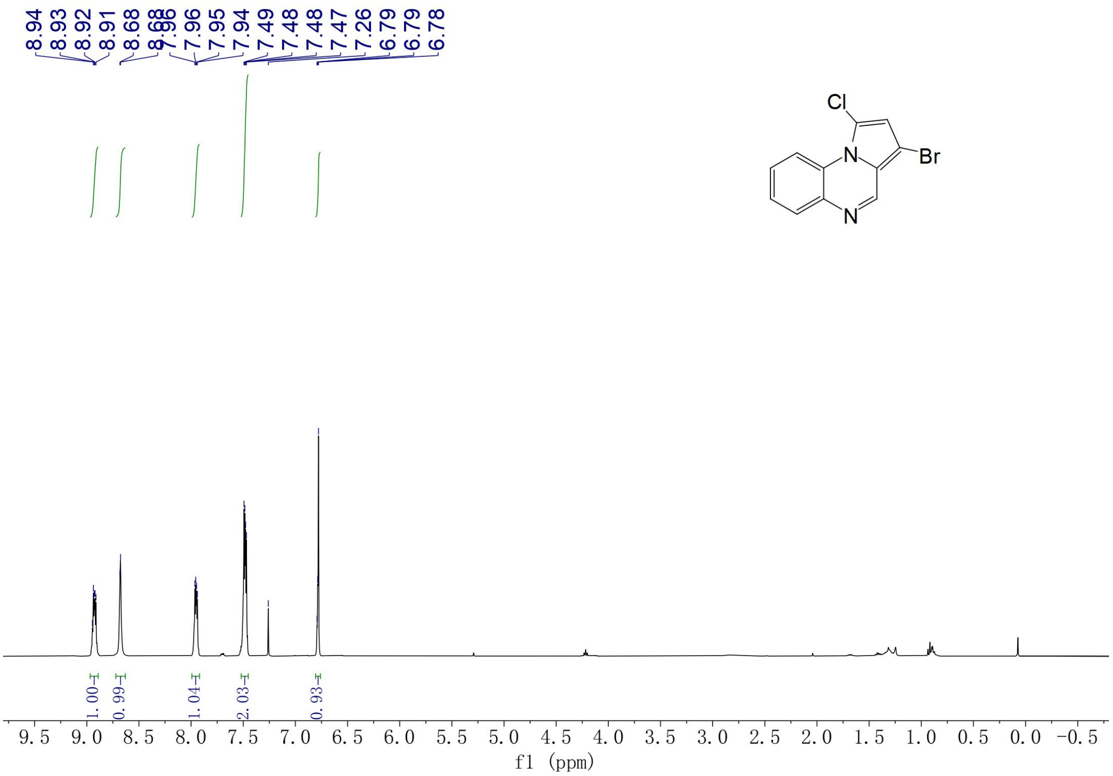

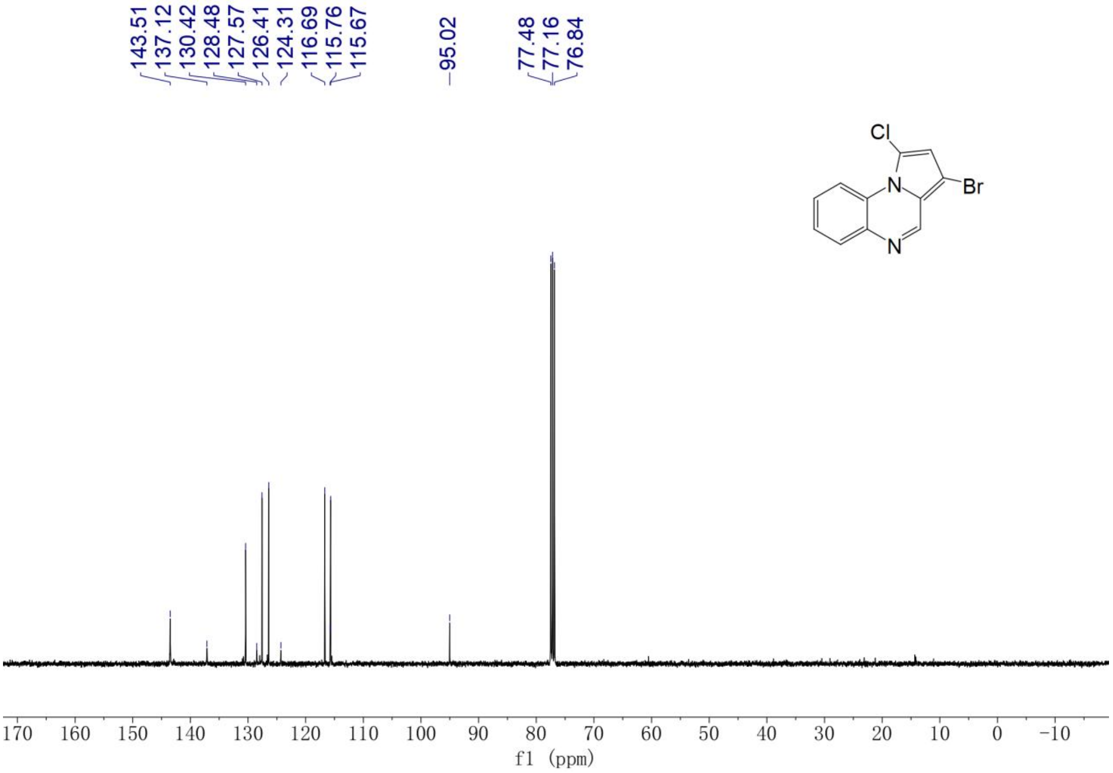

Supplement: RA-014-D4RA07358D-s001 [file RA-014-D4RA07358D-s001.pdf]
